# Autonomous and Continuous Atmospheric Water Harvesting Using Modified Wood

**DOI:** 10.1002/advs.75248

**Published:** 2026-04-13

**Authors:** Shiheng He, Jiaqi Su, Tianting Leng, Yujian Song, Jidong Dong, Pengfei Huo, Zhenhua Gao, Shuaiyuan Han

**Affiliations:** ^1^ State Key Laboratory of Woody Oil Resources Utilization Northeast Forestry University Harbin China; ^2^ College of Material Science and Engineering Northeast Forestry University Harbin China; ^3^ Engineering Research Center of Advanced Wooden Materials Ministry of Education Northeast Forestry University Harbin China; ^4^ Key Laboratory of Biobased Material Science and Technology Ministry of Education Northeast Forestry University Harbin China

**Keywords:** atmospheric water harvesting, autonomous and continuous harvesting device, heat induction desorption, poly(N‐isopropylacrylamide), wood modification

## Abstract

To overcome global water scarcity, it is necessary to develop technologies to provide efficient access to freshwater without geographical limitations. Sorption‐based atmospheric water harvesting (SAWH), a promising solution, presents limitations of low water uptake capacity of adsorbents, high energy consumption, and limited cycle stability. In this paper, a high‐performance SAWH composite material prepared by co‐incorporating a poly(N‐isopropylacrylamide)/polyacrylic acid (PNipam/PAA) network with lithium chloride (LiCl) into delignified wood (DW) is presented. In which the rigid, hydroxyl‐rich wood skeleton with graded channels serves as a multifunctional host to promote water transport and storage, while inhibiting thermal‐desorption‐induced structural deterioration of PNipam, thereby overcoming the cycling stability issue of PNipam/PAA‐LiCl in SAWH applications. Compared to other biomass adsorbents, the PNADW‐LiCl exhibits high water absorption (0.998 g g^−^^1^) and rapid sorption kinetics at 70% RH. Meanwhile, the material enables rapid and efficient water desorption at 80 °C and maintains stable performance over 10 consecutive absorption–desorption cycles. An automated, continuous, solar‐powered AWH device is further developed. A 24‐h real‐world outdoor (Harbin, China) test shows a water production of 2.631 L kg^−^^1^·day^−^^1^, which is much better than the reported method. This work provides valuable insights for designing next‐generation sustainable, efficient, and stable renewable atmospheric water harvesting materials and systems.

## Graphical abstract

A rigid, hydroxyl‐rich wood skeleton with graded channels is used as a multifunctional host to co‐incorporate a PNipam/PAA network and LiCl, creating a high‐performance sorbent for atmospheric water harvesting. The composite achieves rapid sorption, efficient low‐temperature desorption, and stable cycling, enabling a solar‐powered device with a high‐water production rate.

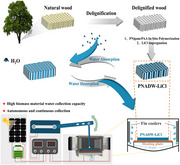

## 1 Introduction

Water shortage is one of the most urgent challenges today. The United Nations World Water Development Report 2025 points out that about 4 billion people suffer from periodic water shortages every year due to climate change [1], environmental pollution, and land desertification [2, 3]. Technologies, including wastewater treatment and desalination are adopted to obtain freshwater; however, high expenses and geographic constraints prevent their widespread use in poor arid regions [4, 5]. Atmospheric water harvesting (AWH) is considered a promising alternative method due to its ability to directly utilize water vapor in the air without geographical constraints [6, 7, 8, 9]. Among the three typical AWHs (fog capture [10, 11, 12], condensation device collection [13, 14, 15], and SAWH) [16, 17, 18, 19, 20, 21], SAWH has garnered significant attention due to its low energy consumption and high environmental adaptability [20, 22, 23]. However, traditional adsorbents such as zeolites [24], metal–organic frameworks (MOF) [25], and silica gel [26] exhibit poor water uptake and high temperatures to release water. Pure hygroscopic salts exhibit excellent water sorption properties but are prone to deliquescence and particle aggregation, resulting in poor cyclic stability [27]. Photothermal conversion additives can efficiently facilitate adsorbent desorption using solar energy with high energy conversion rates. However, such materials typically undergo only one sorption‐desorption cycle per day, resulting in relatively low overall water collection rates [28, 29, 30].

The incorporation of hygroscopic salts into the matrix can effectively enhance the matrix's water sorption capacity and prevent the deliquescence and aggregation of the salts [31, 32]. When the matrix is a thermoresponsive polymer, poly (N‐isopropylacrylamide) (PNipam), which exhibits hydrophilic‐to‐hydrophobic conversion transition near the low critical solution temperature (LCST), shows advantages in thermal desorption, enabling low‐temperature thermal desorption [33, 34, 35]. However, PNipam exhibits limited water absorption and lacks anchoring sites, making it difficult to immobilize LiCl. In contrast, PAA in PNipam‐co‐PAA enhances water uptake [36] and provides abundant carboxyl groups that coordinate with Li^+^ and form hydrogen bonds with Cl^−^ [37], effectively stabilizing LiCl within the polymer network. Even more troublesome is that the thermal desorption of PNipam leads to structural deterioration caused by polymer shrinkage, which severely declines its re‐sorption performance and triggers the release of hygroscopic salts from the PNipam, severely compromising its cycling stability [38, 39, 40]. Therefore, it is crucial to develop a rigid, porous, water‐absorbent, and low‐cost porous host matrix capable of inhibiting structural deterioration during the polymer thermal dehydration.

Natural wood, as a renewable porous material, possesses a graded pore structure and abundant hydroxyl groups, and stands as a promising candidate for a moisture‐sorbing matrix [41, 42, 43]. The high lignocellulose crystallinity makes the wood's pore structure rigid enough, ensuring it is stable enough to support polymers. Meanwhile, the abundant hydroxyl groups in wood can form strong hydrogen bonds with polymers, effectively stabilizing the polymer on the inner pore walls [44, 45]. However, the lignin in natural wood blocks pores, preventing effective interconnection within the wood's graded pore structures, reducing the wood's water capture capacity, hindering the introduction and loading of polymers, resulting in limited water uptake and high desorption energy consumption when used directly as an adsorbent [46, 47].

**FIGURE 1:**
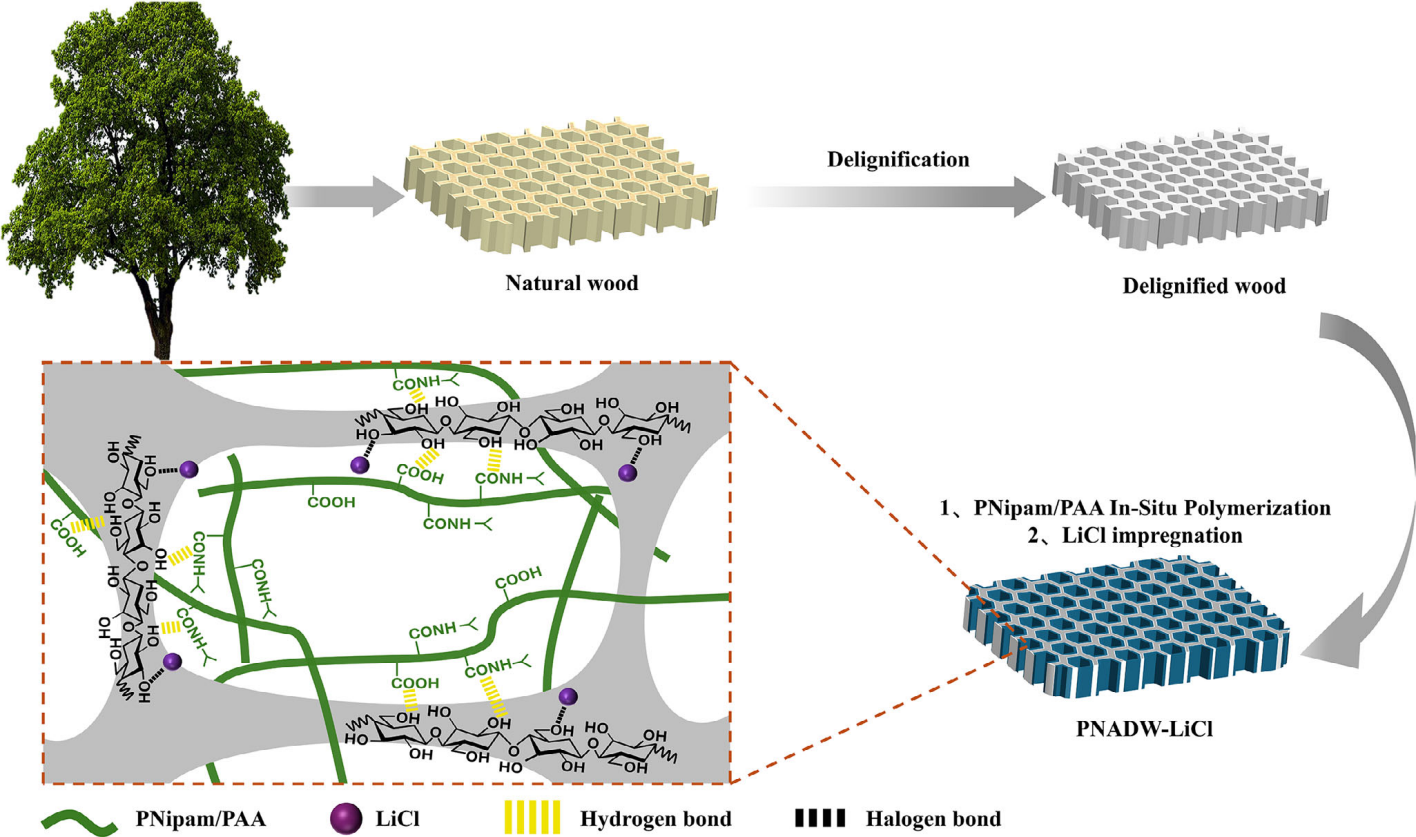
The preparation process of PNADW‐LiCl (modified wood).

Herein, we report a high‐performance SAWH composite material from modified natural wood, in which the rigid wood frame skeleton effectively inhibits the structural deterioration of PNipam during thermal cycling, enhancing the material's cyclic stability, while the loading of PNipam/polyacrylic acid (PAA) network and LiCl into delignified wood overcomes the wood's limitations of poor hygroscopicity and difficult desorption (Figure 1). The modified wood exhibits a moisture sorption of 0.998 g g^−^^1^ at 70% RH, while maintaining structural and performance stability after 10 sorption‐desorption cycles. To achieve autonomous and continuous atmospheric water harvesting, we developed a fully automated solar‐powered water collection system centered on modified wood, integrated with photovoltaic power generation, lithium‐ion battery energy storage, Joule thermal desorption, and a control system. This system completes 12 sorption‐desorption cycles per day, with the measured water collection rate is 2.631 L kg^−^^1^·day^−^^1^ (verified outdoors from 8:00 AM on September 25 to 8:00 AM on September 26 at Northeast Forestry University in Harbin). In summary, this study effectively addresses the poor cycling stability of thermal‐responsive polymer during the sorption‐desorption cycles, overcomes the limitations of natural wood for efficient SAWH, develops a continuous atmospheric water collection system, and provides new insights for designing sustainable, efficient, stable, and renewable next‐generation atmospheric water harvesting materials.

## 2 Results and Discussion

### 2.1 Characterization of Modified Wood

**FIGURE 2:**
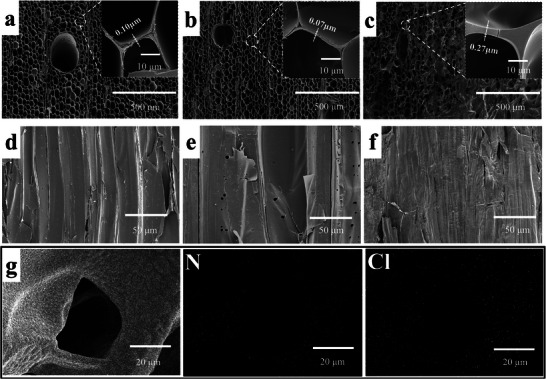
Cross‐sectional SEM image of (a) NW, (b) DW, and (c) PNADW; Longitudinal cross‐sectional SEM images of (d) NW, (e) DW, and (f) PNADW; (g) SEM image and EDS elemental mapping image of PNADW‐LiCl.

The robust and stable interconnected graded porous structure of modified wood is the foundation for stably loading polymers and hygroscopic salts, enabling the development of highly efficient SAWH materials. SEM images (Figure 2a–f) visually demonstrate the variations of pore structure and morphology of NW, DW, and PNADW. Figure 2a,d reveal that NW exhibits naturally anisotropic porous structures with smooth cell walls, which are unfavorable for polymer loading. After delignification, the wood block changed from brown to white (Figure S1). Figure 2a,b reveals that the cell walls of delignified wood have significantly thinned (from 0.10 to 0.07 µm), with markedly enlarged intercellular gaps, creating a more favorable environment for polymer loading. Notably, the delignification process resulted in the creation of abundant transverse micropores in the vessel walls (Figure 2e). The transverse micropores provide a structural foundation for the formation of interconnected polymer networks in DW, thereby further preventing thermally induced structural deterioration of the polymer. The SEM image of the PNADW cross‐section (Figure 2c) demonstrates that the gaps between cell walls have been completely filled with polymer, resulting in an increase in cell wall thickness from 0.07 to 0.27 µm, and further confirming the successful loading of polymer onto the wood cell walls. The SEM image of the PNADW longitudinal section (Figure 2f) shows that the gaps between vessels have disappeared, and the transverse micropores on the vessel walls are no longer visible, indicating that the polymer has formed an interpenetrating structure with DW. The SEM image of PNADW‐LiCl (Figure 2g) reveals that numerous uniformly distributed micro‐protrusions appeared on the smooth polymer‐coated microporous wall surface after LiCl immersion. EDS mapping of N and Cl clearly shows that the polymer and LiCl are distributed homogeneously on the microporous walls. Based on the elemental proportions of N and Cl, the polymer and LiCl accounts were calculated to be 56.94% and 12.31% in PNADW‐LiCl, exhibiting a high loading capacity. Pore size statistics (Figure S2) show that the pore sizes of both PNADW and PNADW‐LiCl are predominantly concentrated around 30 µm.

**FIGURE 3:**
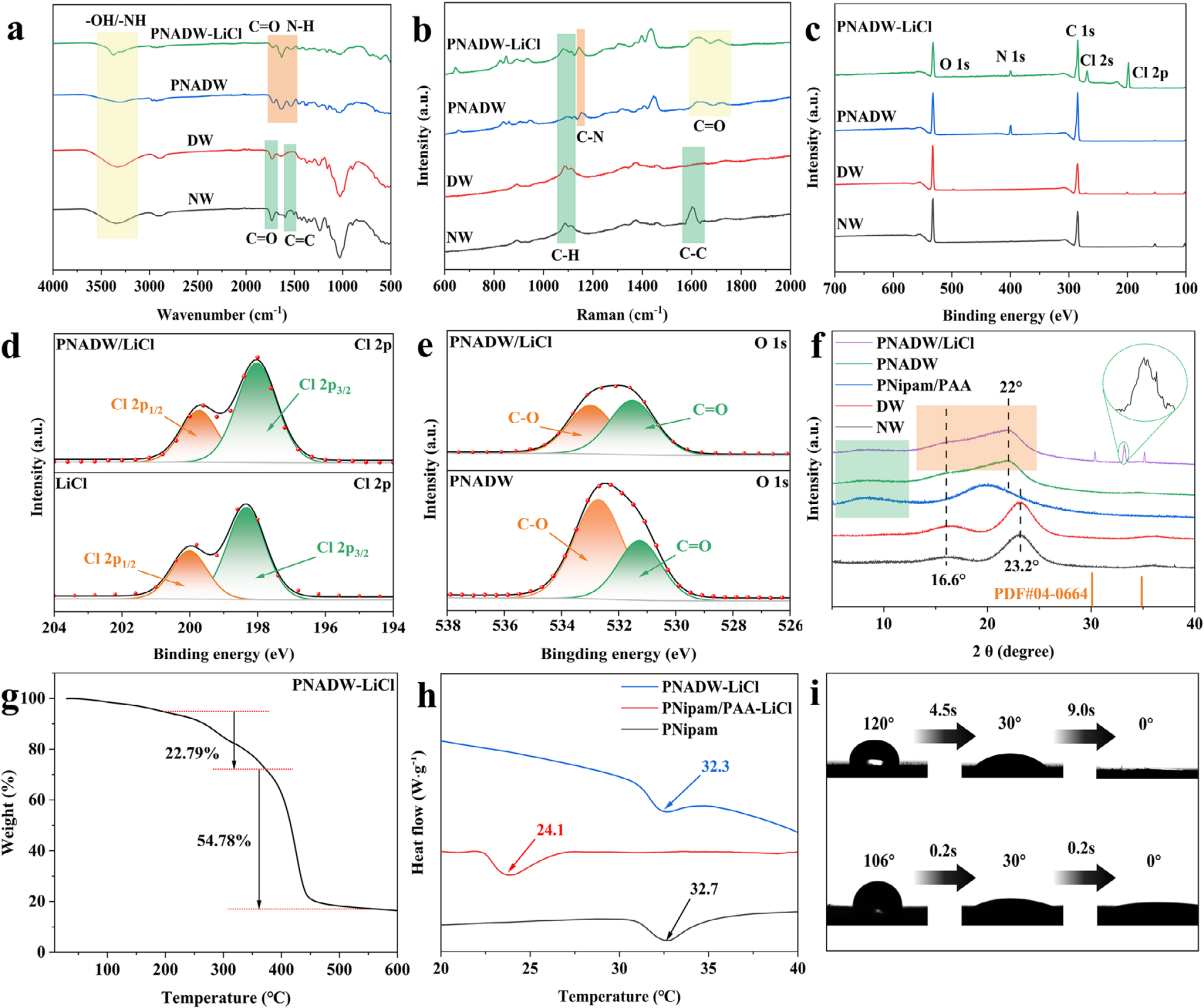
(a) FT‐IR spectra of NW, DW, PNADW, and PNADW‐LiCl; (b) Raman spectra of NW, DW, PNADW, and PNADW‐LiCl; (c) XPS spectra of NW, DW, PNADW, and PNADW‐LiCl; (d) High‐resolution XPS spectra of Cl 2p for PNADW‐LiCl and LiCl; (e) High‐resolution XPS spectra of O 1s for PNADW‐LiCl and PNADW; (f) XRD patterns of LiCl, NW, DW, PNADW, and PNADW‐LiCl; (g) TGA curve of PNADW‐LiCl; (h) DSC curves of PNipam, PNipam/PAA‐LiCl and PNADW‐LiCl; (i) Dynamic contact angle of NW and PNADW‐LiCl.

During wood modification, changes in elemental composition, functional groups, and crystalline morphology occurred, while new interactions also appeared between functional groups. Spectral analyses were used to confirm lignin removal, validate the successful loading of polymer/lithium chloride, and reveal the mechanism of the modified wood's stable structure and high sorption efficiency. FT‐IR spectra (Figure 3a) reveal that, compared to NW, the disappearance of C═C peaks at 1593 and 1505 cm^−^^1^ and the reduced intensity of the C═O peak at 1733 cm^−^^1^ in DW confirm the successful removal of lignin and partial removal of hemicellulose. After polymer introduction, PNADW shows peaks at 1450 cm^−^^1^ (C─N bond), 1540 cm^−^^1^ (N─H bond), 1634 cm^−^^1^ (amide C═O bond), and 1733 cm^−^^1^ (carboxyl C═O bond), almost correspond to the peaks of PNipam/PAA (Figure S3), in which the slight shifts of N─H and C═O peaks are attributed to the formation of hydrogen bond, confirming the polymer has been successfully incorporated into WD. Following the introduction of LiCl, the coordination of lithium ions competes with the existing hydrogen bonds in the system, leading to the disruption of some hydrogen bonds in PNADW. In the FT‐IR spectrum, broadening and blue shift of the O─H/N─H hydrogen bond stretching vibration band are observed, with a sharp peak appearance at 3380 cm^−^^1^, corresponding to the dissociative N─H vibration. Meanwhile, the asymmetric stretching vibration peak of the carboxylate (～1570 cm^−^^1^) shifts to a lower wavenumber (redshift) due to the strong interaction between Li^+^ and ─COO^−^. The above changes indicate that LiCl participates in and reorganizes the original intermolecular interactions, forming a new network.

As a supplement to FT‐IR, the Raman spectrum (Figure 3b) further confirms the complete removal of lignin (disappearance of C─C peak at 1600 cm^−^^1^) and partial removal of hemicellulose (decrease of C─H peak at 1385 cm^−^^1^). Notably, the C─H bending vibration at 1130 cm^−^^1^ and the cellulose skeleton vibration peak at 1100 cm^−^^1^ in the DW sample prove the survival of cellulose from delignification. Following polymer incorporation, PNADW exhibited distinct C─N peaks at 1155 cm^−^^1^, carboxyl C═O peaks at 1632 cm^−^^1^, and amide C═O peaks at 1723 cm^−^^1^, corresponding to the peaks in pure PNipam/PAA (Figure S4). The tiny shift of the C═O peak is due to the formation of hydrogen bonds between PNipam/PAA and cellulose, further confirming the successful loading of PNipam/PAA into DW. Similar to FT‐IR, the introduction of LiCl caused a significant shift of C═O peak in PNADW‐LiCl (from 1723 to 1708 cm^−^^1^), since LiCl reorganized the intermolecular interaction network.

XPS was employed to analyze the elemental composition and bonding states of the samples. Since lignin contains more carbon than cellulose, the XPS results (Figure 3c–e) combined with elemental analysis (Table S1) indicate that the carbon content of DW decreased by 1.5% compared to NW, further confirming the removal of lignin. In the XPS spectrum of PNADW‐LiCl, the O 1s, N 1s, C 1s, and Cl 2p peaks are located at 532, 400, 285, and 198 eV respectively, which the N1s peak matches PNipam/PAA (Figure S5), while the C 1s and Cl 2p peaks align with those of LiCl, indicating the successful loading of PNipam/PAA and LiCl onto the sample. The high‐resolution Cl 2p spectra of LiCl and PNADW‐LiCl (Figure 3d) revealed characteristic peaks of Cl 2p3/2 and Cl 2p1/2 at 197.9 and 199.8 eV with a 2:1 intensity ratio, which matches the chemical state of Cl^−^ in LiCl. Furthermore, compared with LiCl_2_, the Cl 2p peak of PNADW‐LiCl shifted toward lower binding energy, which is attributed to the formation of hydrogen bonds between Cl^−^ and the PNipam/PAA polymer network. The high‐resolution Cl 2p spectra of PNADW‐LiCl (Figure 3d) revealed characteristic peaks of Cl 2p_3/2_ and Cl 2p_1/2_ at 197.9 and 199.8 eV with a 2:1 intensity ratio, which matches the chemical state of Cl^−^ in LiCl, providing strong evidence for the successful loading of LiCl. High‐resolution O1s spectra of PNADW and PNADW‐LiCl (Figure 3e) reveal that both O1s contain two components at 532.7∼532.9 eV (C─O bonds) and 531.3∼531.5 eV (C═O bonds). Notably, with loading LiCl, the electron of Li was continuously attracted by O, resulting in an increase in electron cloud density, which led to a slight increase in C─O binding energy and a decrease in peak intensity, indicating strong interactions between LiCl and the polymer.

The high crystallinity of cellulose is fundamental to maintaining its high strength. XRD (Figure 3f) was employed to investigate changes in crystallinity during the modification process of wood. DW exhibits crystallographic diffraction peaks for cellulose type I at 16.6° and 23.3°, with peak intensity even higher than that of NW, which indicates that the delignification process not only kept the highly crystalline structure of cellulose but also elevated the overall crystallinity index of the material due to the removal of amorphous components. After the introduction of PNipam/PAA, the PNADW diffraction peak shifted from 23.2° to 22° and broadened, because the strong hydrogen‐bonding interactions between polymer and hydroxyl groups on cellulose (200) plane increase the interplanar d‐spacing, reducing the diffraction angle (θ). This provides strong evidence for hydrogen bonding between the polymer and DW. Meanwhile, the crystalline cellulose peak at 16.6° was masked by broad signals from the amorphous PNipam/PAA (humps at 5°–13° and 15°–25° in the XRD (Figure S6)). After the introduction of LiCl, PNADW‐LiCl emerges with three new diffraction peaks at 30.4°, 33.3°, and 35.2° in addition to the original PNADW signals. The peaks at 30.4° and 35.2° are indexed to the (111) and (220) planes of LiCl. Notably, the peak at 33.3° is not typical in pure LiCl. This peak can be further identified as three components: the (202) and (220) planes of LiCl·H_2_O, and a novel, short‐range ordered “ion‐polymer composite”. This composite is formed by Li^+^ coordination to the COO^−^/COOH of PAA and C═O of PNipam, providing clear evidence that LiCl is strongly bound to the polymer and not merely blended.

Thermal analysis was used to characterize the thermal stability and thermal responsiveness of modified wood. TGA (Figure 3g) shows that PNADW‐LiCl exhibits no significant weight loss below 100 °C (less than 3% weight loss attributable to incompletely dried water), which indicates that no structural failure occurs due to thermal degradation. Weight loss of 22.79% and 54.78% occurs at 373 °C and 552 °C, respectively, due to the degradation of cellulose and polymer breakage. The 16% final residue consists of carbonized PNADW and un‐gasified LiCl. The DSC curves (Figure 3h) of PNipam, PNipam/PAA‐LiCl, and PNADW‐LiCl exhibit distinct endothermic peaks, which are attributed to the dehydration process occurring at the LCST. Pure PNipam exhibited an LCST of 32.7 °C, consistent with literature values. The LCST of PNipam/PAA‐LiCl was observed at 24.1 °C, which is attributed to the combined effects of PAA–PNipam interactions and the strong salting‐out effect of LiCl promoting chain dehydration. Strangely, the observed LCST of PNADW‐LiCl is 32.5 °C (the double check result shown in Figure S7), close to pure PNipam. This is because the hydrophilic scaffold, DW, dilutes and partially shields the intermolecular interactions between PAA and PNipam. Additionally, the DW scaffold binds LiCl, weakening its salting‐out effect on PNipam. As a result, the local chain environment of PNipam is restored to a state closer to its intrinsic state, leading to an LCST similar to pure PNipam. Consequently, heating the sample to 32.4 °C achieves efficient dehydration, below its thermal decomposition temperature.

More importantly, water sorption capacity, the core property as a SAWH material, has been significantly enhanced during the modification of wood. Dynamic water contact angle experiments (Figure 3i) indicate that water droplets are rapidly absorbed upon contacting the PNADW‐LiCl surface within 0.4 s, significantly shorter than the 9.5 s observed for NW. Notably, with the modifications, the mechanical strength of PNADW‐LiCl was also enhanced compared to DW. This improvement was attributed to the filling of polymer and LiCl into the intercellular pores, as well as the structural support provided by the polymer network. Three‐point bending test (Figure S8) shows that PNADW‐LiCl exhibits a bending strength 1.71 times higher than NW and 5.13 times higher than DW, further ensuring the structural stability of PNADW‐LiCl during absorption–desorption cycles.

### 2.2 Absorption and Desorption Properties of Modified Wood

**FIGURE 4:**
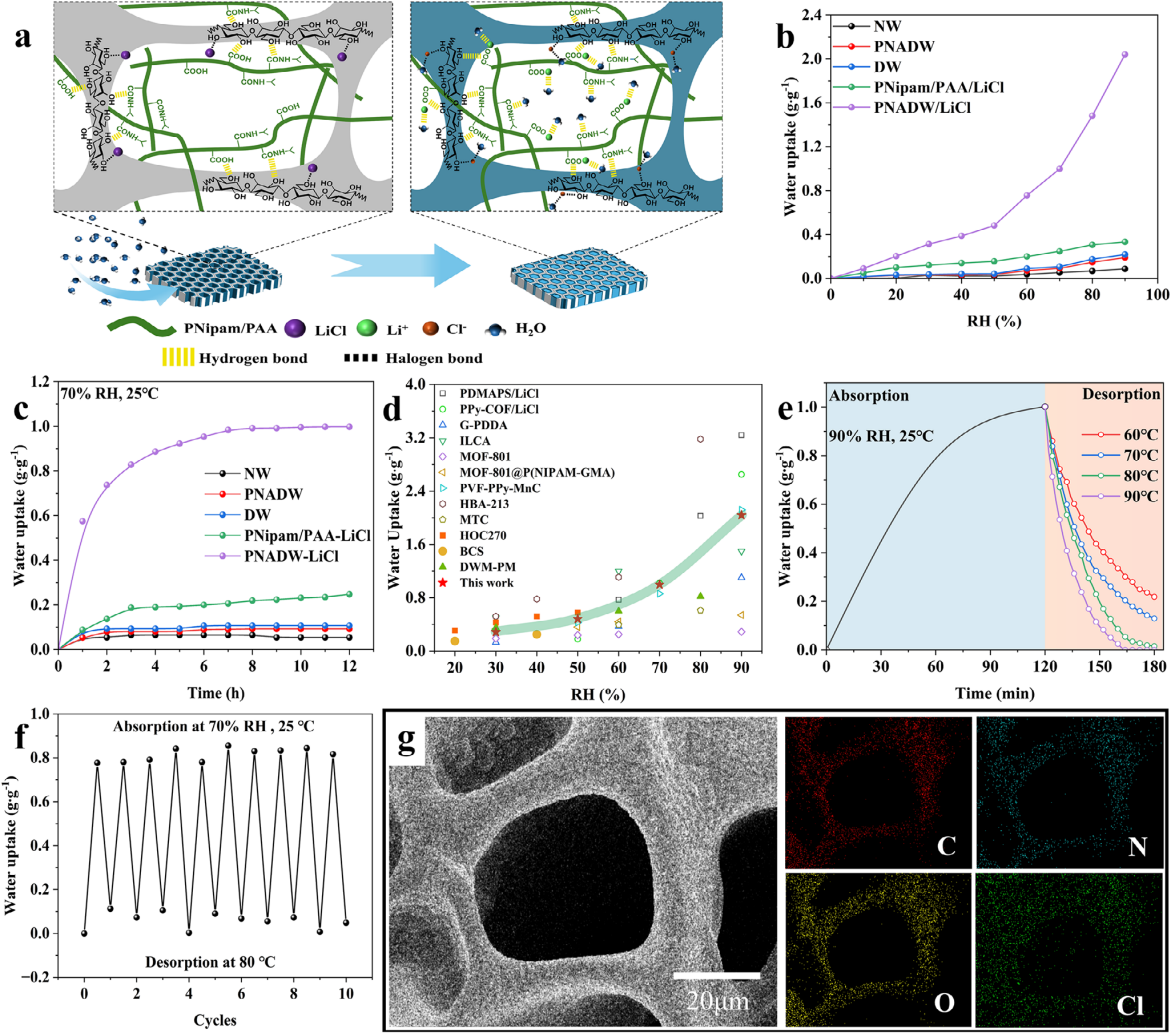
(a) Schematic illustration of moisture absorption by PNADW‐LiCl; (b) Static sorption of NW, PNADW, DW, PNipam/PAA‐LiCl, and PNADW‐LiCl at 25 °C from 10% to 90% RH; (c) Dynamic water absorption of NW, PNADW, DW, PNipam/PAA‐LiCl, and PNADW‐LiCl at 25 °C and 70% RH; (d) Water uptake of PNADW‐LiCl compared with other literature reports; (e) Desorption properties of PNADW‐LiCl at different temperatures; (f) PNADW‐LiCl 10‐cycle durability test; (g) SEM image and EDS (C, N, O, and Cl elements) images of PNADW‐LiCl wood after 10‐cycling.

The large number of hydrophilic groups (including hydroxyl (─OH), carboxyl (‐COOH), amid (‐CONH‐) groups, and so on) in modified wood PNADW‐LiCl provides excellent water‐harvesting properties due to hydrogen bonding, electrostatic interactions, and dipole interactions. The water sorption capacity is further enhanced by loading hygroscopic salt LiCl. The stable 3D porous framework possessed by DW further enhances the water absorption performance. First, the abundant microporous structure of the framework enhances capillary action, leading water molecules rapid transport; Second, the framework provides robust support for the polymer network and hygroscopic salt, while simultaneously increasing their contact area with air, significantly improving the efficiency of moisture capture from the atmosphere; Finally, the high stability of the framework and the robust interactions between polymer/LiCl and framework prevent the decline in adsorption caused by polymer structural deterioration. In one word, this synergy between the material's functional group and structure is key to the high‐efficiency water absorption (Figure 4a).

The static water absorption experiment (Figure 4b) demonstrates that the water uptake of PNADW‐LiCl increases with humidity increase (0.091 g g^−^^1^ at 10% RH to 0.998 g g^−^^1^ at 70% RH, and 2.041 g g^−^^1^ at 90% RH). PNADW‐LiCl demonstrates significantly superior moisture sorption performance compared to the control materials across the entire humidity range. Compared with PNipam/PAA‐LiCl, PNADW‐LiCl shows a two‐ to six‐fold increase in moisture absorption (10%–90% RH), due to the effect of water adsorption, transport, and storage caused by the structure difference. PNipam/PAA‐LiCl can absorb some moisture via polymer‐LiCl synergy; however, its low porosity and small specific surface area limit both the rate and total capacity of moisture uptake. The sorption is more distinct at high humidity, as after the polymer and LiCl in PNADW‐LiCl reach water saturation, water can rapidly transfer further through the internal microporous structure into the cellulose voids. PNADW and DW exhibit comparable moisture absorption, both outstanding than NW and much worse than PNADW‐LiCl, which is consistent with the contact angle test results (Figure S9). Delignification enhances the water absorption of wood by exposing more hydroxyl groups. Polymer loading can introduce hydrophilic groups, while the original hydroxyl on DW was covered, leading to unchanged water absorption. However, PNADW‐LiCl exhibits excellent overall moisture absorption, indicating that the water absorbed by LiCl can be effectively transferred and stored in the cellulose matrix.

The dynamic absorption experiments (Figure 4c; Figure S10a–c) reveals that PNADW‐LiCl exhibits highest moisture absorption rates during the initial two hours (0.368, 0.058, 0.187, 0.492 g·g^−^^1^·h^−^^1^), it also maintains the highest moisture absorption capacity (0.736 g g^−^^1^ at 70% RH) after reaching absorption equilibrium compared to all control samples. Based on the dynamic‐static water absorption experiment results, the water absorption mechanism of PNADW‐LiCl can be inferred as follows: The internal pores of the wood increase the contact area between LiCl/polymer and air, while capillary action promotes atmospheric moisture adsorption. Subsequently, LiCl chemically adsorbs water to form LiCl · H_2_O. And then LiCl · H_2_O continues to absorb water, gradually transforming into a salt solution. The water in solution then diffuses through the polymer matrix into the interfibrillar spaces of cellulose and micropores for storage. Finally, LiCl recrystallizes from the solution and re‐enters the hygroscopic cycle. Compared with other SAWH materials reported in the literature (Figure 4d; Table S2), PNADW‐LiCl exhibits outstanding water absorption capacity even relative to some non‐naturally modified materials, demonstrating its potential for application in highly efficient atmospheric water harvesting.

As evidenced by the dynamic and static absorption results above, the introduction of LiCl can significantly enhance the water uptake of PNADW. However, the relationship between LiCl content and water uptake (Figure S11) indicates that higher LiCl loading does not mean high water uptake. With the concentration of LiCl solution used for soaking PNADW increased from 5 to 15 wt.%, the water uptake of PNADW‐LiCl increases and then declines, reaching a maximum value (0.779 g g^−^^1^) at 10 wt.%. This is mainly because LiCl effectively enhances the material's hygroscopicity due to its high hydration energy and low vapor pressure at low LiCl loading. Conversely, soaking PNADW in a high‐concentration LiCl solution causes LiCl to exceed its loading limit, leading to aggregation and clumping, thereby reducing the overall water uptake. Notably, the water uptake performance of PNADW‐LiCl is also significantly influenced by the monomer composition ratio of the polymer and its loading amount. As shown in Figure S12, when the molar ratio of PNipam to PAA is 1:1, and the initial polymer concentration is 1.105 mol/L, PNADW‐LiCl shows the best water uptake performance, reaching 0.786 g g^−^^1^ in 2.5 h. Since excessive polymer can block the micropores of DW, insufficient loading or inappropriate monomer ratio hinders the effective loading of LiCl, thereby reducing water absorption capacity. Based on the above, the monomer used for DW modification in this paper is a mixed solution of 1.105 mol/L Nipam and AA (mole ratio 1:1), and the solution used for PNADW soaking is a 10 wt.% LiCl solution, unless otherwise noted.

Desorption efficiency is the key property for SAWH materials. The TGA curve of PNADW‐LiCl (70% RH adsorbed 1 h, with 55% water content) (Figure S13) shows that 80% of the water is removed below 90 °C, indicating that most free water in PNADW‐LiCl can be easily removed. The remaining bound water is difficult to desorb, even when the temperature increases a lot. The desorption process originates from the contraction behavior of PNipam segments when the temperature exceeds their LCST. The DSC result (Figure 3h) indicates that the LCST of PNADW‐LiCl is 32.4 °C. However, the desorption curve at 40 °C (Figure S14) indicates that PNADW‐LiCl only lost 8 wt.% water after 1 h heating at 40 °C, which means temperature just above LCST is not enough for efficient desorption. This is because more energy is still needed to overcome the viscous resistance of polymer chain motion and generate sufficient contraction force to resist the expansion pressure of the PAA network. Therefore, the actual desorption process needs a much higher temperature than the LCST. Based on the above, isothermal desorption experiments were used to investigate the PNADW‐LiCl desorption performance at 60 °C, 70 °C, 80 °C, and 90 °C. As shown in Figure 4d, PNADW‐LiCl exhibits high desorption efficiency over a wide temperature range. The desorption rate shows a positive correlation with temperature. Within 30 min, the sample lost 90.4 wt.% water at 90 °C, 80.3 wt.% water at 80 °C, 68.8 wt.% water at 70 °C, and 58.3 wt.% water at 60 °C. When focusing on a specific temperature, the sample exhibits a decreasing desorption rate over time.

Good cycle stability is a prerequisite for the practical application of SAWH materials. The absorption–desorption cycling experiment was used to evaluate the performance stability of PNADW‐LiCl; meanwhile, its structural stability was verified through morphological observation and elemental analysis. The 10‐cycle durability test of PNADW‐LiCl (Figure 4e) demonstrates that during the cycle process of water sorption at 25  °C (70% RH) and dehydration at 80 °C, fluctuations in material absorption capacity remains within 10% (ranging from 0.777 to 0.855 g g^−^^1^), and desorption efficiency is above 90% (91.35% to 99.35%). Moreover, no degradation in absorption and desorption performance is observed after 10 cycles. Macroscopic morphology analysis (Figure S15) shows that there are no significant changes in volume, color, or other visual aspects compared to the pristine PNADW‐LiCl after 10 cycles. SEM images of PNADW‐LiCl after 10‐cycle (Figure 4f) confirm that the microporous structure remains intact without collapse, and the tiny protrusions on the microporous surface were also retained. EDS indicates that LiCl remains distributed on the micropores’ surface without agglomeration or loss after multiple cycles. In summary, the stable, graded porous structure of PNADW‐LiCl effectively maintains the loading state of both the polymer and LiCl, thereby ensuring its outstanding cycling stability.

### 2.3 Solar‐Powered, Continuous Atmospheric Water Harvesting Collection System Design and Outdoor Water Harvesting

In recent years, numerous SAWH devices have been developed and tested in practical applications. However, most of these devices are manual and can only perform one absorption–desorption cycle per day. Although the device reported by Zhang [48] achieves automated water collection and can complete several cycles per day, its outdoor cycling experiments are limited to daytime due to the photo desorption properties of its SAWH material, preventing utilization of the higher humidity water vapor present at night. To fully utilize nocturnal water vapor—enabling 24‐h water collection—we abandoned the concept of introducing a photothermal agent into the DW during the initial design stage. Instead, we engineered the material for low‐temperature desorption, ensuring efficient desorption even during light‐free nights. Based on our SAWH material, we have designed a solar‐powered, continuous atmospheric water harvesting collection system. The device consists of three main components: an absorption–desorption chamber, a control system, and an energy supply unit. For detailed equipment specifications, refer to the experimental section.

**FIGURE 5:**
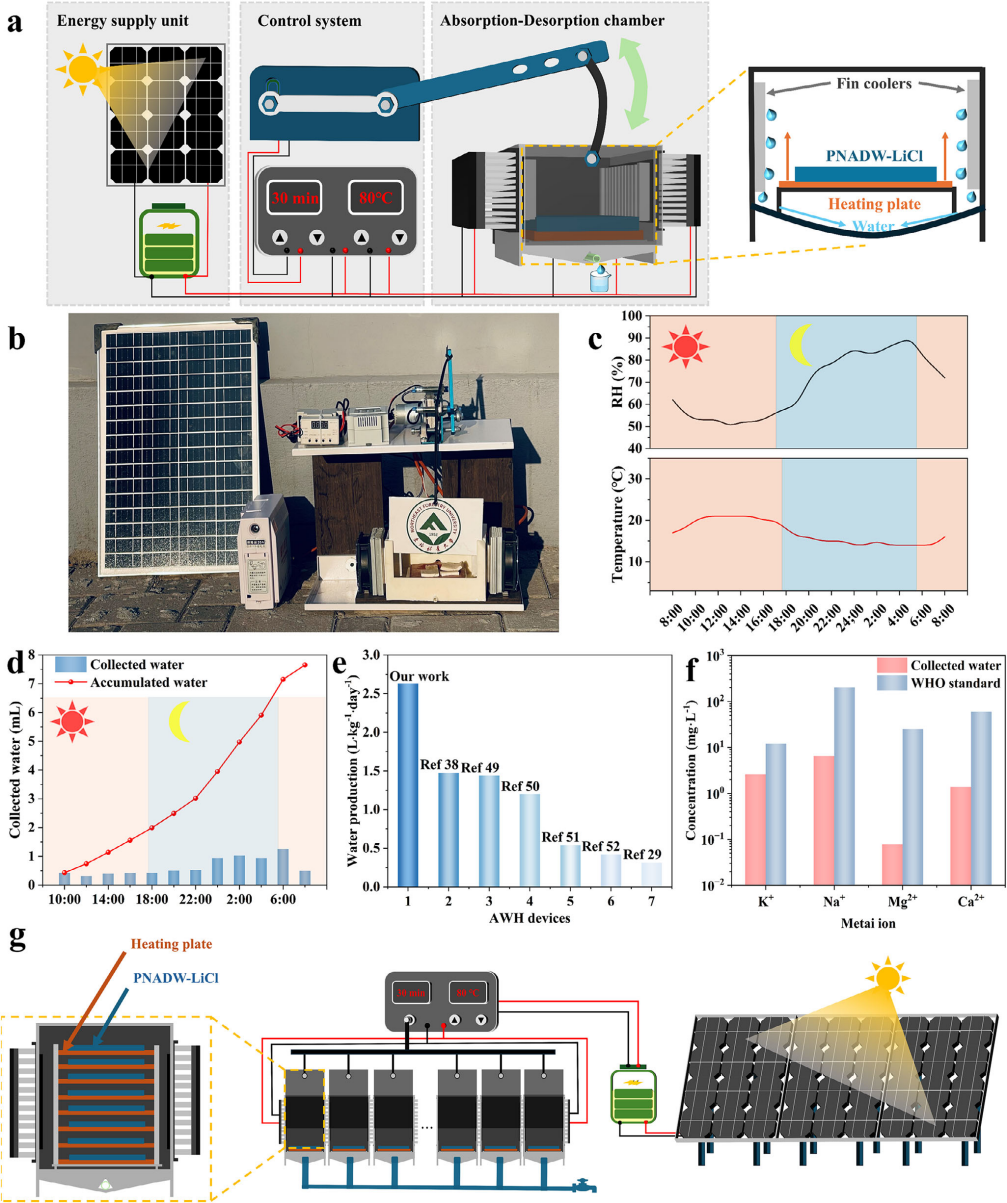
(a) Schematic diagram of the automatic continuous atmospheric water harvesting collection system; (b) Photograph of the SAWH system under outdoor water harvesting test; (c) Environmental temperature and humidity variations during outdoor testing; (d) Water collection from 8:00 on September 25 to 8:00 on September 26, 2025; (e) Comparison of the practical water productivity between the previously reported biomass based SAWH materials and our modified wood; (f) Ion concentration of collected water and comparison with WHO drinking water standards; (g) Conceptual diagram of the modular AWH device.

Selecting a suitable sorption‐desorption condition is a tricky issue. The temperature and humidity for sorption depend on the outdoor environment in Harbin summer (Figure 5c) (approximately 20°C, 50%RH～90%RH). Sorption time selection accorded to the absorption tests results of the constant temperature (25 °C) and constant humidity (50%, 70%, and 90%) (Figure S10a,b; Figure 4c), the water absorption rate decreased significantly after 2 h, and absorption continued slowly even after 12 h. Considering the balance between single‐cycle water absorption and the absorption cycles (2.947 mL over 120 min × 4 cycles is less than 3.238 mL over 90 min × 5 cycles), 90 min was selected for one‐time absorption. Desorption curves at different temperatures (Figure 4d) were used to guide the selection of desorption conditions. The desorption curve clearly shows that the desorption efficiency increases with rising temperature; however, higher temperature means more energy consumption and may damage the material's cycle stability. The difference in absorption efficiency between 80 °C and 90 °C is less than 5%, and maintaining 90 °C requires at least 20% more energy input, the desorption at 80 °C was ultimately selected. The desorption rate at 80 °C decreases over time (68.8% desorbed in the first 30 min and only 18.2% in the subsequent 30 min). Considering absorption efficiency and energy consumption, one‐time desorption took 30 min. Finally, an automatic, continuous, atmospheric water collection device is deployed outdoors based on the above conditions, serving to validate the water collection efficacy of our modified wood and the automated water collection system under real‐world conditions.

The outdoor water collection of modified wood PNADW‐LiCl with our automatic absorbent device was evaluated under natural environment at Northeast Forestry University in Harbin, China, from September 25 to 26, 2025. At first, the pre‐dried PNADW‐LiCl (2.903 g) was placed in the desorption chamber, and the chamber door was opened to the atmosphere. After 90 min of water absorption, the chamber door was closed, and then the sample was heated to 80 °C while the cooler within the desorption chamber cooled and kept desorbing for 30 min to complete the first absorption–desorption cycle. The process continued for 24 h, 12 absorption–desorption cycles were completed. The water harvesting device and the measured outdoor temperature and humidity during the period are depicted in Figure 5b,c. The temperature‐humidity curves indicate the daytime environment is approximately stable at (20 °C, 50% RH), while the temperature decreases and humidity increases are maintained around (15 °C, 80% RH) at night. Notably, at 4:00 a.m., the humidity even reaches as high as 90% RH. Therefore, it is particularly important for actual atmospheric water collection to utilize nighttime, as both higher humidity and lower temperatures favor atmospheric water harvesting. The 12‐cycle water collection (Figure 5d) clearly shows that the total water collection in 6 cycles during the daytime is 2.896 mL, which is much lower than the water collection in 6 cycles at night (4.757 mL), further demonstrating the necessity of utilizing nighttime for water collection. Benefiting from nighttime water absorption, our modified wood achieves 12 adsorption‐desorption cycles per day. After completing 12 cycles over 24 h, the cumulative amount of water collected is 7.653 mL, equivalent to 2.631 L kg^−^^1^·day^−^^1^, which is much higher than other reported biomass SAWH materials (Figure 5e) [29, 38, 49, 50, 51, 52]. Water quality testing (Figure 5f) shows that the concentrations of K^+^, Na^+^, Mg^2+^, and Ca^2+^ in the collected water are all below the WHO standards for high‐quality drinking water. It is noteworthy that, compared to the ideal state of absorption and desorption, the actual water collection of our modified wood only reaches half of the theoretical capacity, which means this system still has significant potential.

For practical applications, the material's scalability, the desorption chamber's adaptability to large amounts of material, the water uptake capacity in harsh environments, and the ability to adjust water uptake on demand are also important. 24 h water collections of two‐fold (5.914 g) and five‐fold (14.571 g) AWH woods (Figure S16) show that the daily water uptake is 15.888 mL and 40.331 mL, which is proportional to the amount of AWH wood used, confirming the material's scalability. The water collection using a two‐layer adsorption‐desorption chamber with twice the materials (Figure S17) shows that the multilayer design can use space efficiently without sacrificing water collection capacity. Furthermore, water collection under dry conditions (Figure S18) indicates that the material's water uptake capacity can still reach 0.360 L kg^−^^1^·day^−^^1^ even the average daily relative humidity is 34% RH, while under high‐humidity conditions (Figure S19), it can achieve as high as 3.079 L kg^−^^1^·day^−^^1^. For real‐world applications, a modular assembly concept is proposed. In this concept, multilayer adsorption‐desorption chambers equipped with appropriate amounts of adsorbent serve as adsorption modules, while solar power stations serve as power supply modules. A number of adsorption modules can be connected to form a water‐harvesting facility, depending on the actual water demand (Figure 5g).

## 3 Conclusion

In summary, a wood‐based high‐performance atmospheric water harvesting composite material and a supporting solar‐powered fully automatic water collection system were developed. Through delignification, the micropores in wood were interpenetrated and exposed more hydrophilic hydroxyl groups, thereby forming an ideal matrix for water molecule transport and storage. The thermo‐responsive PNipam/PAA were attached to the microporous walls in DW robustly via mechanical interlocking and hydrogen bonding. Meanwhile, LiCl was bound with both the polymers and the DW through coordination interactions, hydrogen bonds, and halogen bonds. The rigid DW cellulose skeleton prevented the thermal‐desorption‐induced structural deterioration of PNipam and suppressed the dehydration‐induced aggregation of LiCl, thereby endowing the material with excellent stability. In other words, our study resolved not only the poor water sorption and difficult desorption of wood as SAWH materials, but also the poor cycling stability of polymer‐LiCl. The modified wood exhibited a water absorption of 0.998 g g^−^^1^ at 70% RH, which could be rapidly desorbed at 80 °C–90 °C and maintained stable performance after 10 cycles. In real‐world outdoor conditions, the modified wood combined with a special fully automated water collection device, achieved a water production efficiency of 2.631 L kg^−^^1^·day^−^^1^ with water quality meeting drinking standards. This work not only provides an innovative solution to the problem of poor cycling stability of thermally responsive SAWH materials but also demonstrates a new paradigm for utilizing wood. Furthermore, the collaborative design of “material‐device” demonstrates the practical application potential of modified wood in sustainable water harvesting.

## 4 Experimental Section

### 4.1 Preparation of Delignified Wood (DW)

10 mm × 10 mm × 5 mm natural wood (NW) slices were soaked in a 5 wt.% sodium hypochlorite (NaClO_2_) solution and kept at 80 °C for 8 h. Wash the slices three times with anhydrous ethanol, then soak in deionized water for 12 h to remove residual chemicals, yielding delignified wood (DW).

### 4.2 Preparation of PNipam/PAA Aerogels

Nipam (1.105 mol/L, 2.501 g), AA (1.105 mol/L, 1.510 mL), MBA crosslinker (0.025 mol/L, 0.154 g), and AIBN initiator (0.025 mol/L, 0.082 g) were dissolved in DMF (20 mL) to prepare a uniformly Nipam/AA mixture solution. The mixed solution was polymerized at 80 °C for 2.5 h, then soaked in deionized water to remove unreacted monomers and solvents. Freeze‐dry to constant weight to obtain the PNipam/PAA aerogel.

### 4.3 Preparation of PNipam/PAA Modified Wood (PNADW)

The DW was soaked in the Nipam/AA mixture solution mentioned above, and the air in the DW was expelled by 30 min of vacuum processing to ensure the mixture solution fully permeates the DW. The wood with the solution is heated at 80 °C for 2.5 h. After Nipam/AA cross‐linking within the wood pores was completed, the wood slices were taken out, immersed in deionized water to remove unreacted monomers and solvents, and then lyophilized to constant weight to obtain PNADW.

### 4.4 Preparation of Hygroscopic Wood (PNADW‐LiCl) and Hygroscopic Aerogel (PNipam/PAA‐LiCl)

The freeze‐dried PNADW and PNipam/PAA were immersed in LiCl solutions of varying concentrations (5, 7.5, 10, 12.5, 15 wt.%) separately for 24 h. Subsequently, the samples were freeze‐dried to yield PNADW‐LiCl and PNipam/PAA‐LiCl aerogels.

### 4.5 Test of Water Absorption and Desorption

The water absorption tests for NW, DW, PNipam/PAA‐LiCl, PNADW, and PNADW‐LiCl were conducted in an LC‐HSP‐70BE constant temperature and humidity chamber. The samples were exposed to 25 °C at different relative humidity (50%, 70%, and 90%) for 12 h, with their weights recorded hourly. Water uptake is calculated using the following Equation (1):

(1) $$\mathrm{Water}\;\mathrm{uptake}\;=\left(m_{1}-m_{0}\right)/m_{0}$$

where m_1_ represents the weight after water absorption (for natural wood, delignified wood, polymer networks, and lignocellulose‐reinforced polymer networks), and m_0_ represents the dry weight of the samples (natural wood, delignified wood, polymer networks, and lignocellulose‐reinforced polymer networks).The desorption experiments of PNADW‐LiCl and DW were performed in the LC‐101‐00B drying oven. Pre‐hydrated samples were desorbed at different temperatures (60 °C, 70 °C, 80 °C, and 90 °C) for 1 h, with their mass recorded at 4‐min intervals. The residual weight is given by Equation (2):

(2) $$\mathrm{Residual}\;\mathrm{weight}\;=\left(m_{a}-m_{0}\right)/m_{b}$$

where m_a_ denotes the mass of the lignocellulose‐reinforced polymer network after a certain desorption time, m_0_ represents the dry weight of the lignocellulose‐reinforced polymer network, and m_b_ is the water content of the sample prior to desorption.

### 4.6 Solar‐Powdered Automatic Atmospheric Water Harvesting Device Design and Outdoor Tests

To achieve round‐the‐clock atmospheric water collection based on modified wood, a solar‐powered, automated, and continuous atmospheric water harvesting device was designed (Figure 5a). The device primarily consists of an absorption–desorption chamber, a control system, and an energy supply unit. The absorption–desorption chamber is fabricated by 3D printing and constructed as a plastic box equipped with a small front door that can be opened on demand. Fin coolers are installed on the sides of the box for efficient vapor condensation, and the PNADW‐LiCl sample is placed on the heating plate in the center of the box (the middle of the box is also configured with two layers of heating plates (Figure S17) based on demand). The control system is employed to regulate the heating and the chamber door. During the sorption, the door remains open with the heating plate deactivated. In contrast, the desorption requires the door to be closed, with the samples heated. The energy supply unit, comprising solar panels, lithium‐ion batteries, and control circuits, powers the entire device. A continuous automatic water collection was tested on the campus of Northeast Forestry University in Harbin, from 8:00 AM on September 25 to 8:00 AM on September 26, 2025, with each cycle of 90 min of sorption and 30 min of desorption. Other continuous automatic water collections were tested in a constant‐temperature, humidity test chamber used to simulate different environments. The atmospheric water collection yield is calculated based on the mass of water collected per cycle, and the ambient temperature and humidity were monitored in real time.

## Funding

S.H. acknowledged the financial support from the China Postdoctoral Science Foundation (2024M760383), Fundamental Research Funds for the Central Universities (2572023CT05‐03), Science and Technology Talents Spring Goose Support Program of Heilongjiang (CYCX24025), and Key R&D Program of Heilongjiang Province (GZ20220134 and JD25A004).

## Conflicts of Interest

The authors declare no conflicts of interest.

## Supporting information




**Supporting File 1**: advs75248‐sup‐0001‐SuppMat.docx

**Supporting File 2**: advs75248‐sup‐0002‐FigureS1‐S20.zip

## Data Availability

The data that support the findings of this study are available from the corresponding author upon reasonable request.
