# Supplementary material for: Autonomous and Continuous Atmospheric Water Harvesting Using Modified Wood
Source: Adv Sci (Weinh). 2026 Apr 13:e75248. Online ahead of print. doi: 10.1002/advs.75248 (PMC13334611; doi:10.1002/advs.75248)
Supplement: Supplementary file 1 — Supporting File 1: advs75248‐sup‐0001‐SuppMat.docx [file ADVS-9999-e75248-s002.docx]

Supporting Information

Autonomous and Continuous Atmospheric Water Harvesting Using Modified Wood

Shiheng He^A,B,C,D^ , Jiaqi Su^A,B,C,D^, Tianting Leng^A,B,C,D^, Yujian Song^A,B,C,D^, Jidong Dong^A,B,C,D^*, Pengfei Huo^A,B,C,D^*, Zhenhua Gao ^A,B,C,D^*, and Shuaiyuan Han^A,B,C,D^*

A. State Key Laboratory of Woody Oil Resources Utilization, Northeast Forestry University, Harbin 150040, China

B. College of Material Science and Engineering, Northeast Forestry University, Harbin 150040, China

C. Engineering Research Center of Advanced Wooden Materials, Ministry of Education, Northeast Forestry University, Harbin 150040, China

D. Key Laboratory of Biobased Material Science and Technology, Ministry of Education, Northeast Forestry University, Harbin 150040, China

*Corresponding Author. Email:

[dongjidong@nefu.edu.cn](mailto:dongjidong@nefu.edu.cn)

[huopengfei@nefu.edu.cn](mailto:huopengfei@nefu.edu.cn)

[gaozh@nefu.edu.cn](mailto:gaozh@nefu.edu.cn)

[hanshuaiyuan@nefu.edu.cn](mailto:Hanshuaiyuan@nefu.edu.cn)

**Table of Contents**

Materials and Characterization S2

Supplementary Figures S3

Supplementary Tables S22

References S24

1. Materials and Characterization

1.1. Materials

Balsa wood slices purchased from Nantong Jimu House E-commerce Co., Ltd. (Jiangsu, China), sourced from Indonesia. N-isopropylacrylamide (Nipam, 98%), acrylic acid (AA, 98%), 2,2'-azobis(2-methylpropionitrile) (AINB, 99%), N, N'-methylene acrylamide (MBA, 99%) and Lithium chloride anhydrous (LiCl, 99.0%) were purchased from Shanghai Titan Technology Co., Ltd. (Shanghai, China). N, N-dimethylformamide (DMF), sodium chlorite (NaClO₂), acetic acid, and anhydrous ethanol were purchased from Tianjin Aopusheng Chemical Co., Ltd. (Tianjin, China). All reagents were used as directly without further purification.

1.2. Characterization

The morphology and microstructure of the samples were observed through scanning electron microscope (SEM, TESCAN MIRA LMS, Czech Republic). The elements distribution in the sample was confirmed by energy-dispersive spectroscopy (EDS, Xplore 30, UK). The molecular structure and composition were characterized using Fourier-transform infrared spectroscopy (FT-IR, Nicolet iN10, Thermo Fisher Scientific Inc, USA) in the wavenumber range of 4000–500 cm⁻¹, and by laser-scanning confocal Raman spectroscopy (Ram, Onvia, UK) in the wavenumber range of 600–2000 cm⁻¹. The crystallinity of the samples was tested using an X-ray diffraction (XRD, Smart Lab 9KW, Japan) within the 5–70° (2θ) angle range. The elemental composition and bonding patterns of the samples were determined using X-ray photoelectron spectroscopy (XPS, Thermo Scientific K-Alpha, USA). The thermal decomposition process of the samples was investigated using a thermogravimetric analyzer (TGA, Rigaku 8122, Japan) under 10 °C /min from 35 °C to 600 °C in a nitrogen atmosphere. The LCST of the samples was determined using differential scanning calorimetry (DSC, Q20, TA Instruments, USA) with 1 °C / min from 20 °C to 40 °C. The purity of water collected from the atmosphere was tested using inductively coupled plasma optical emission spectroscopy (ICP-OES/MS, Agilent 5110 (OES), USA). Contact angles of the samples were observed using a video optical contact angle measuring instrument (Theta Flex, Germany). Mechanical properties of the samples were tested using the UTM2203 electronic universal testing machine (Shenzhen Suns Technology Stock Co., Ltd.)

2. Supplementary Figures


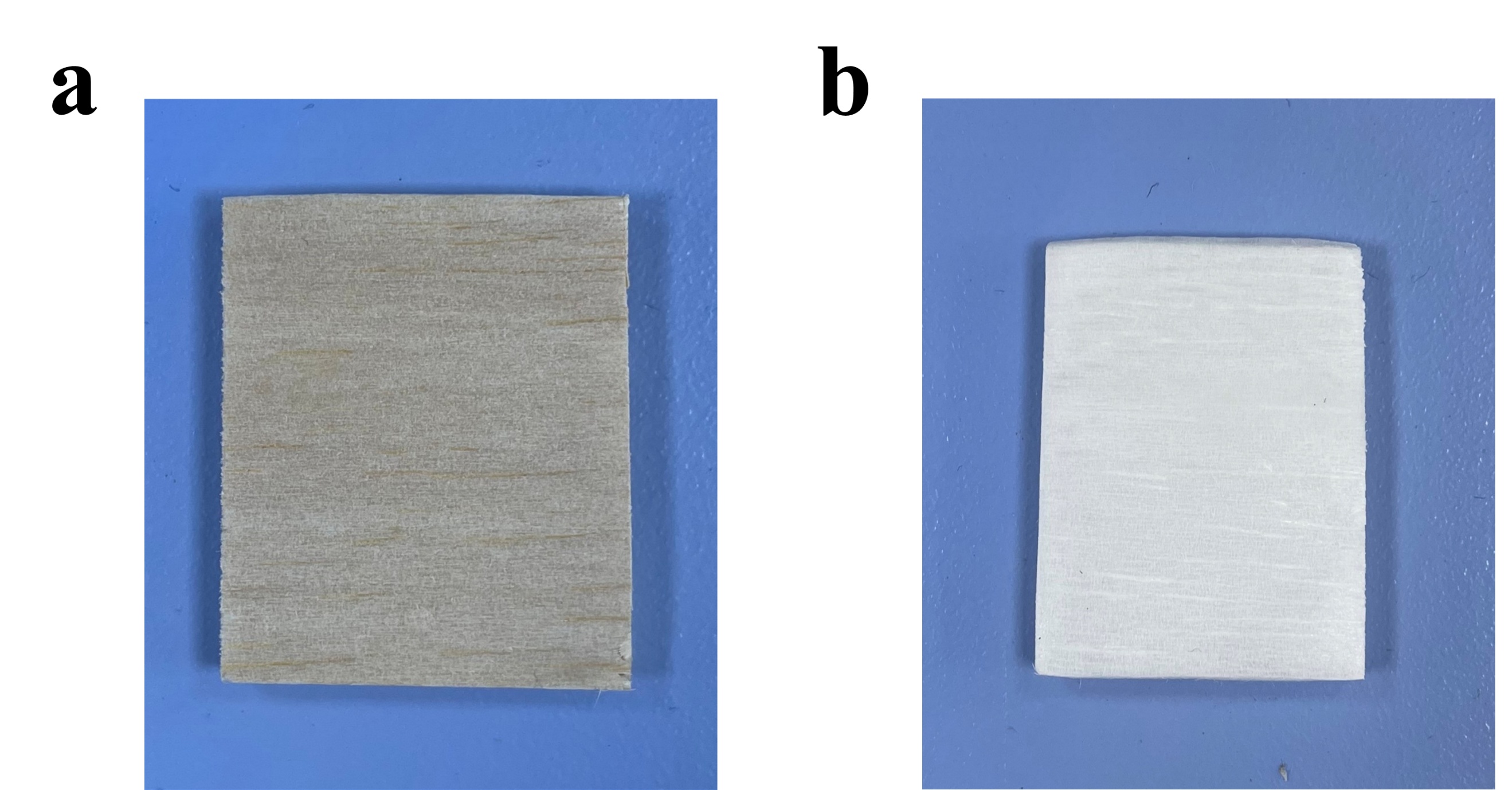


**Figure S1.** a) Optical image of natural wood (NW); b) Optical image of delignified wood (DW).


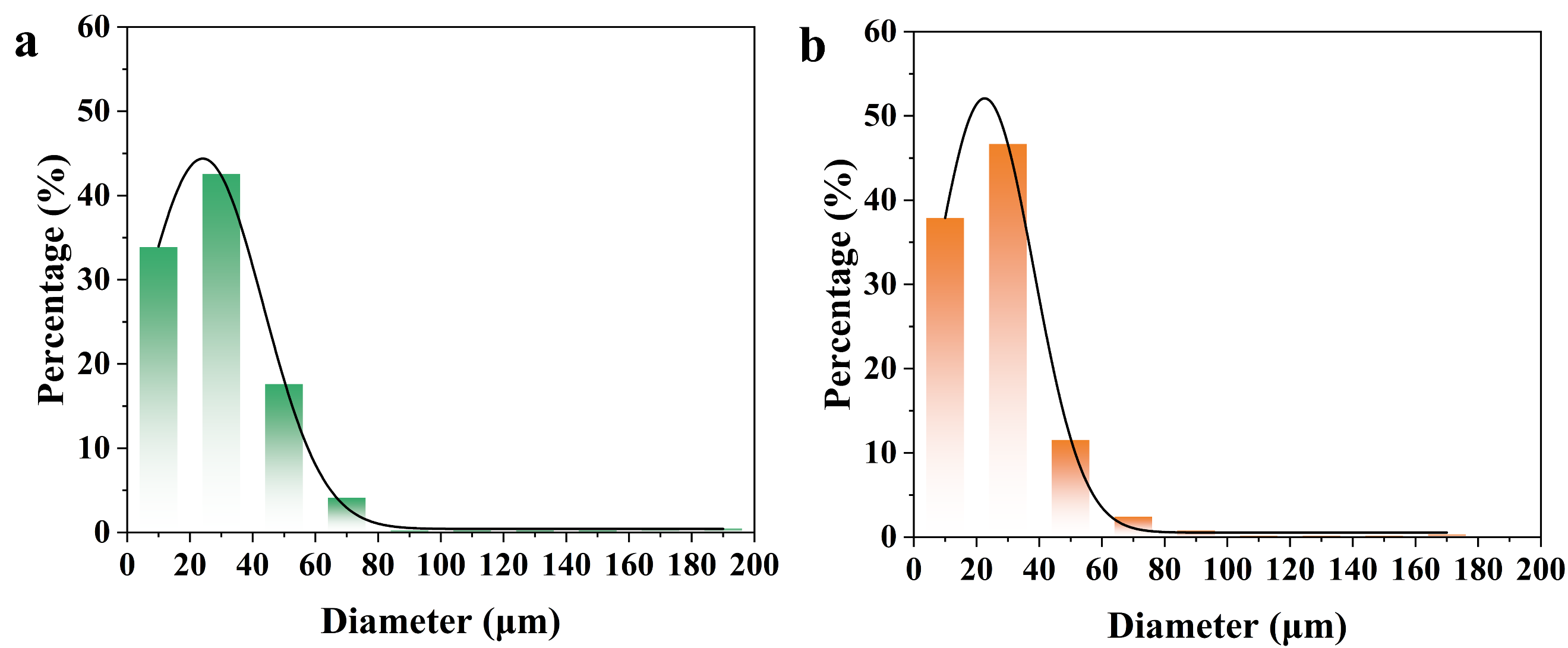


**Figure S2.** Pore size distribution of a) PNADW and b) PNADW-LiCl measured by Nano Measurer.


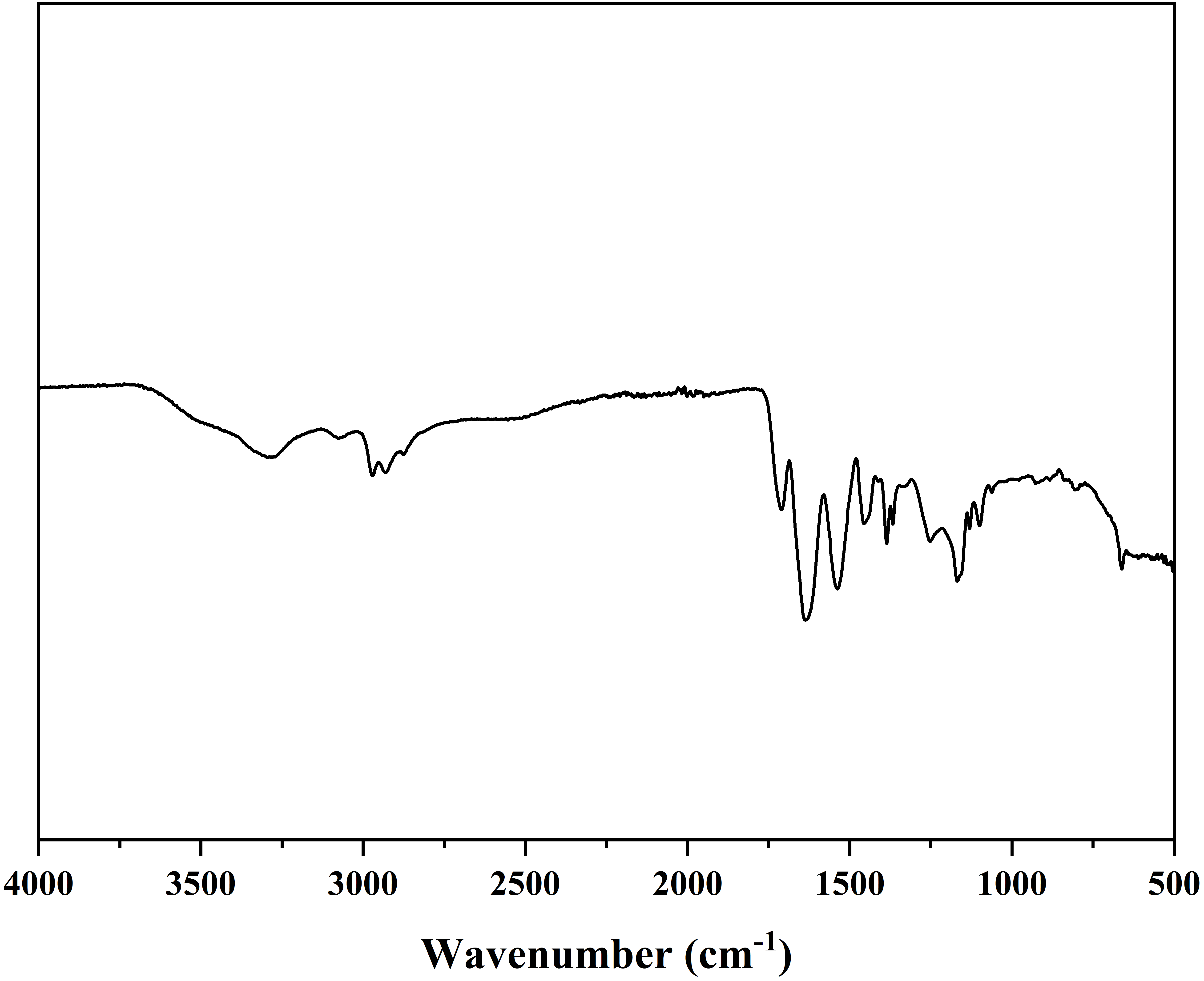


**Figure S3.** FT-IR spectrum of PNipam/PAA.


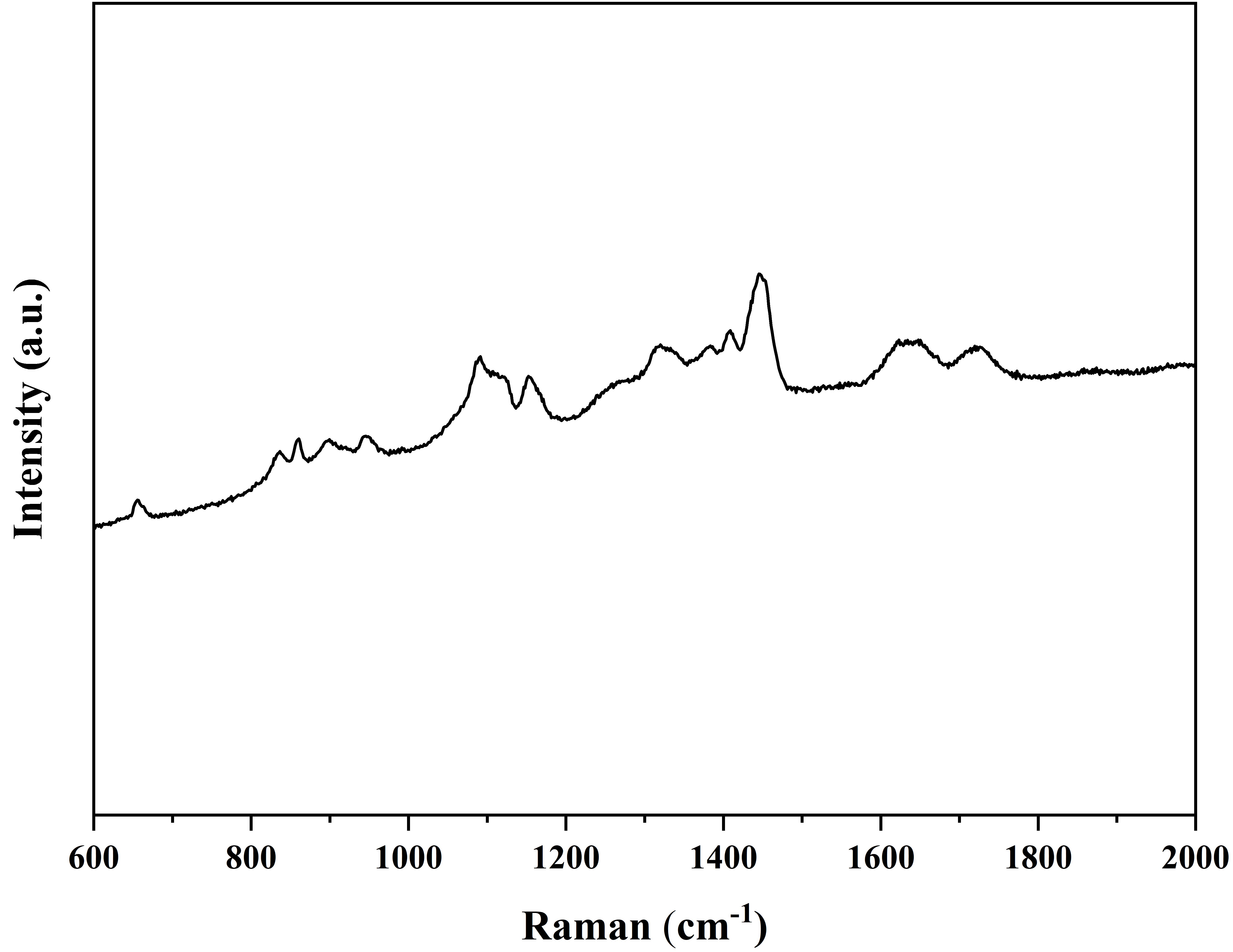


**Figure S4.** Raman spectrum of PNipam/PAA.


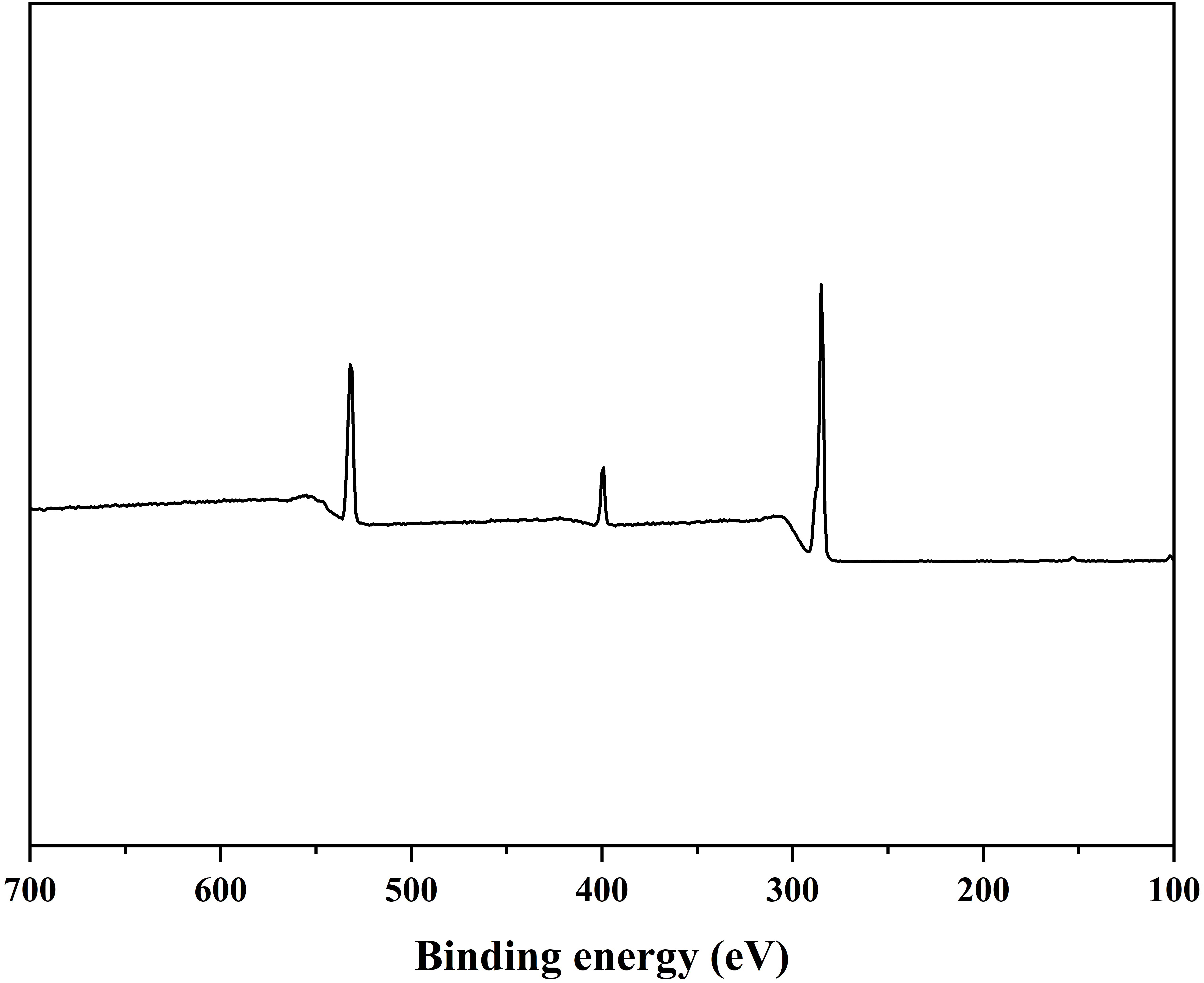


**Figure S5.** XPS spectrum of PNipam/PAA.


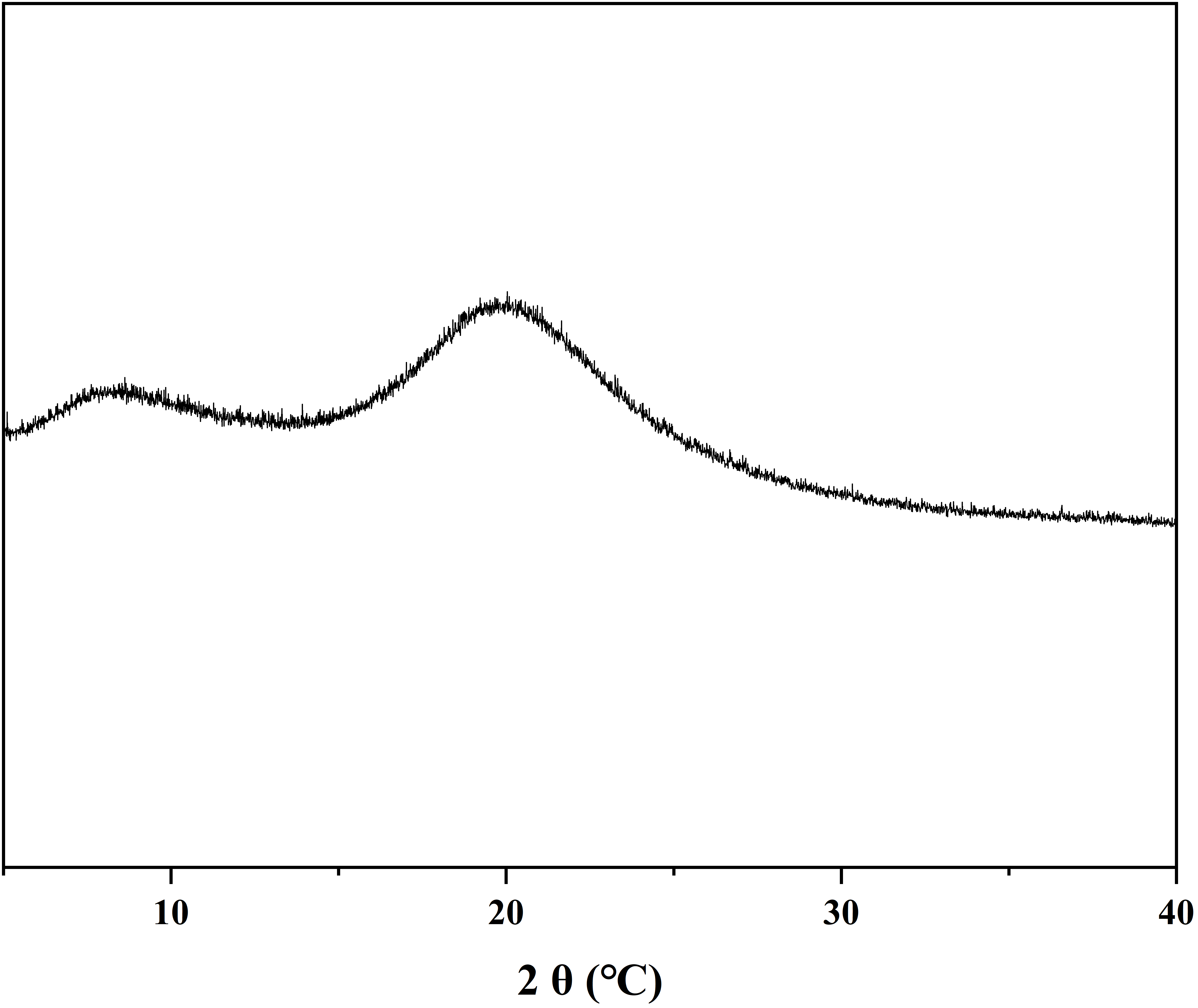


**Figure S6.** XRD pattern of PNipam/PAA.


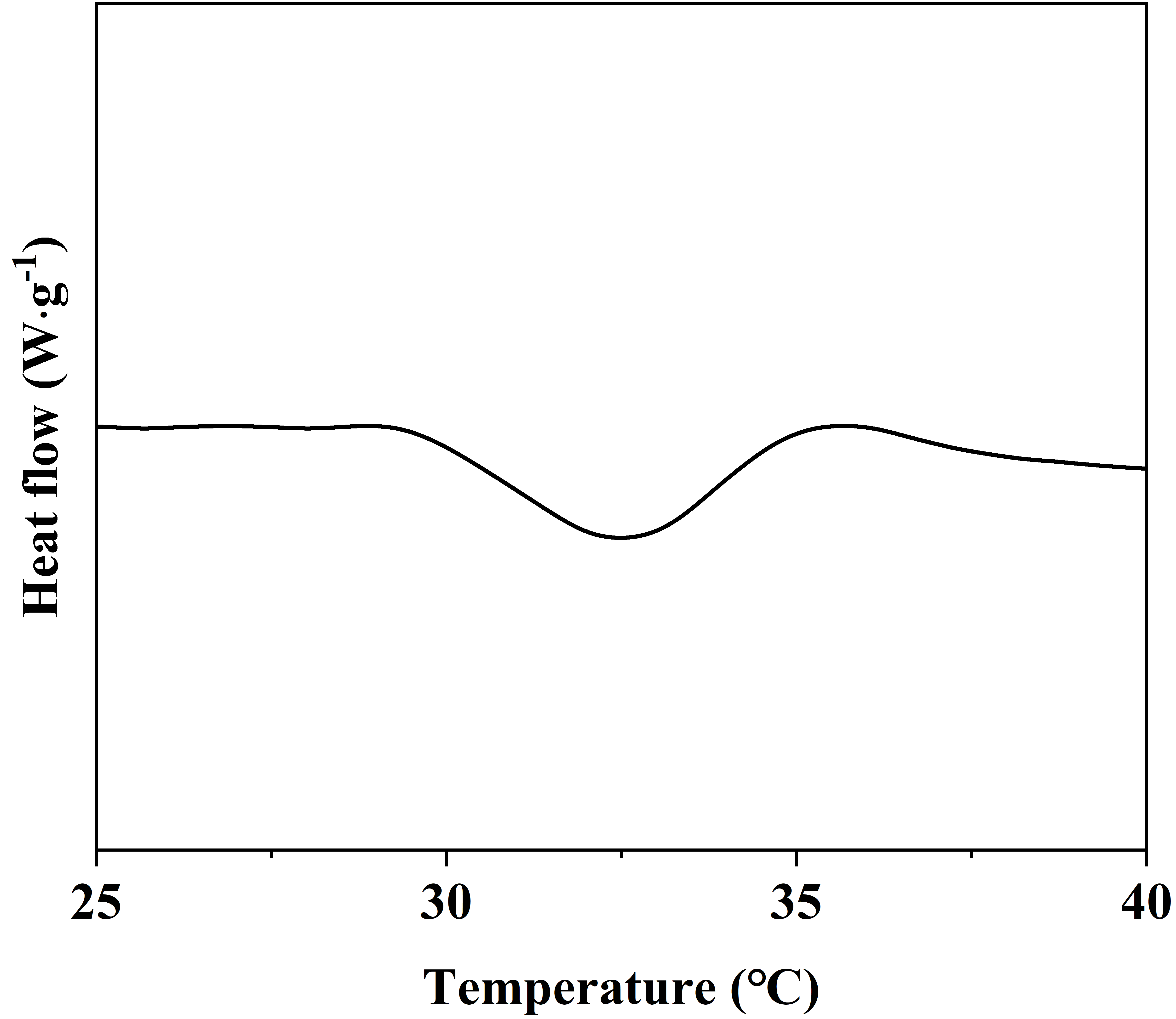


**Figure S7.** Retest the PNADW/LiCl samples for LCST


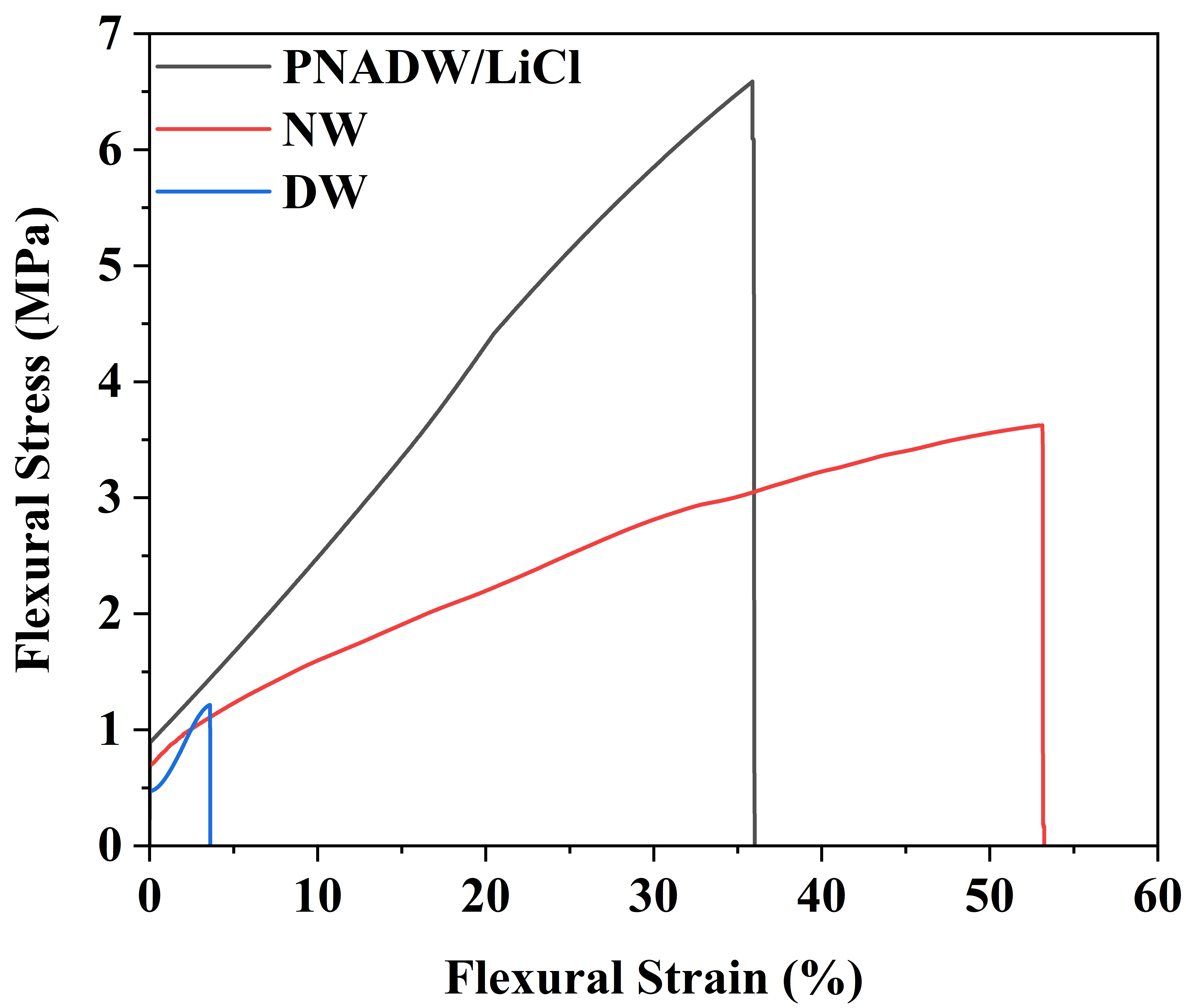


**Figure S8.** Bending stress-strain curves of NW, DW and PNADW-LiCl.


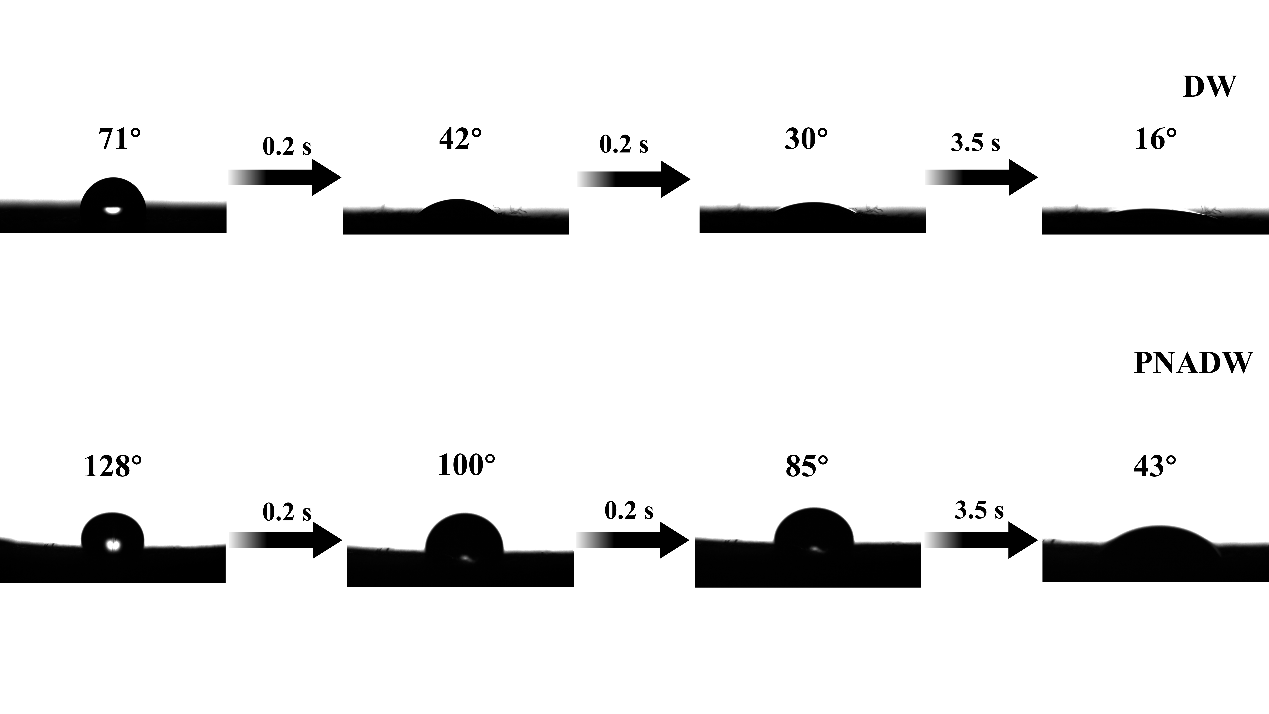


**Figure S9.** Dynamic contact angle measurements for DW and PNADW.


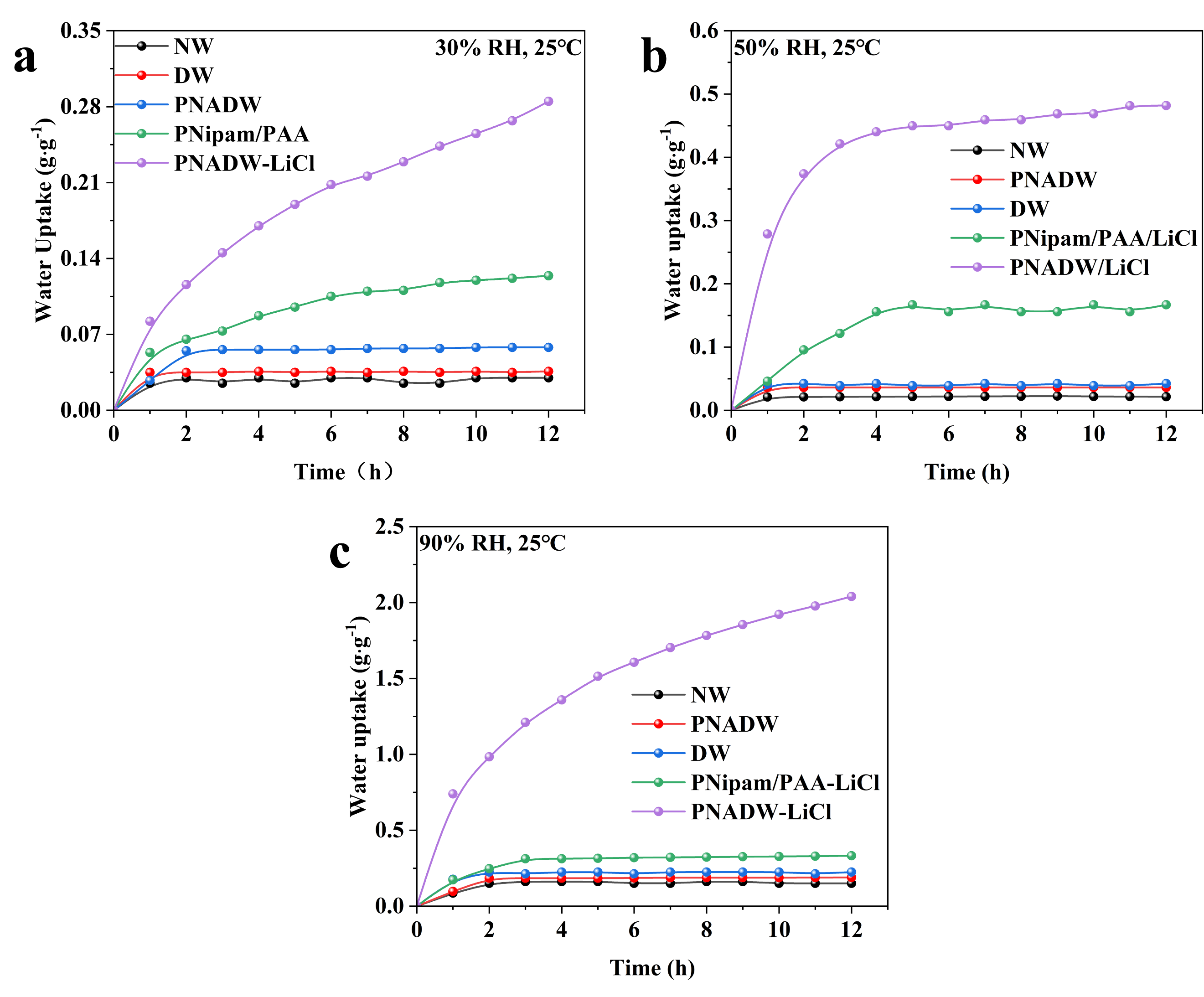


**Figure S10.** a) Water uptake of PNADW-LiCl and other samples at 30% RH; b) Water uptake of PNADW-LiCl and other samples at 50% RH; c) Water uptake of PNADW-LiCl and other samples at 90% RH.


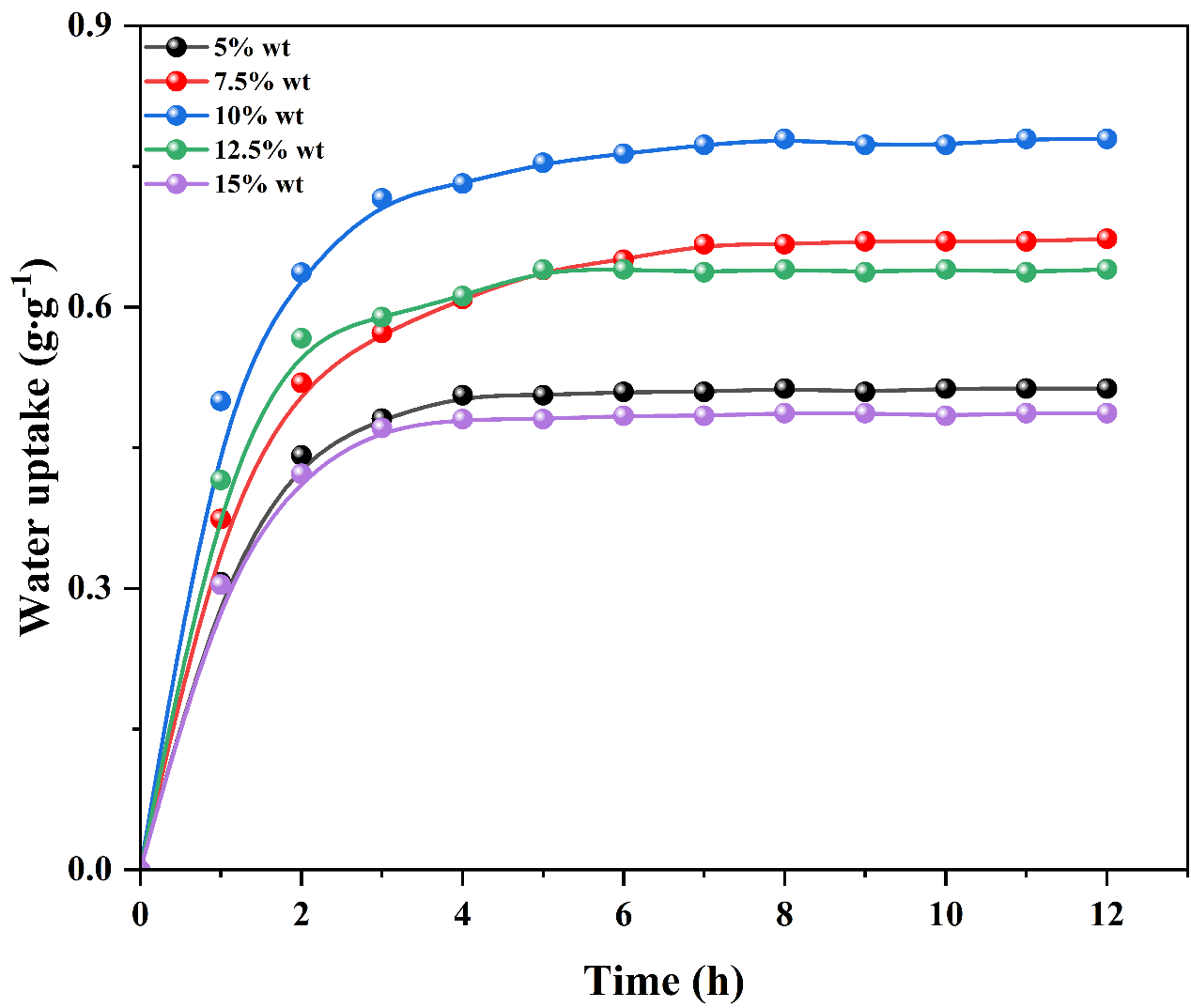


**Figure S11.** Water uptake of PNADW-LiCl immersed in LiCl solutions of varying mass concentrations.


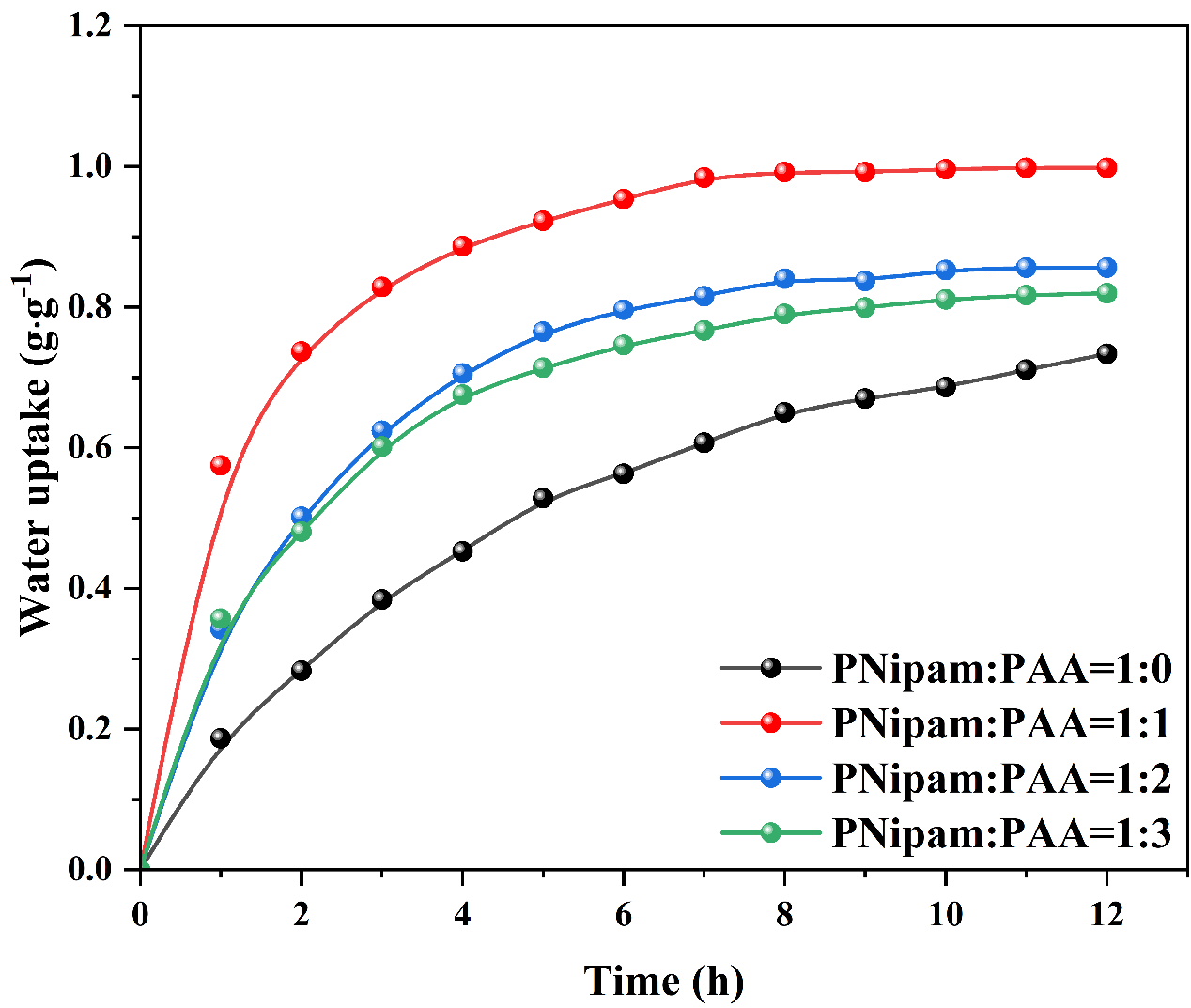


**Figure S12.** Water uptake of PNADW-LiCl as a function of the molar ratio of PNipam to PAA for different amounts of each substance.


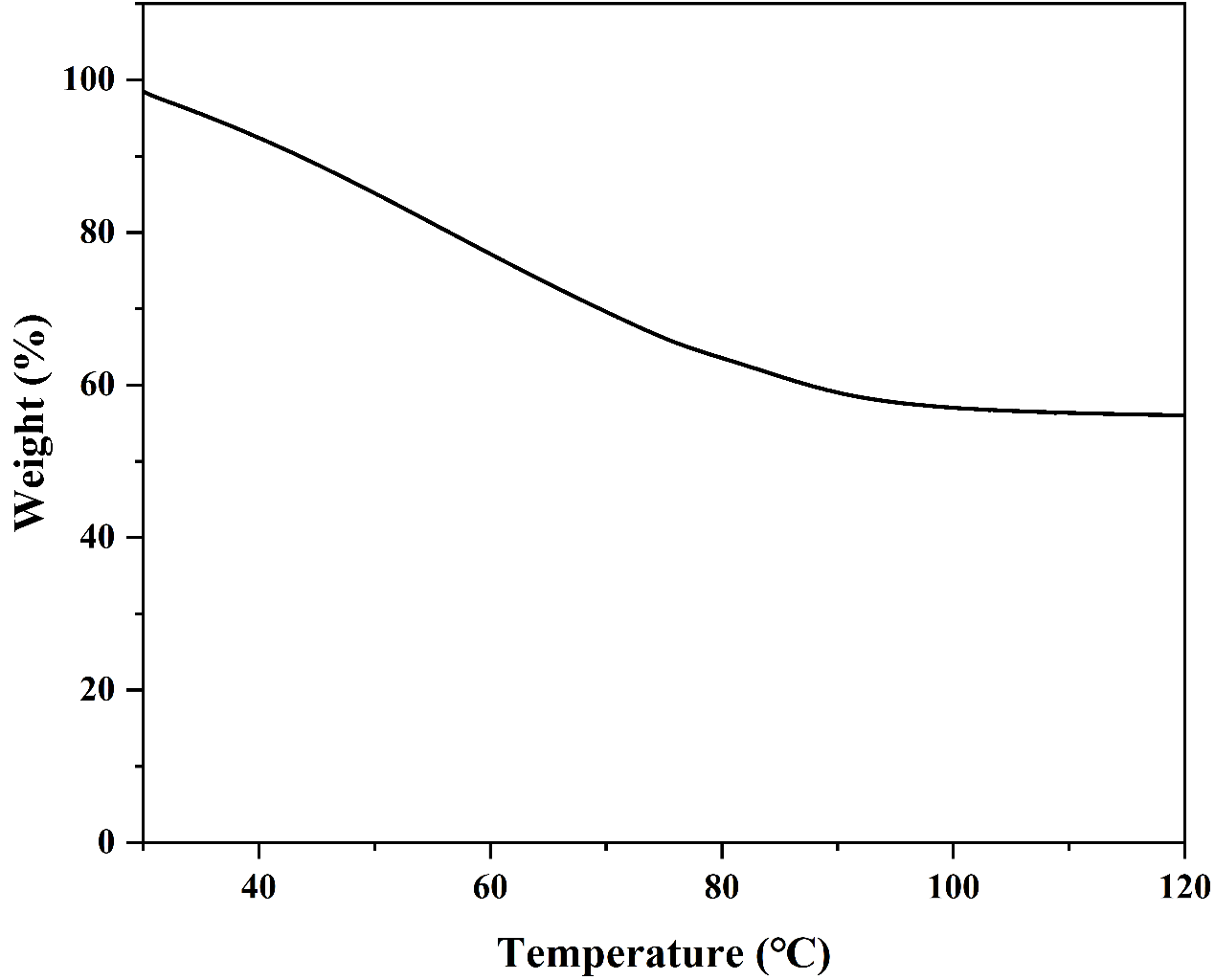


**Figure S13.** TGA weight loss curve of PNADW-LiCl sample adsorbed at 70% relative humidity for 1 hour.


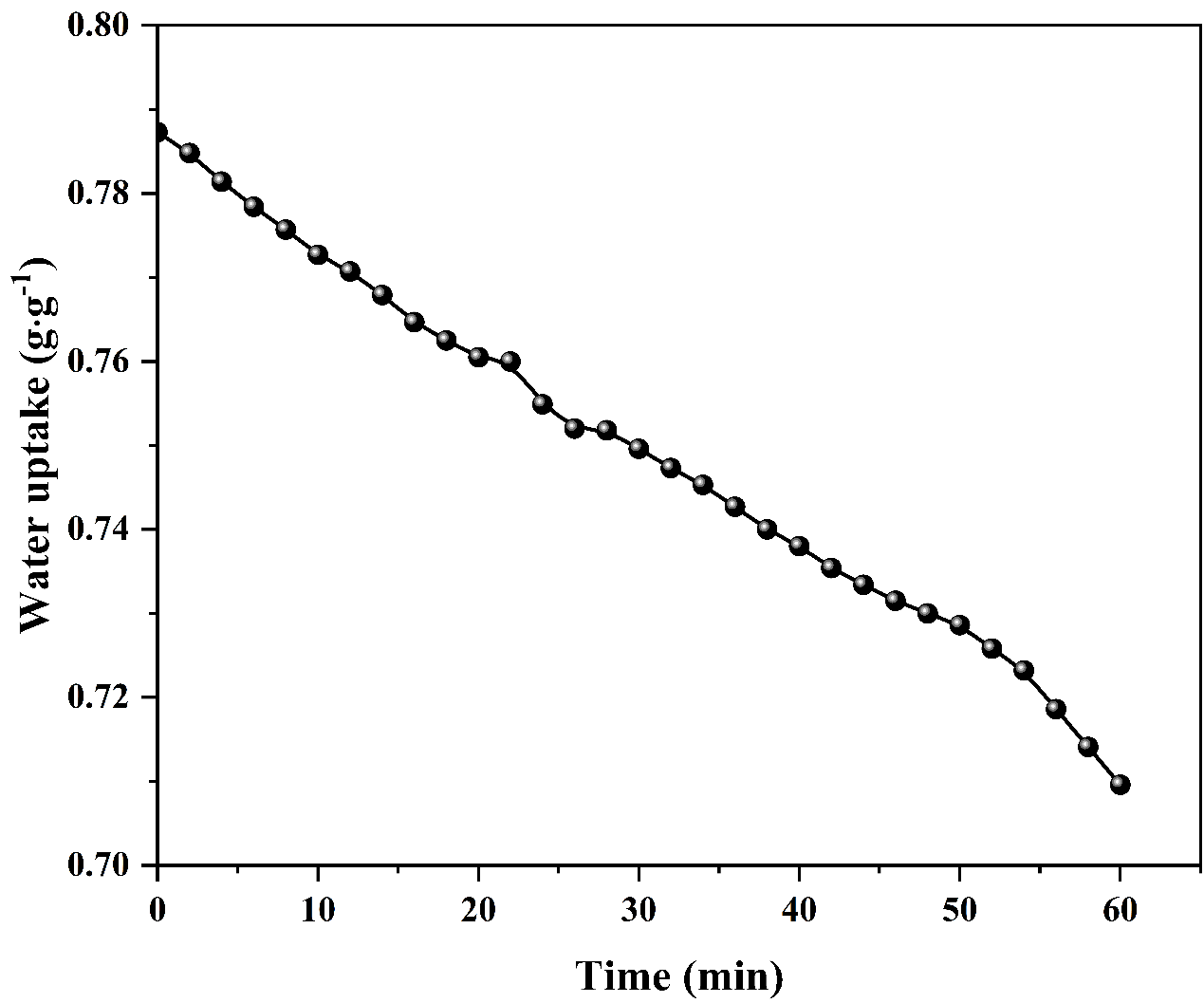


**Figure S14.** Desorption experiment of PNADW-LiCl at 40 °C.


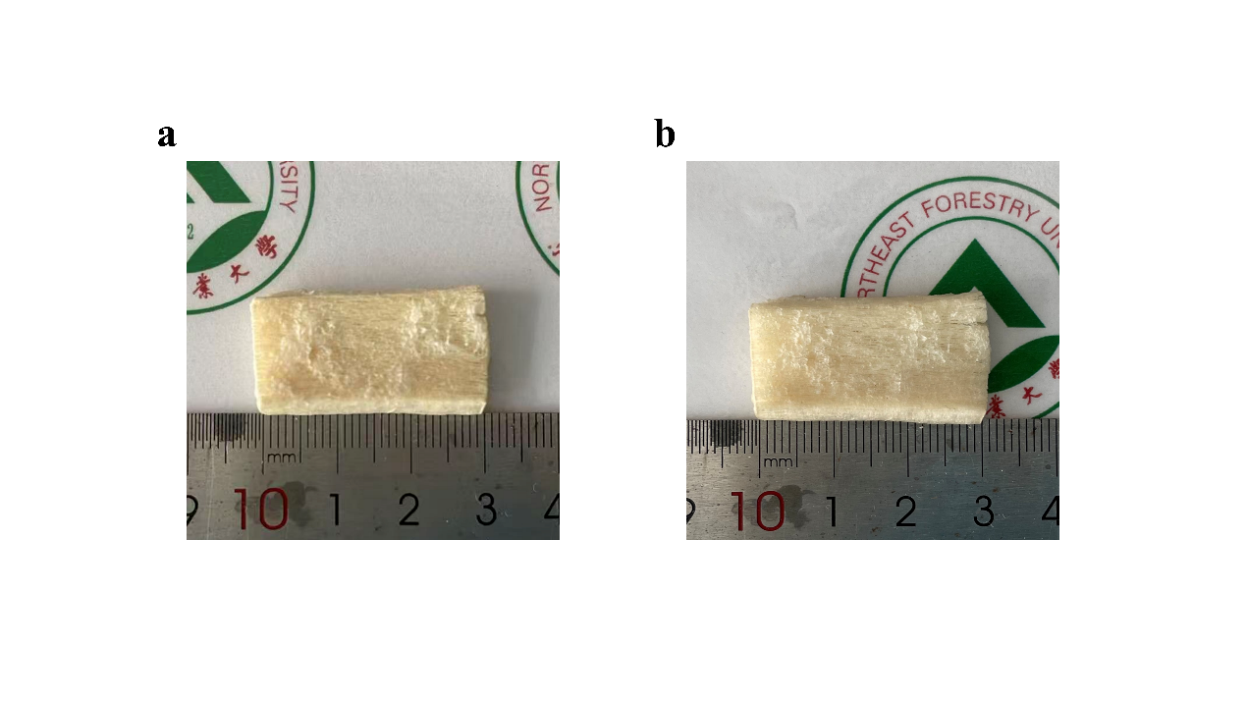


**Figure S15.** Digital photos of a) original PNADW-LiCl; b) PNADW-LiCl after 10 cycles.


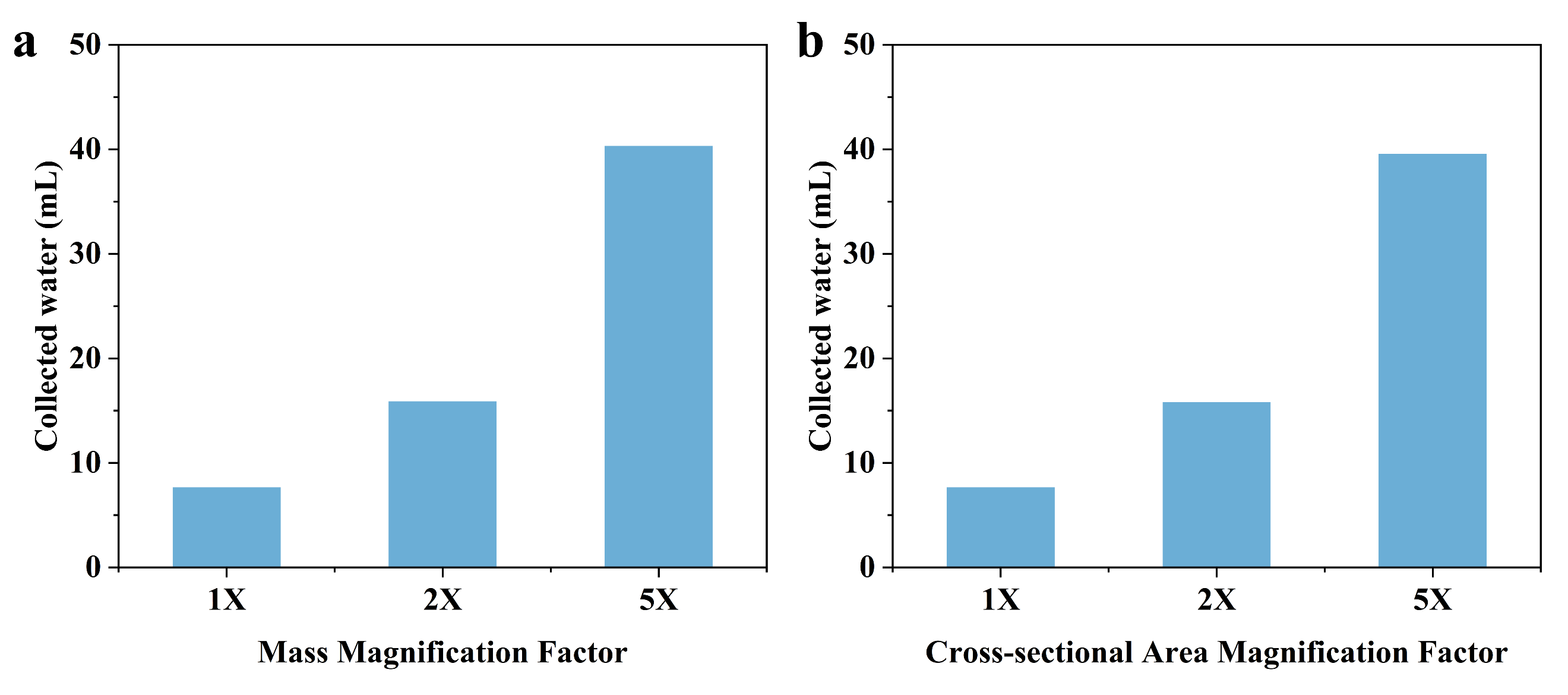


**Figure S16.** (a) Atmospheric water collection with different mass magnification and (b) different cross-sectional amplification magnification


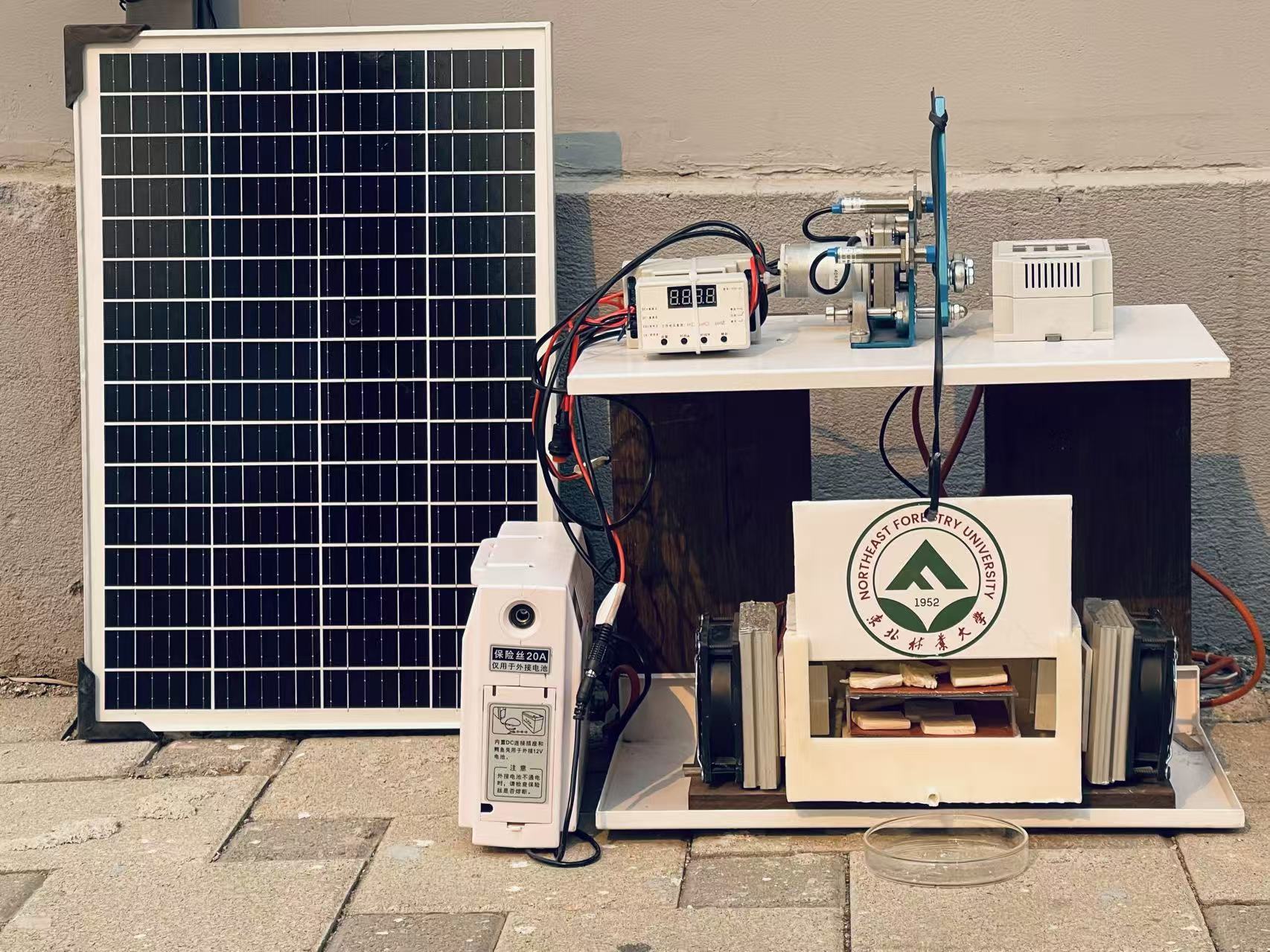


**Figure S17.** Expandable absorption-desorption chamber (double heating plate) for atmospheric water collection systems


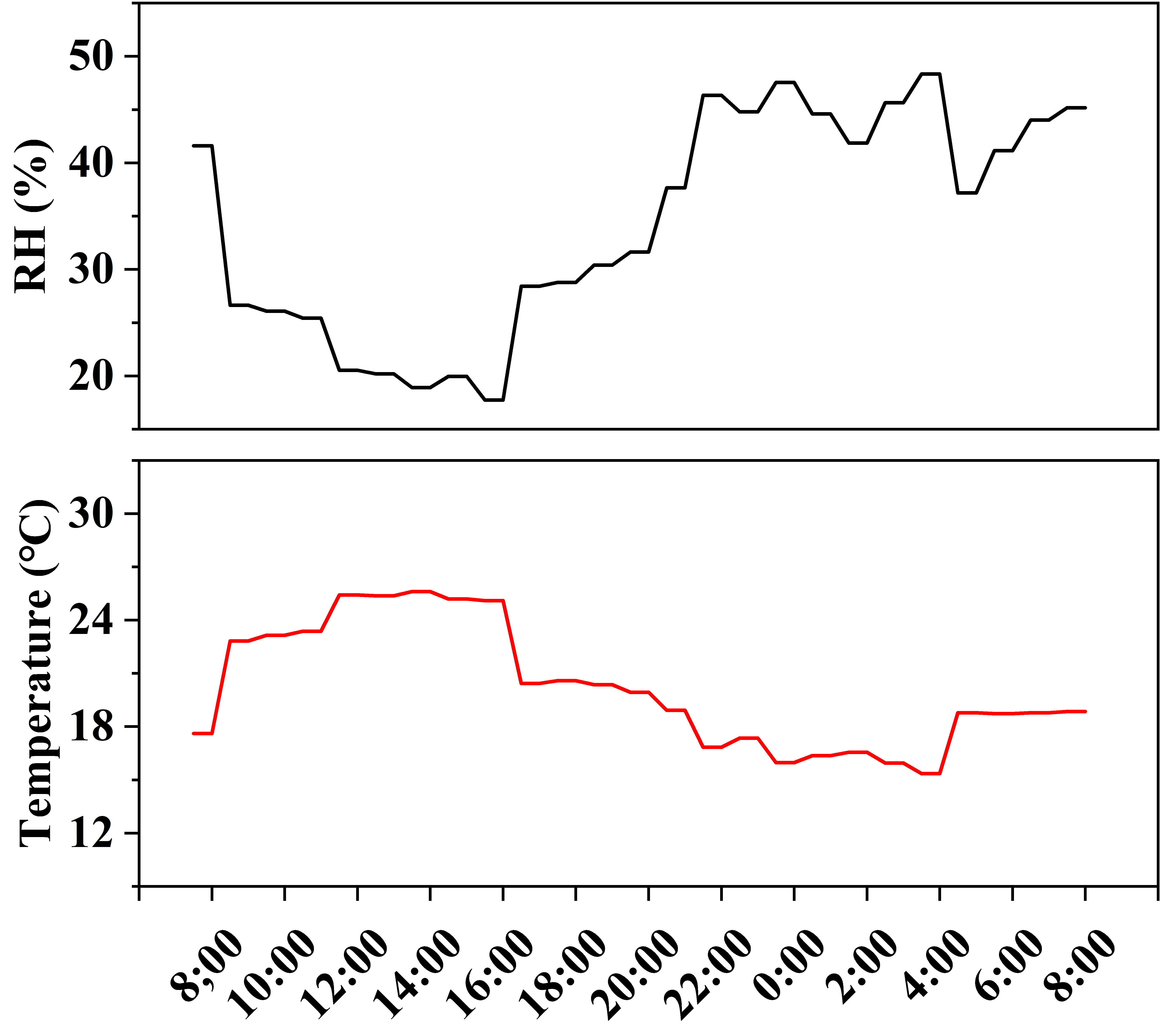


**Figure S18.** Simulated temperature and humidity for the driest day in Harbin in 2025


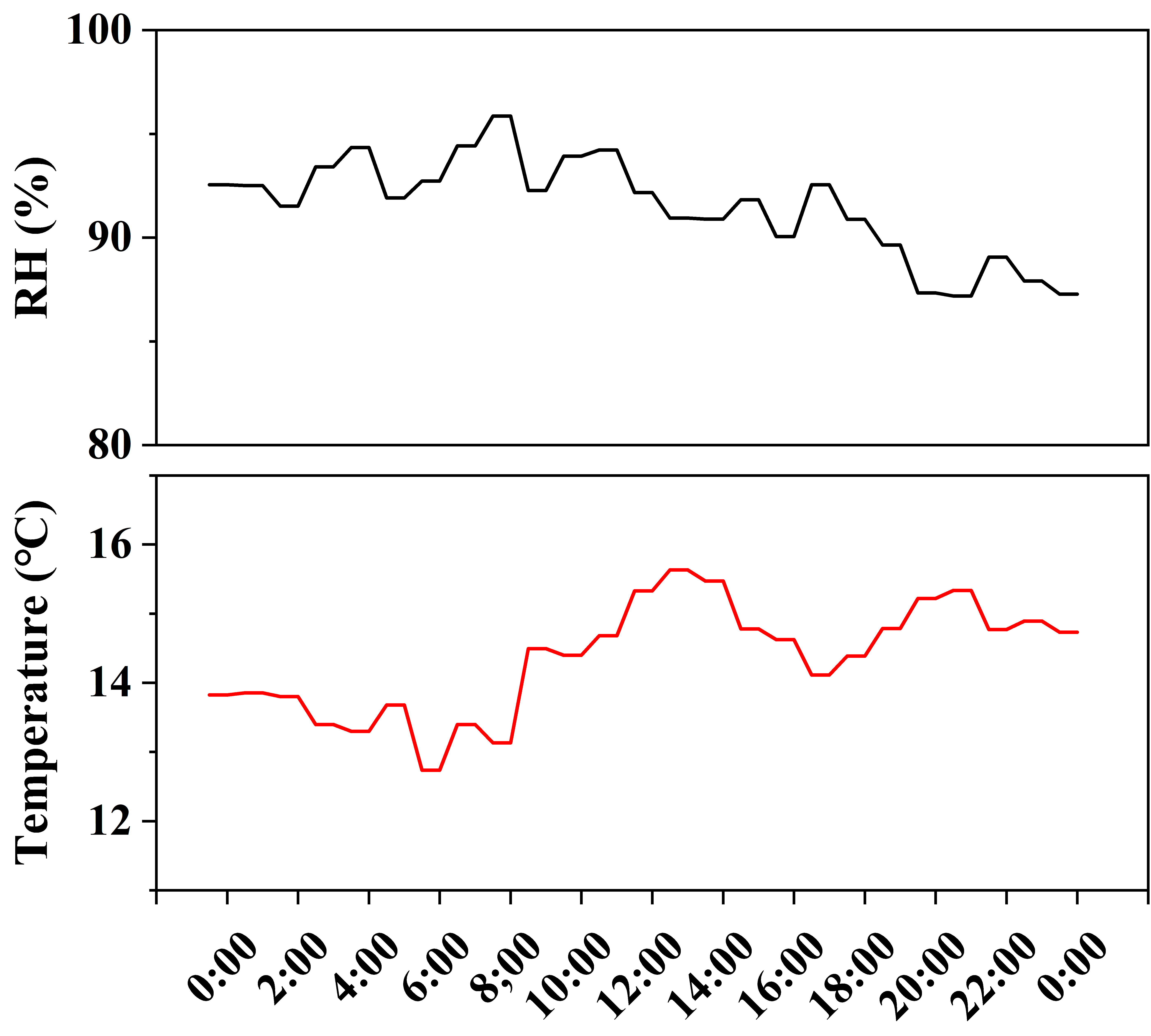


**Figure S19.** Simulated temperature and humidity for the most humid day in Harbin in 2025


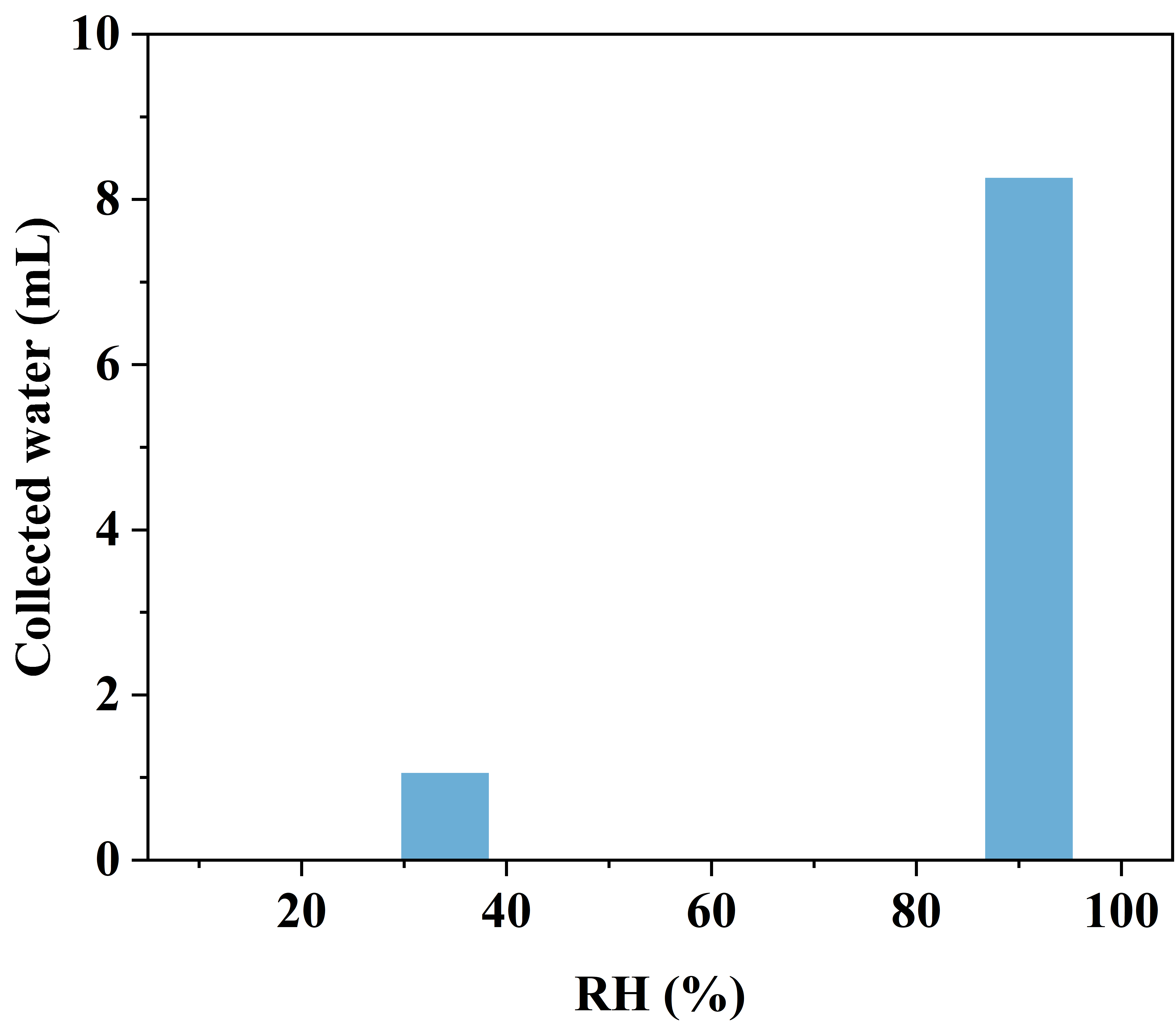


**Figure S20.** Comparison of water collection performance under two weather conditions.

3. Supplementary Table

**Table S1.** The atomic percentages of PNADW-LiCl.

| Sample | Chemical composition (At %) | | | |
| --- | --- | --- | --- | --- |
|  | C | O | N | Cl |
| NW | 70.32 | 29.68 | 0 | 0 |
| DW | 68.88 | 31.08 | 0 | 0 |
| PNADW | 69.46 | 23.64 | 6.9 | 0 |
| PNADW-LiCl | 60.84 | 22.47 | 4.38 | 12.3 |

**Table S2.** Summary of AWH materials reported in the literature.

| AWH | Water uptake (g·g^-1^) | RH (%) |
| --- | --- | --- |
| SA/CNT/MC^[1]^ | 0.77/2.03/3.24 | 60/80/90 |
| PPy-COF/LiCl^[2]^ | 0.18/1.02/2.65 | 50/70/90 |
| G-PDDA^[3]^ | 0.13/0.37/1.1 | 30/60/90 |
| ILCA^[4]^ | 0.5/1.2/1.5 | 30/60/90 |
| MOF-801^[5]^ | 0.19/0.23/0.25/0.29 | 30/50/60//90 |
| MOF-801@P(NIPAM-GMA)^[5]^ | 0.33/0.36/0.44/0.54 | 20/30/60/90 |
| PVF-Ppy-MnC^[6]^ | 0.43/0.86/2.12 | 50/70/90 |
| BHA-213^[7]^ | 0.52/0.78/1.11/3.18 | 30/40/60/80 |
| MTC^[8]^ | 0.395/0.61 | 60/80 |
| HOC270^[9]^ | 0.31/0.43/0.52/0.58 | 20/30/40/50 |
| BCS^[10]^ | 0.15/0.25 | 20/40 |
| DWM-PM^[11]^ | 0.35/0.60/0.82 | 30/60/80 |

**Table S3.** The moisture absorption capacity of biomass adsorbents over the past five years.

| AWH | Water uptake (g·g^-1^) | RH (%) |
| --- | --- | --- |
| SA/CNT/MC^[1]^ | 0.87 | 70 |
| LiCl@CGA-15%^[12]^ | 1.2 | 60 |
| cl-SP^[13]^ | 2.90 | 60 |
| CPPY@LiCl^[14]^ | 2.65 | 60 |
| LBC@LiCl^[15]^ | 1.12 | 60 |
| Alg-Ca/PDA^[16]^ | 0.33 | 80 |
| CA^[17]^ | 0.12 | 60 |
| CS/SA@MF^[18]^ | 1.00 | 70 |
| LiCl@GG-CNF/CNT^[19]^ | 0.9 | 60 |
| SCNCPA-EF^[20]^ | 0.95 | 60 |
| FD^[21]^ | 0.53 | 50 |
| SA/CCS/C/CaCl_2_^[22]^ | 0.9 | 60 |
| CF-G-LiCl^[23]^ | 0.367 | 63 |
| LiCl@GQC4^[24]^ | 0.83 | 65 |
| CFA^[25]^ | 2.3 | 60 |
| SBAC/CaCl_2_^[26]^ | 1.2 | 60 |
| ZHPC−3/LiCl^[27]^ | 1.87 | 60 |
| PNIPAM/TO-CNF/LiCl^[28]^ | 1.79 | 60 |
| SMCA^[29]^ | 0.97 | 70 |
| SCG-5^[30]^ | 0.84 | 70 |
| RKL-C@LiCl-8^[31]^ | 1.82 | 60 |
| DBJA-75^[32]^ | 0.9 | 75 |
| NBHA^[33]^ | 1.0 | 65 |
| BHA-210^[34]^ | 0.94 | 80 |
| SA_0.15_PAM_1.5_^[35]^ | 0.8 | 70 |
| CNF/SA fibers^[36]^ | 0.7 | 75 |

4. References

1 X.P. Zhang, H. Qu, X.Y. Li, L.A. Zhang, Y.X. Zhang, J.C. Yang, M.J. Zhou, L. Suresh, S.Q. Liu, S.C. Tan, Adv. Mater. **2024**, 36, 2310219.

2 M. Xia, D. Cai, J.B. Feng, P. Zhao, J.K. Li, R.X. Lv, G.Q. Li, L.L. Yan, W. Huang, Y.P. Li, Z.Y. Sui, M. Li, H. Wu, Y.J. Shen, J.X. Xiao, D. Wang, Q. Chen, Adv. Funct. Mater. **2023**, 33,.

3 K.J. Yang, T.T. Pan, I. Pinnau, Z. Shi, Y. Han, Nano Energy **2020**, 78, 105326

4 F.F. Deng, C.X. Wang, C.J. Xiang, R.Z. Wang, Nano Energy **2021**, 90, 106642.

5 C.R. Yang, H. Wu, J. Yun, J.S. Jin, H. Meng, J. Caro, J.G. Mi, Adv. Mater. **2023,** 35, 2210235.

6 H. Shan, P. Poredos, H. Qu, X. Yang, M. Zhou, L. Bai, J. Shi, W. Chen, R. Wang, S. Tan, Adv. Funct. Mater. **2024**, 34, 2402839.

7 L. Zhang, T. Yang, Y. Niu, X. Xu, M. Helal, M. Hessien, Y. Qiu, Y. Zhong, L. Shen, X. Ji, M. An, Z. El-Bahy, J. Zhang, Y. Zhou, Z. Li, Adv. Mater. **2026**, 38, e22241.

8 Q. Luo, M. Chen, D. Yu, T. Zhang, J. Zhao, L. Zhang, X. Han, M. Zhou, Y. Hou, Y. Zheng, ACS Nano **2024**, 18, 14650.

9 B. Fu, J. Zhang, N. Robinson, Z. Zhang, Z. Zhu, M. Dong, X. Zhang, J. Kang, P. Michalski, Z. Zhao, J. Ji, Y. Xu, K. Zhang, X. Wang, S. Chen, H. Xu, P. Liu, H. Yin, H. Zhao, Adv. Mater. **2025**, 37, e11336.

10 Y. Song, N. Xu, G. Liu, H. Qi, W. Zhao, B. Zhu, L. Zhou, J. Zhu, Nat. Nanotechnol. **2022**, 17, 857.

11 J. Liang, D. Fan, C. Cai, Z. Su, Y. Lu, Adv. Funct. Mater. **2026**, 36, e18960.

12 M. Song, F. Shao, L. Wang, H. Xie, W. Yu, Sol. Energy Mater. Sol. **2023**, 262, 112532.

13 X. Chang, S. Li, N. Li, S. Wang, J. Li, C. Guo, L. Yu, P. Murto, X. Xu, J. Mater. Chem. A **2022**, 10, 18170.

14 B.W. Lin, W.J. Ying, C.F. Li, J.Z. Liu, L.K. Zhou, H. Zhang, R.Z. Wang, J.Y. Wang, Adv. Funct. Mater. **2025**, 35, 2500679.

15 W. Yao, X. Zhu, Z. Xu, R. Davis, G. Liu, H. Zhong, X. Lin, P. Dong, M. Ye, J. Shen, ACS Appl. Mater. Interfaces **2022**, 14, 4680.

16 M. Wu, Y. Zhou, S. Aleid, X. Tang, Y. Zhao, R. Li, P. Wang, ACS Sustain. Chem. Eng. **2024**, 12, 1255.

17 N. Oumous, A. Koukouch, M. Asbik, J. Environ. Chem. Eng. **2025**, 13, 120000.

18 Z. Duan, P. Feng, M. Ding, C. Liu, C. Li, J. Zhang, Adv. Funct. Mater. **2026**, 36, e11143.

19 J. Li, G. Xing, M. Qiao, Z. Liu, H. Sun, R. Jiao, L. Li, J. Zhang, A. Li, Langmuir **2023**, 39, 18161.

20 Y. Lu, Z. Li, Y. Xie, Y. Liu, J. Zhang, L. Zong, ACS Mater. Lett. **2024**, 6, 976.

21 V. Gentile, M. Calò, M. Bozlar, M. Simonetti, F. Meggers, Int. J. Heat Mass Transf. **2024**, 219, 124794.

22 C.H. Fu, D.Y. Zhan, G.Y. Tian, A.H. Yu, L. Yao, Z.G. Guo, ACS Appl. Mater. Interfaces **2024**, 16, 35740.

23 H. Xue, B. Chen, Y. Wang, Langmuir **2024**, 40, 12810.

24 Z. Chen, C. Zhao, Y. Wang, C. Tu, Y. Guo, C. Yan, M. Yang, Carbohydr. Polym. **2026**, 377, 124874.

25 H. Zhang, Z. Zhou, J. Du, X. Pei, L. Zhou, J. Clean Prod. **2023**, 416, 137897.

26 G. Raveesh, R. Goyal, S.K. Tyagi, Sep. Purif. Technol. **2025**, 356, 129820.

27 W.X. Guan, Y.X. Zhao, C.X. Lei, Y.Y. Wang, K. Wu, G.H. Yu, Adv. Mater. **2025**, 37, 2420319.

28 X. Wang, H. Zhang, X. Liu, J. Du, Y. Xu, Polymers **2025**, 17, 17162253.

29 J. Sun, B. An, K. Zhang, M. Xu, Z. Wu, C. Ma, W. Li, S. Liu, J. Mater. Chem. A **2021**, 9, 24650.

30 N. Nguyen, P. Phan, V. Truong, T. Le, D. Hoang, Langmuir 2025, 41, 33776.

31 J. Yan, J. Lu, T. Bai, L. Li, Z. Niu, Y. Yue, W. Cheng, G. Han, O. Rojas, Adv. Funct. Mater. **2025**, e24104.

32 Y. Liu, R. Feng, Y. Zhao, X. Guo, J. Ding, S. Liu, Y. Wang, J. Zhu, X. Li, Adv. Mater. **2025**, 37, e12244.

33 M.Z. Wang, T.M. Sun, D.H. Wan, M. Dai, S.J. Ling, J.L. Wang, Y.Q. Liu, Y. Fang, S.H. Xu, J.J. Yeo, H.P. Yu, S.X. Liu, Q.W. Wang, J. Li, Y. Yang, Z.J. Fan, W.S. Chen, Nano Energy **2021**, 80, 105569.

34 L. Zhang, T. Yang, Y. Niu, X. Xu, M. Helal, M. Hessien, Y. Qiu, Y. Zhong, L. Shen, X. Ji, M. An, Z. El-Bahy, J. Zhang, Y. Zhou, Z. Li, Adv. Mater. **2026**, 38, e22241.

35 X. Li, X. Mu, F. He, Y. Chen, Y. Wang, Z. Zhang, X. Wang, J. Zhou, J. Liu, L. Miao, ACS Appl. Eng. Mater. **2025**, 3, 1051.

36 A. Zheng, J. Pan, D. Nian, S. Lu, Y. Cheng, Z. Du, Chem. Eng. J. **2026**, 529, 173152.
