# Supplementary figures and images for: Autonomous and Continuous Atmospheric Water Harvesting Using Modified Wood
Source: Adv Sci (Weinh). 2026 Apr 13:e75248. Online ahead of print. doi: 10.1002/advs.75248 (PMC13334611; doi:10.1002/advs.75248)

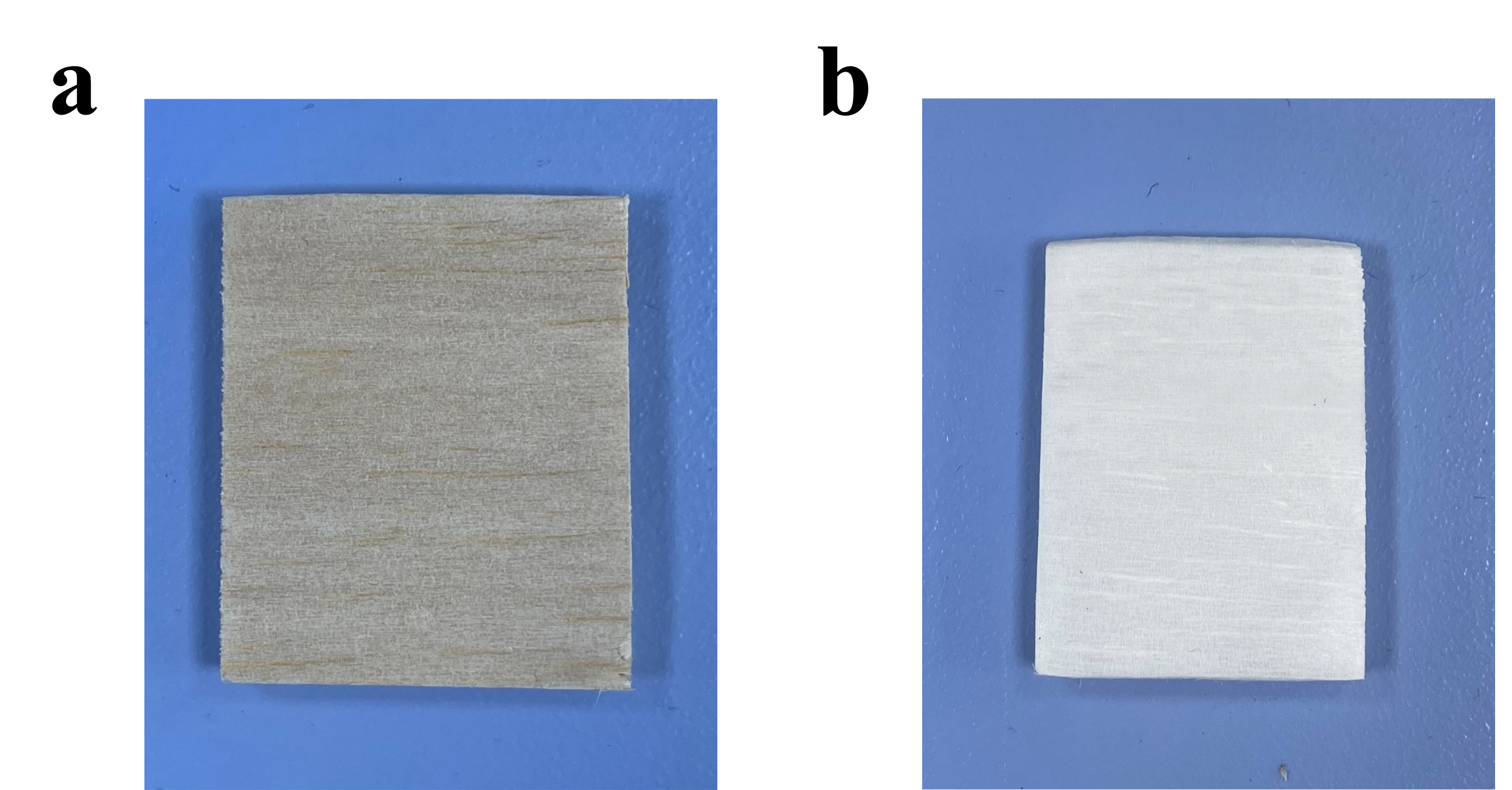

Supplement: Supplementary file 2 — Supporting File 2: advs75248‐sup‐0002‐FigureS1‐S20.zip [file ADVS-9999-e75248-s001.zip › Figure S1.tif]

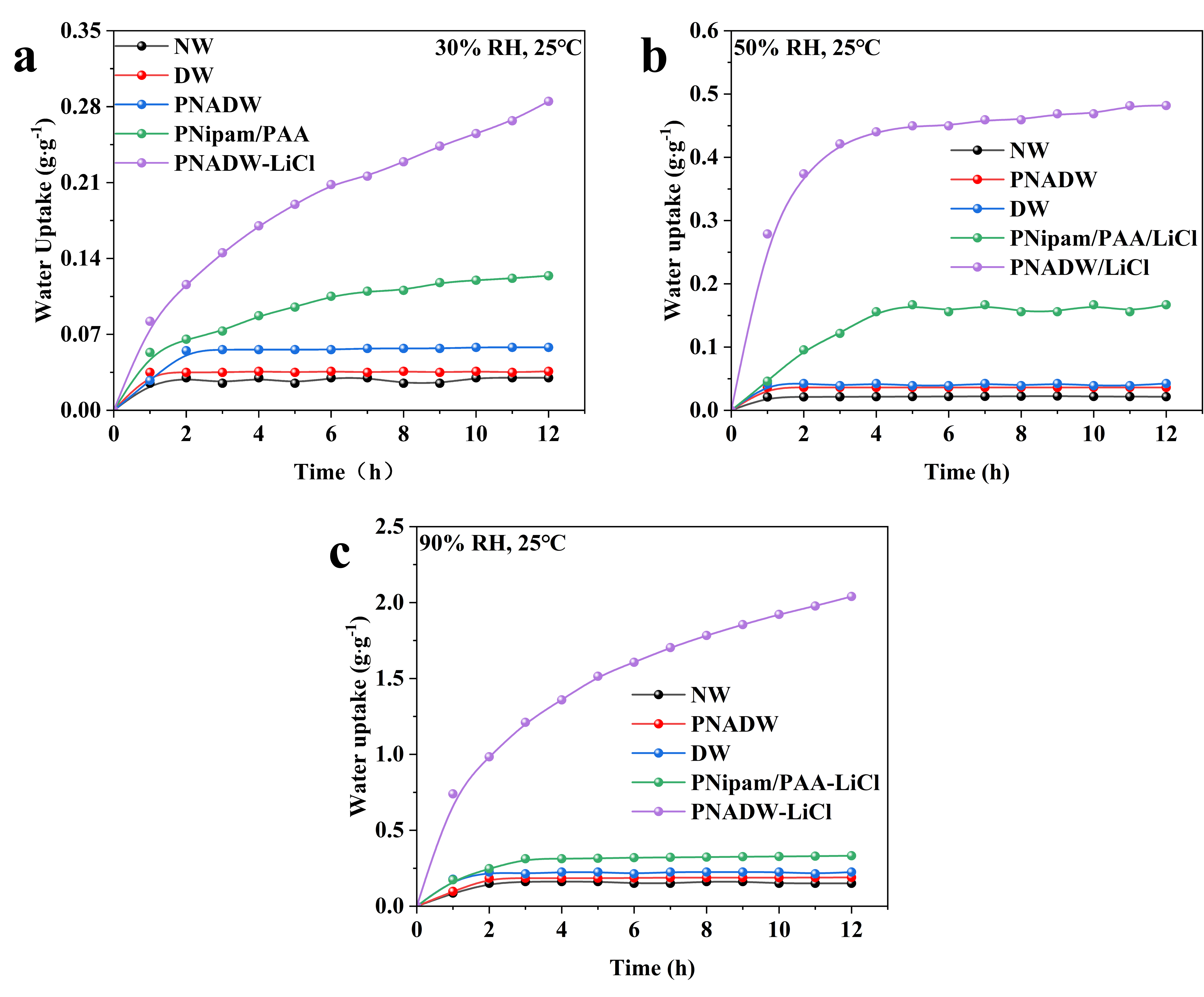

Supplement: Supplementary file 2 — Supporting File 2: advs75248‐sup‐0002‐FigureS1‐S20.zip [file ADVS-9999-e75248-s001.zip › Figure S10.tif]

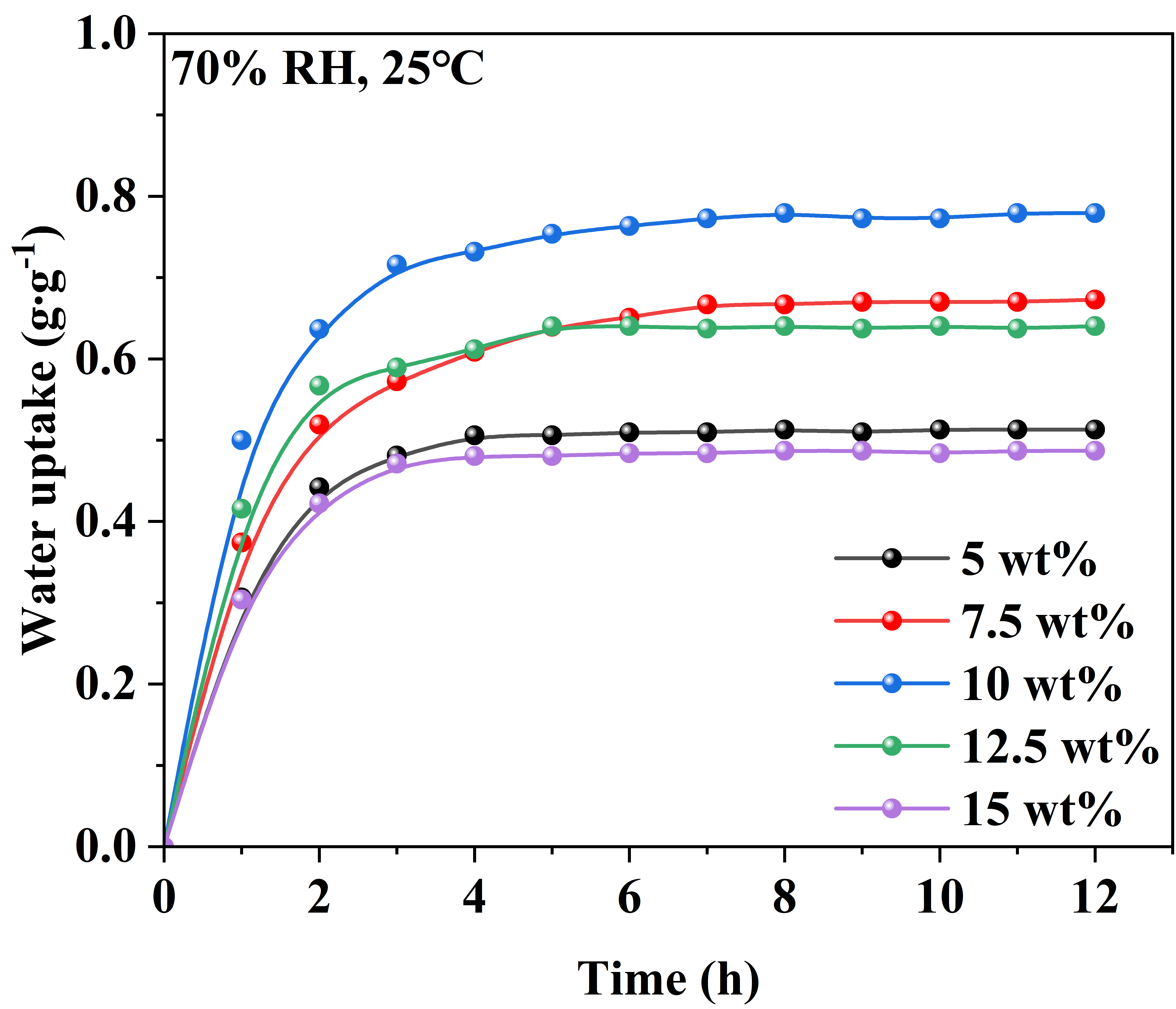

Supplement: Supplementary file 2 — Supporting File 2: advs75248‐sup‐0002‐FigureS1‐S20.zip [file ADVS-9999-e75248-s001.zip › Figure S11.tif]

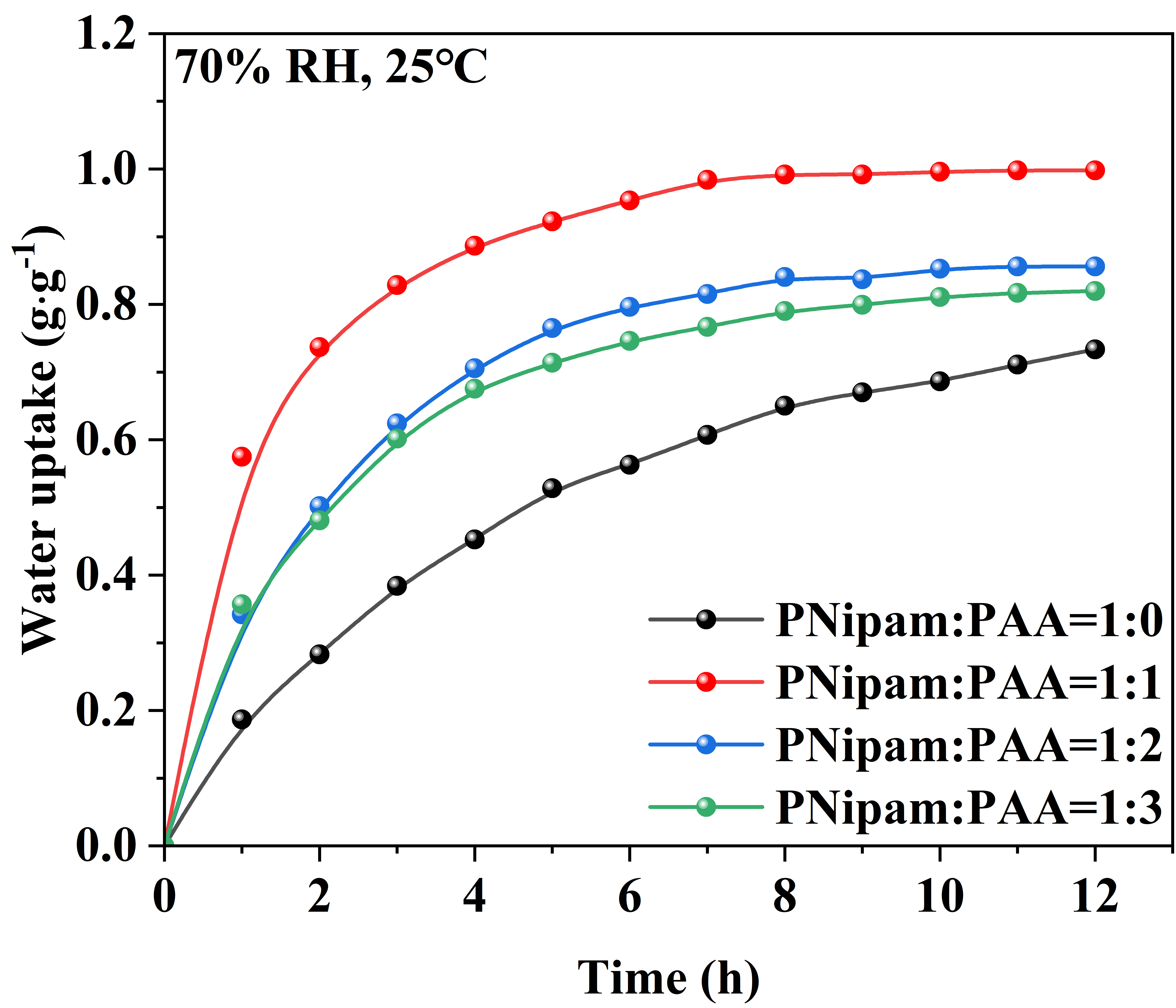

Supplement: Supplementary file 2 — Supporting File 2: advs75248‐sup‐0002‐FigureS1‐S20.zip [file ADVS-9999-e75248-s001.zip › Figure S12.tif]

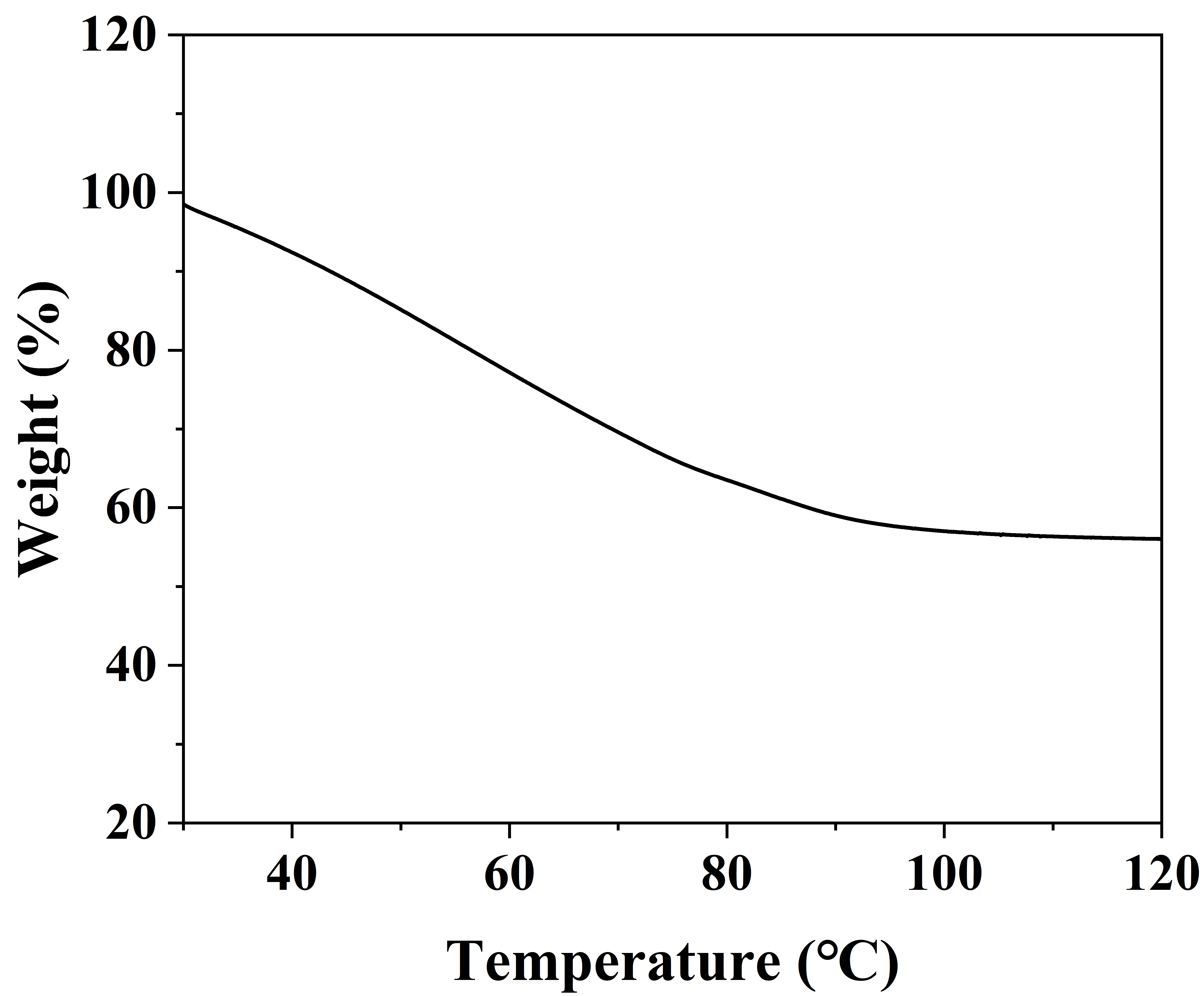

Supplement: Supplementary file 2 — Supporting File 2: advs75248‐sup‐0002‐FigureS1‐S20.zip [file ADVS-9999-e75248-s001.zip › Figure S13.tif]

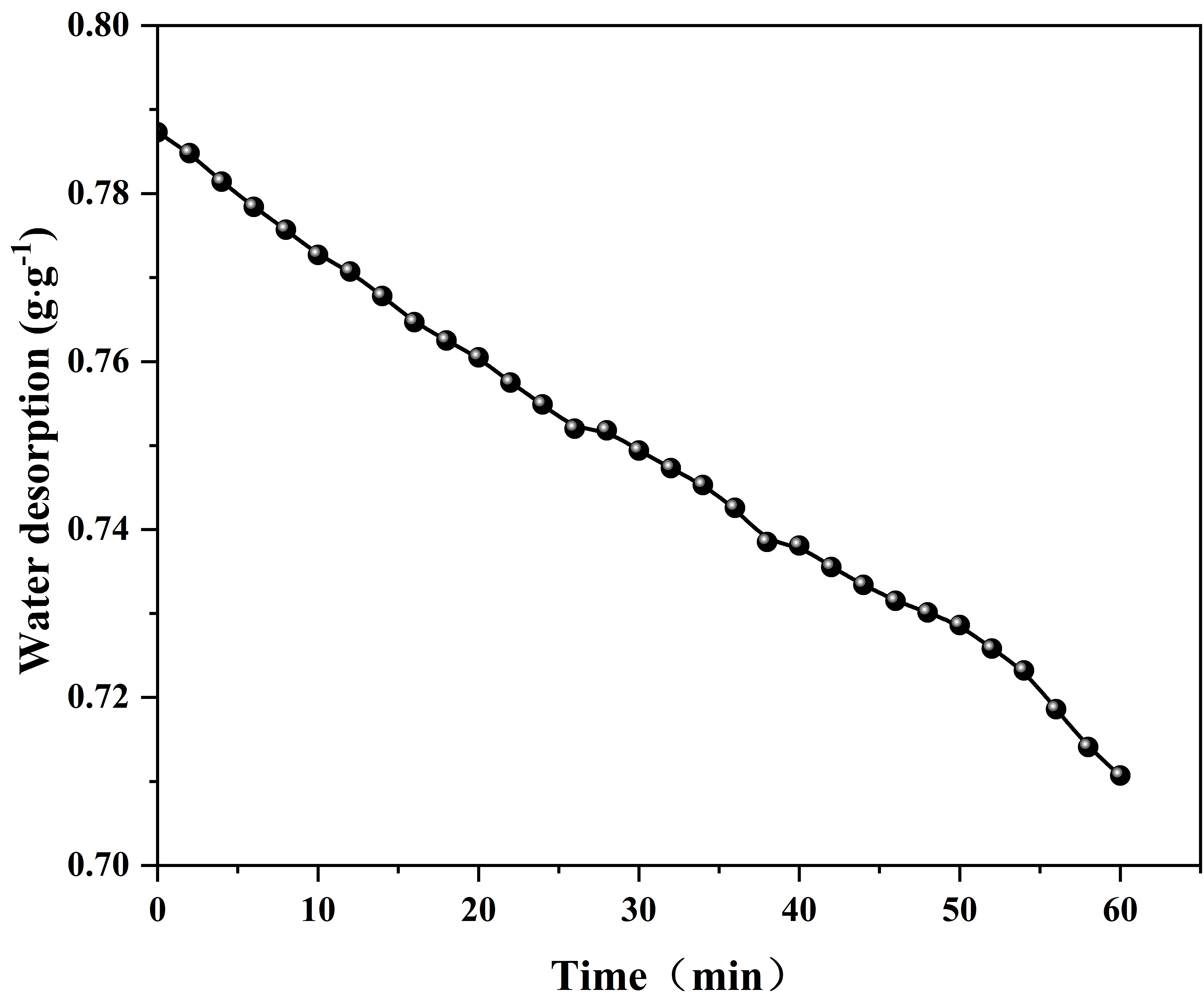

Supplement: Supplementary file 2 — Supporting File 2: advs75248‐sup‐0002‐FigureS1‐S20.zip [file ADVS-9999-e75248-s001.zip › Figure S14.tif]

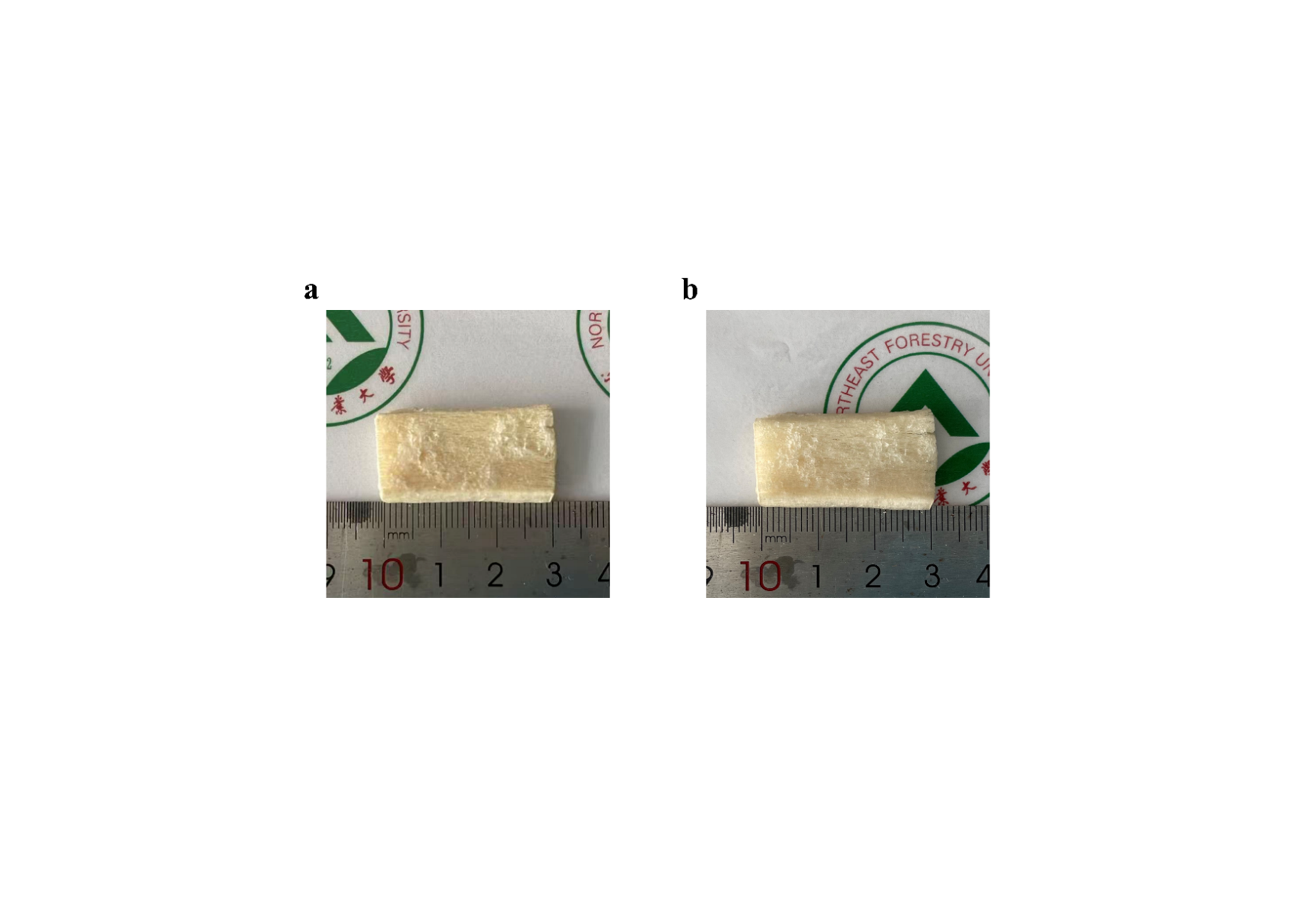

Supplement: Supplementary file 2 — Supporting File 2: advs75248‐sup‐0002‐FigureS1‐S20.zip [file ADVS-9999-e75248-s001.zip › Figure S15.tif]

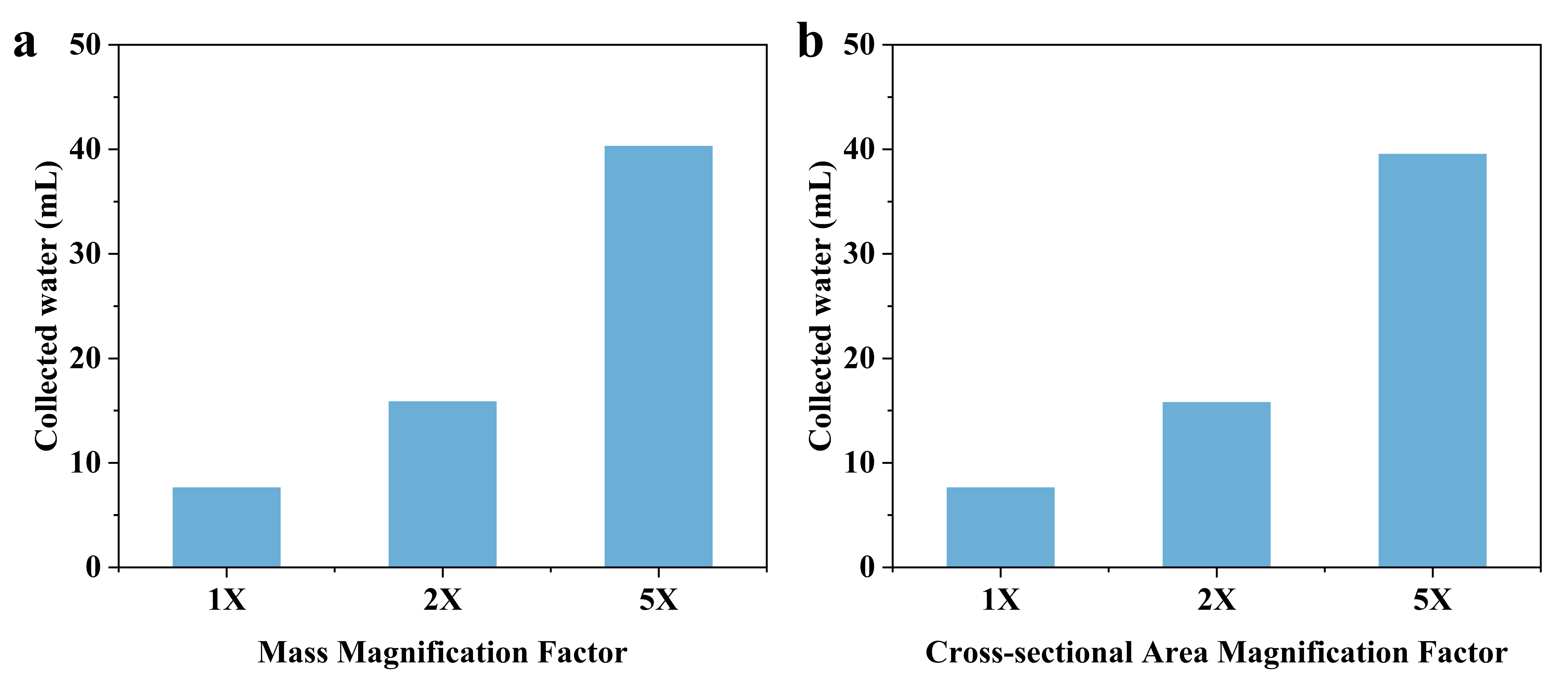

Supplement: Supplementary file 2 — Supporting File 2: advs75248‐sup‐0002‐FigureS1‐S20.zip [file ADVS-9999-e75248-s001.zip › Figure S16.tif]

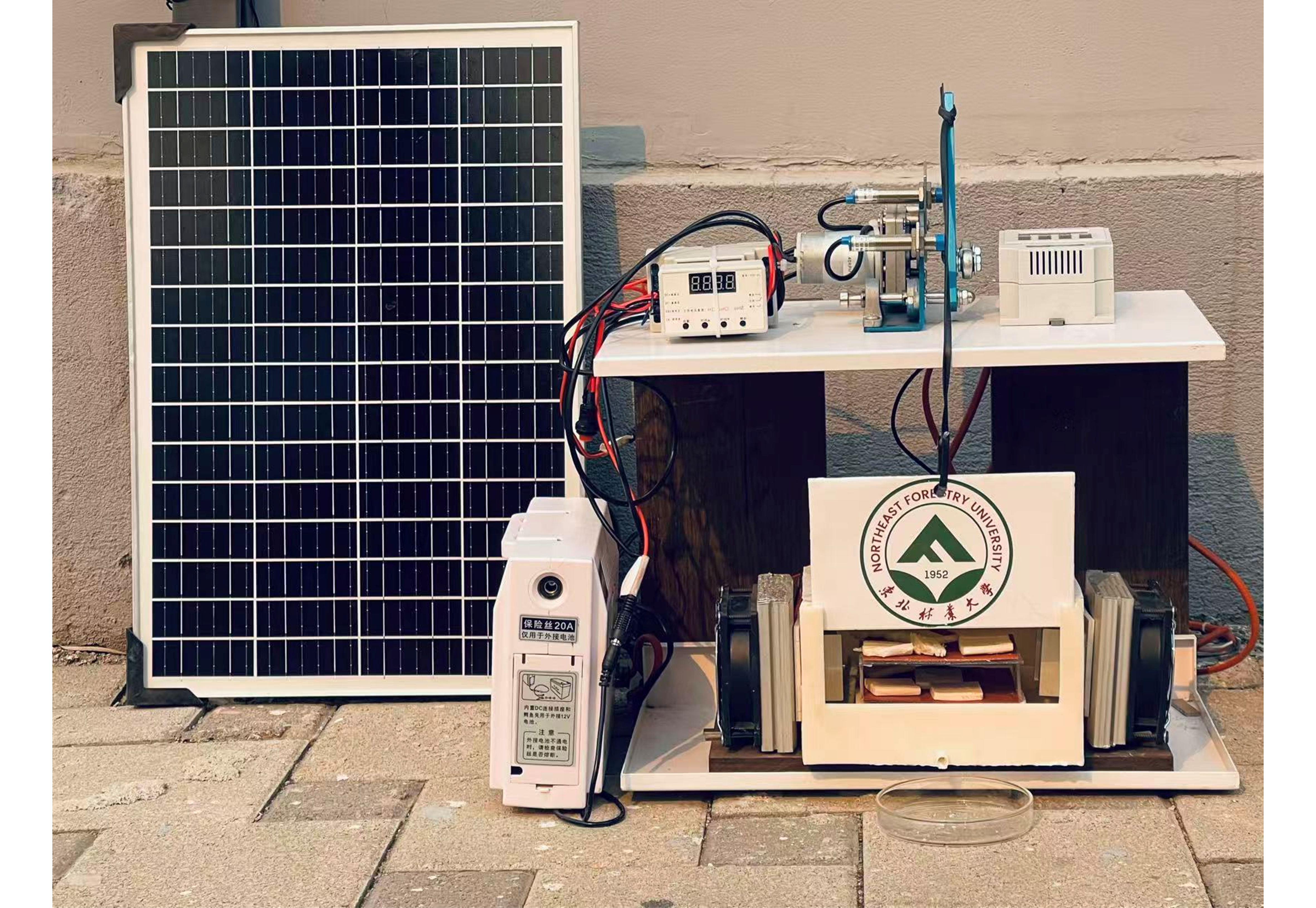

Supplement: Supplementary file 2 — Supporting File 2: advs75248‐sup‐0002‐FigureS1‐S20.zip [file ADVS-9999-e75248-s001.zip › Figure S17.tif]

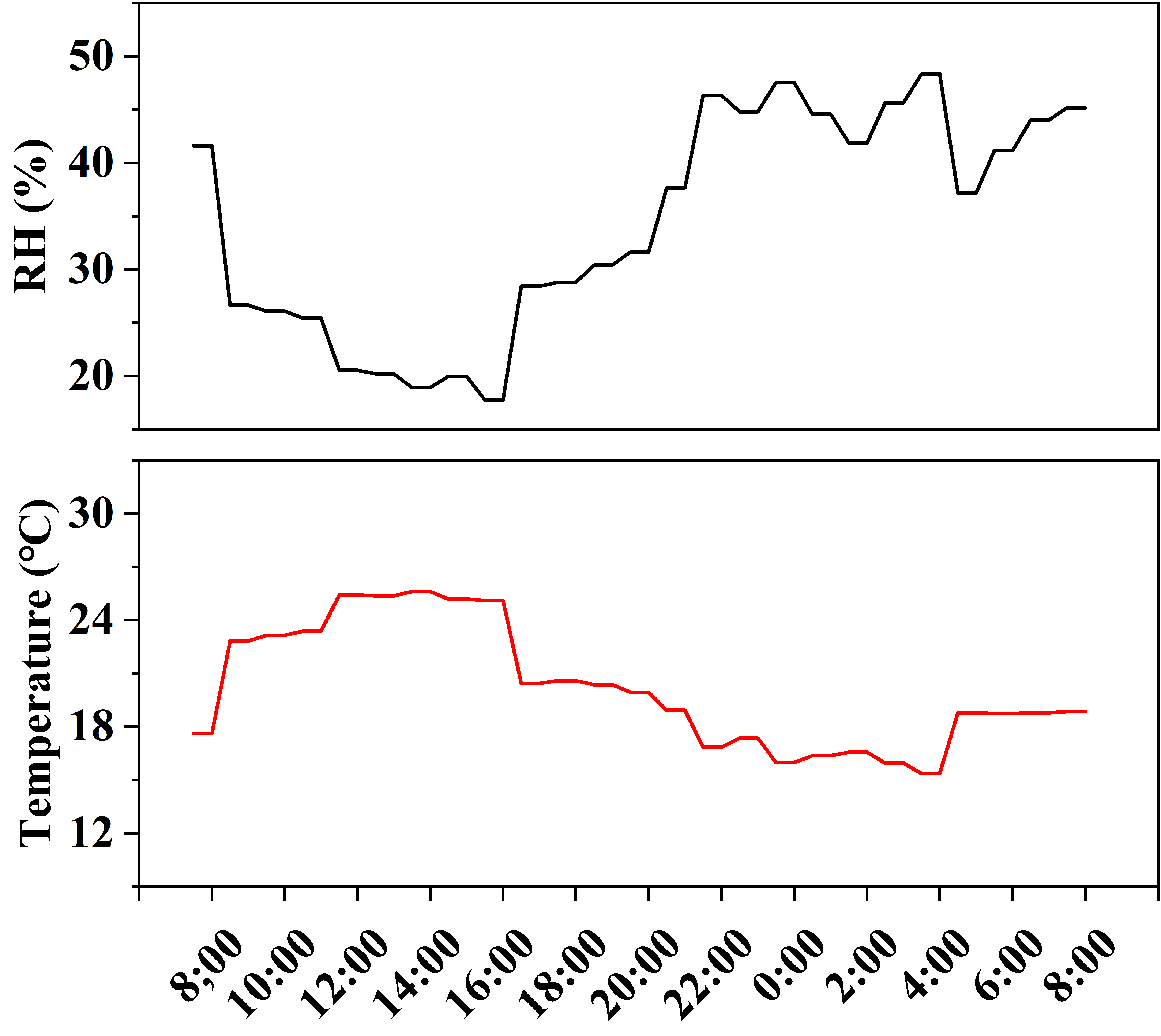

Supplement: Supplementary file 2 — Supporting File 2: advs75248‐sup‐0002‐FigureS1‐S20.zip [file ADVS-9999-e75248-s001.zip › Figure S18.tif]

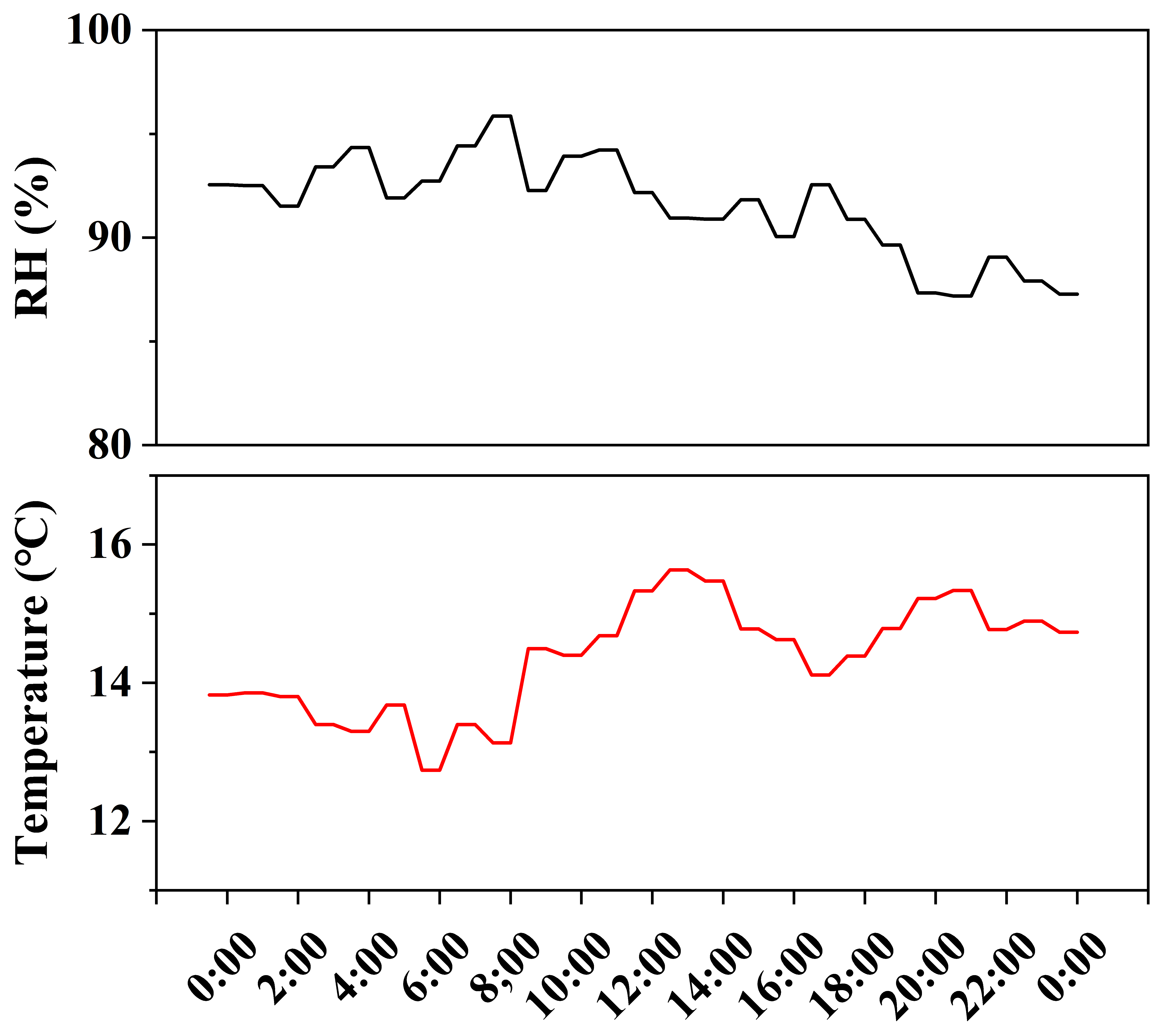

Supplement: Supplementary file 2 — Supporting File 2: advs75248‐sup‐0002‐FigureS1‐S20.zip [file ADVS-9999-e75248-s001.zip › Figure S19.tif]

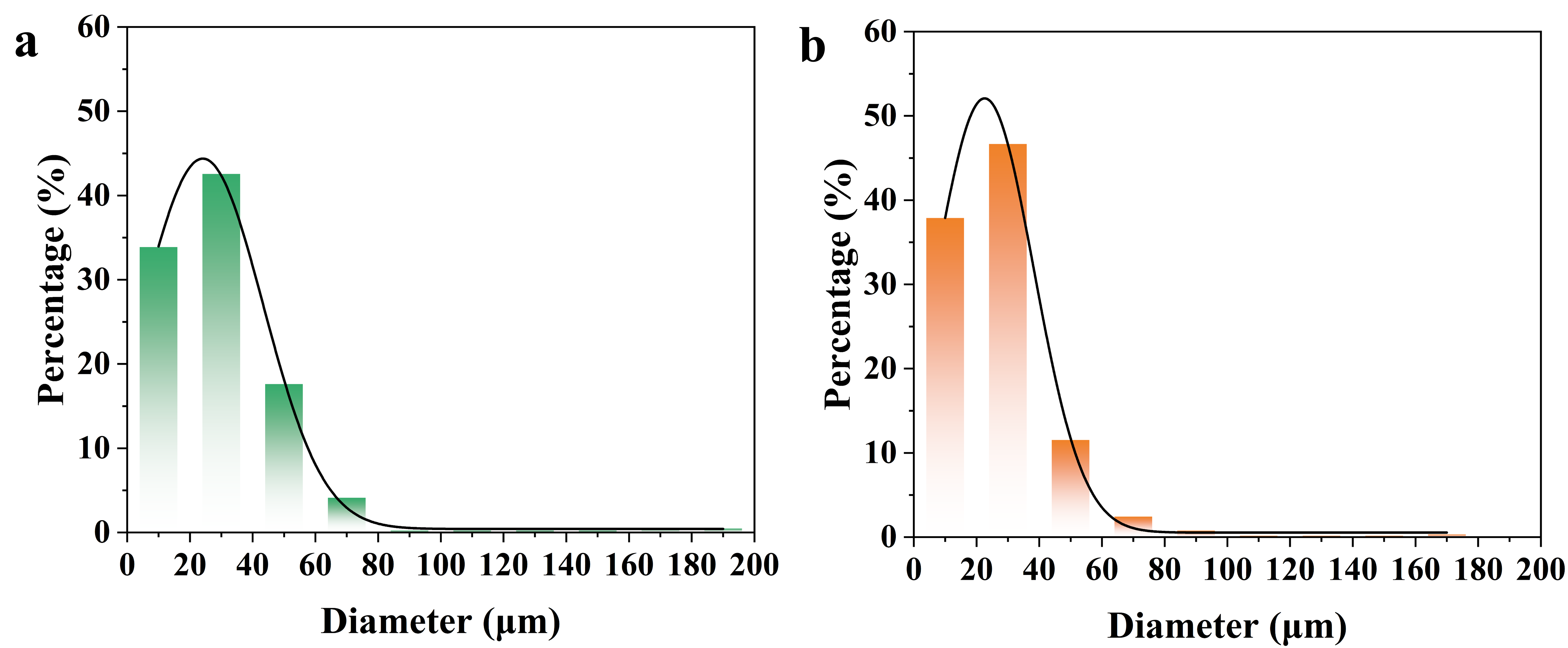

Supplement: Supplementary file 2 — Supporting File 2: advs75248‐sup‐0002‐FigureS1‐S20.zip [file ADVS-9999-e75248-s001.zip › Figure S2.tif]

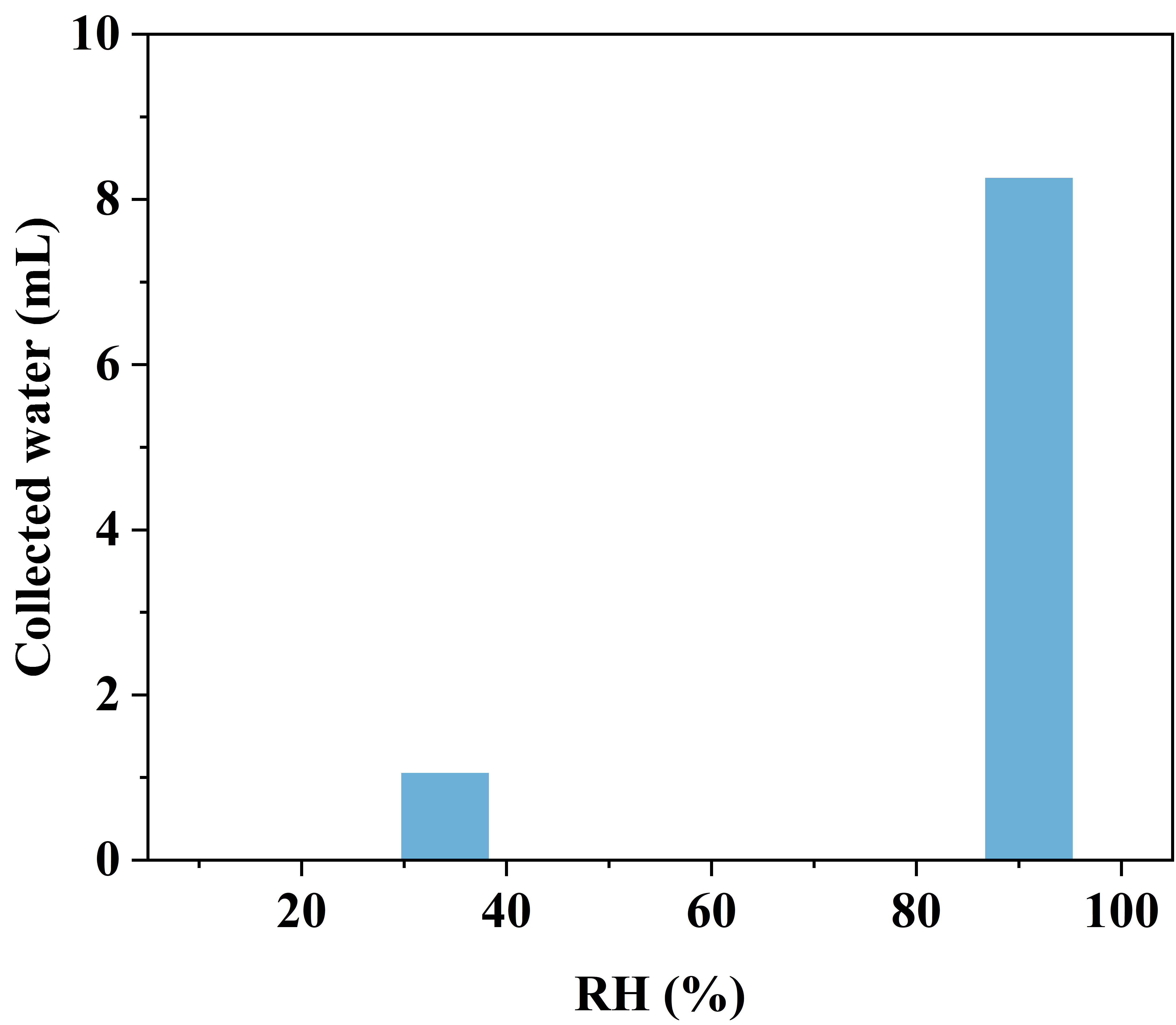

Supplement: Supplementary file 2 — Supporting File 2: advs75248‐sup‐0002‐FigureS1‐S20.zip [file ADVS-9999-e75248-s001.zip › Figure S20.tif]

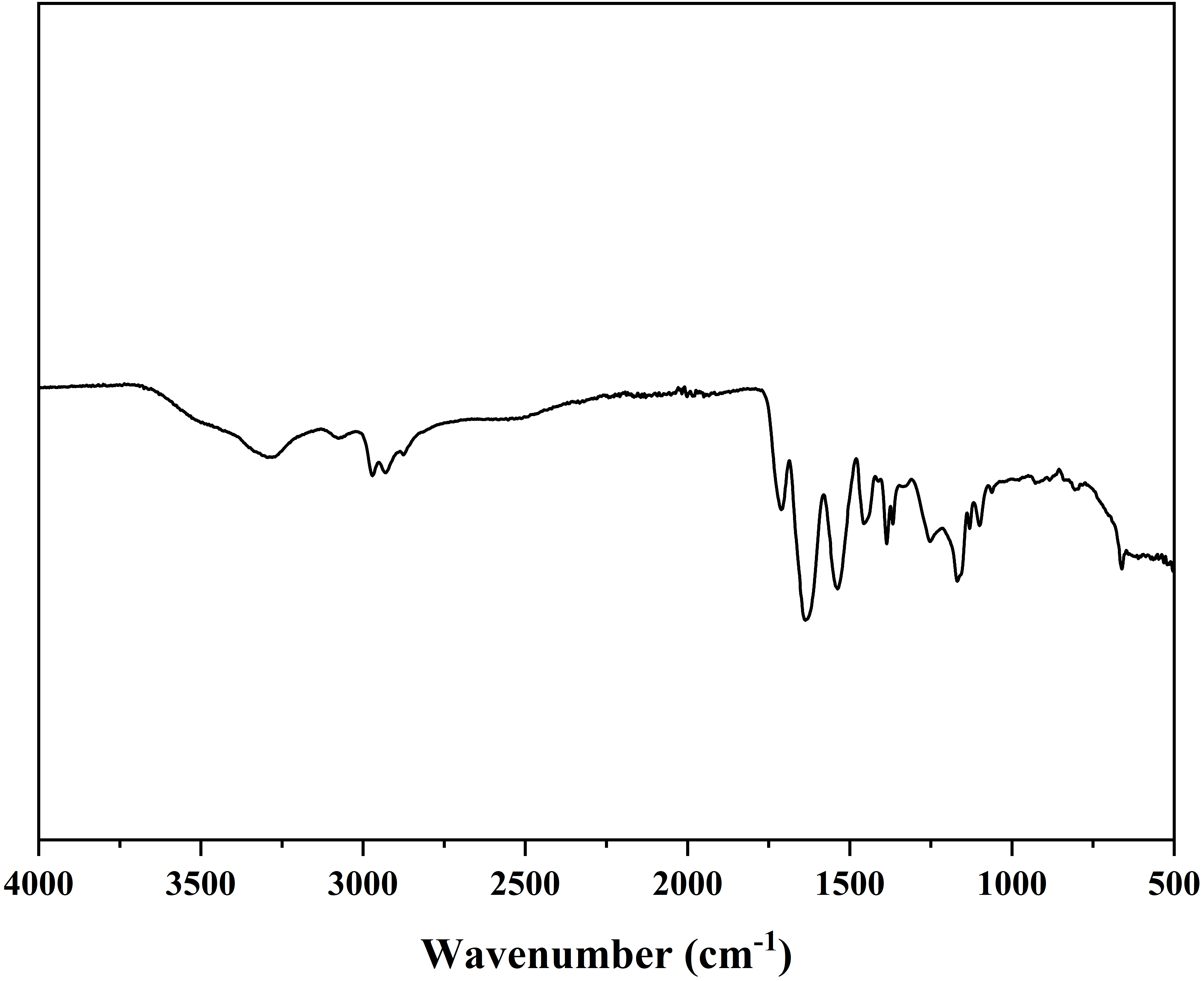

Supplement: Supplementary file 2 — Supporting File 2: advs75248‐sup‐0002‐FigureS1‐S20.zip [file ADVS-9999-e75248-s001.zip › Figure S3.tif]

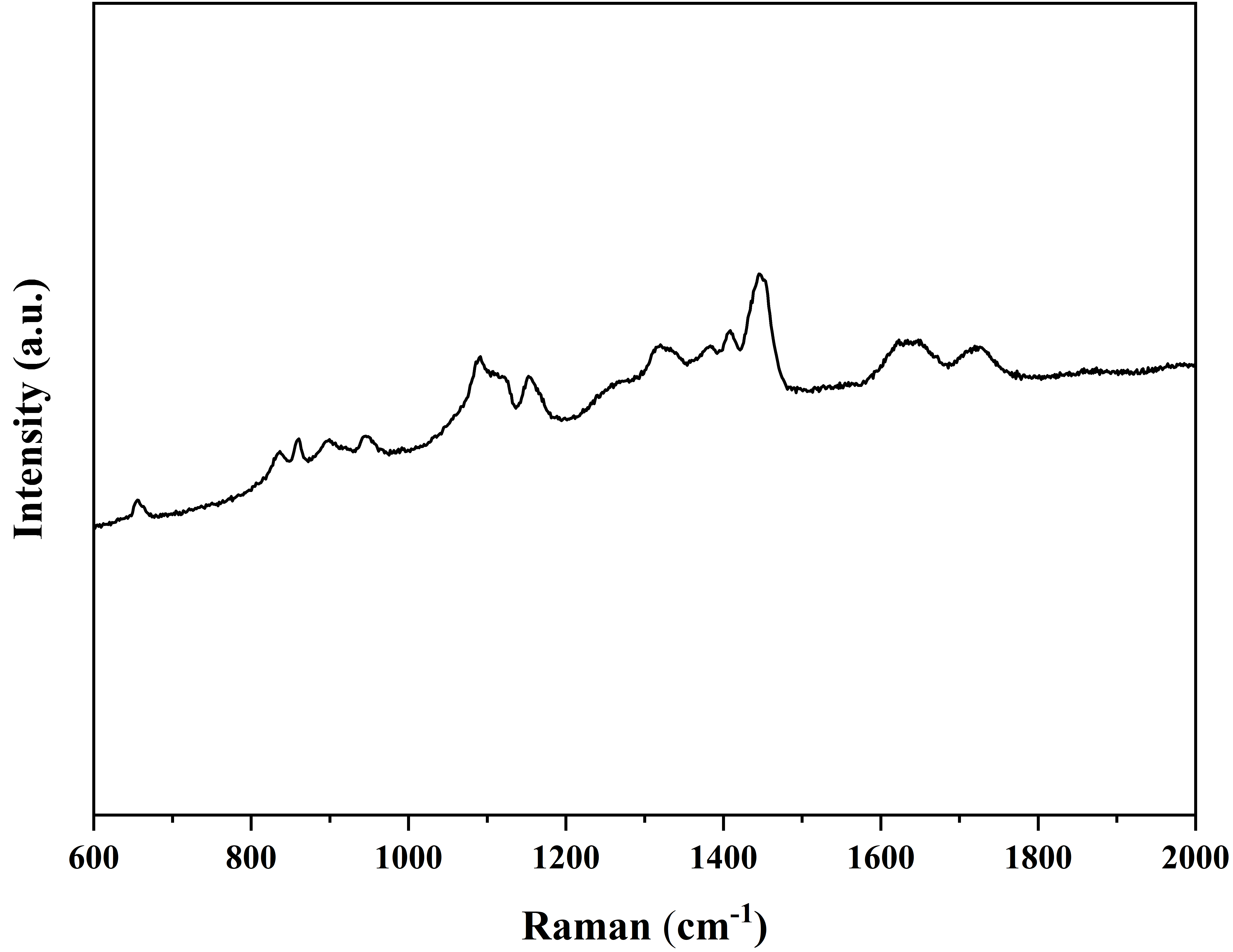

Supplement: Supplementary file 2 — Supporting File 2: advs75248‐sup‐0002‐FigureS1‐S20.zip [file ADVS-9999-e75248-s001.zip › Figure S4.tif]

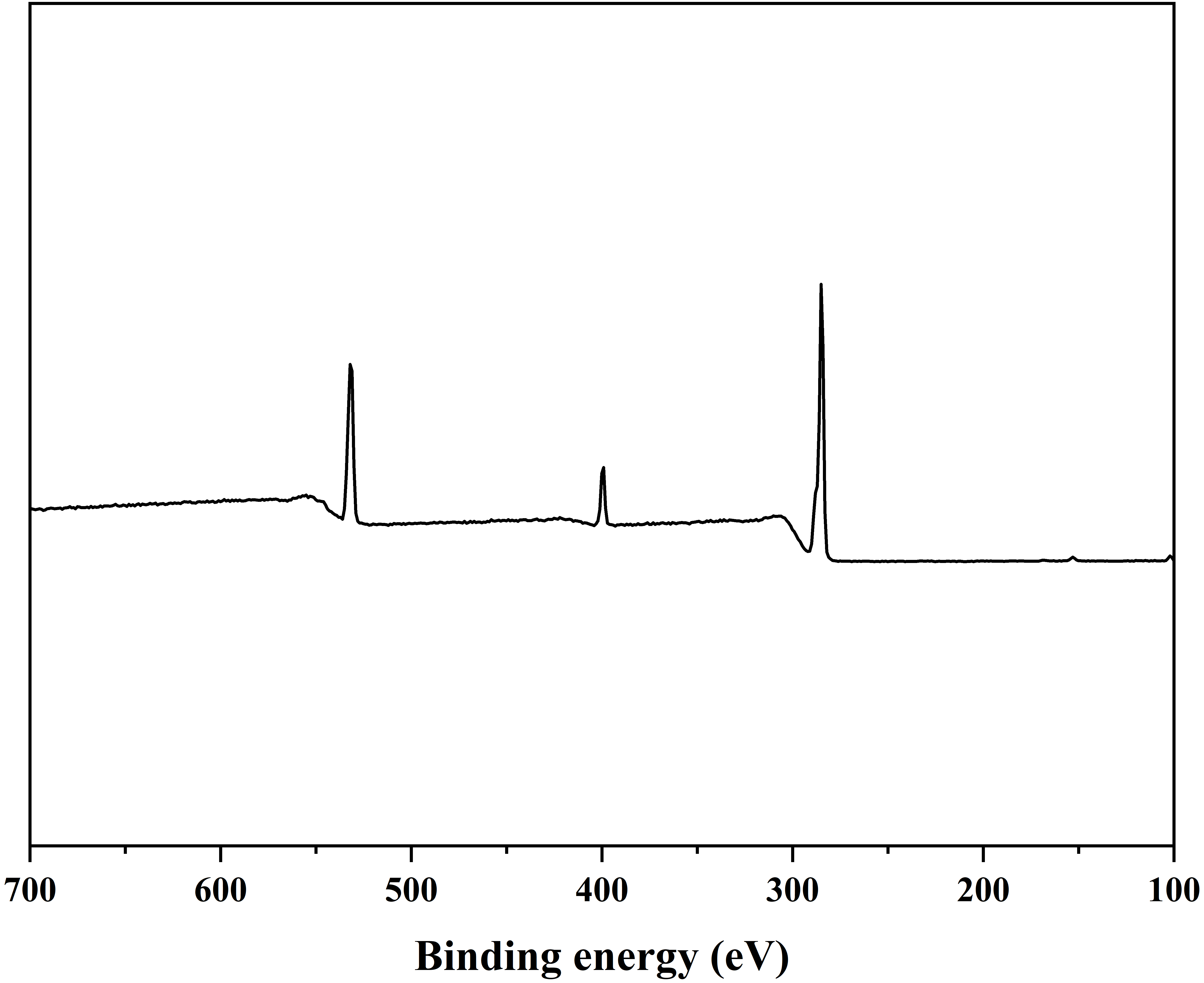

Supplement: Supplementary file 2 — Supporting File 2: advs75248‐sup‐0002‐FigureS1‐S20.zip [file ADVS-9999-e75248-s001.zip › Figure S5.tif]

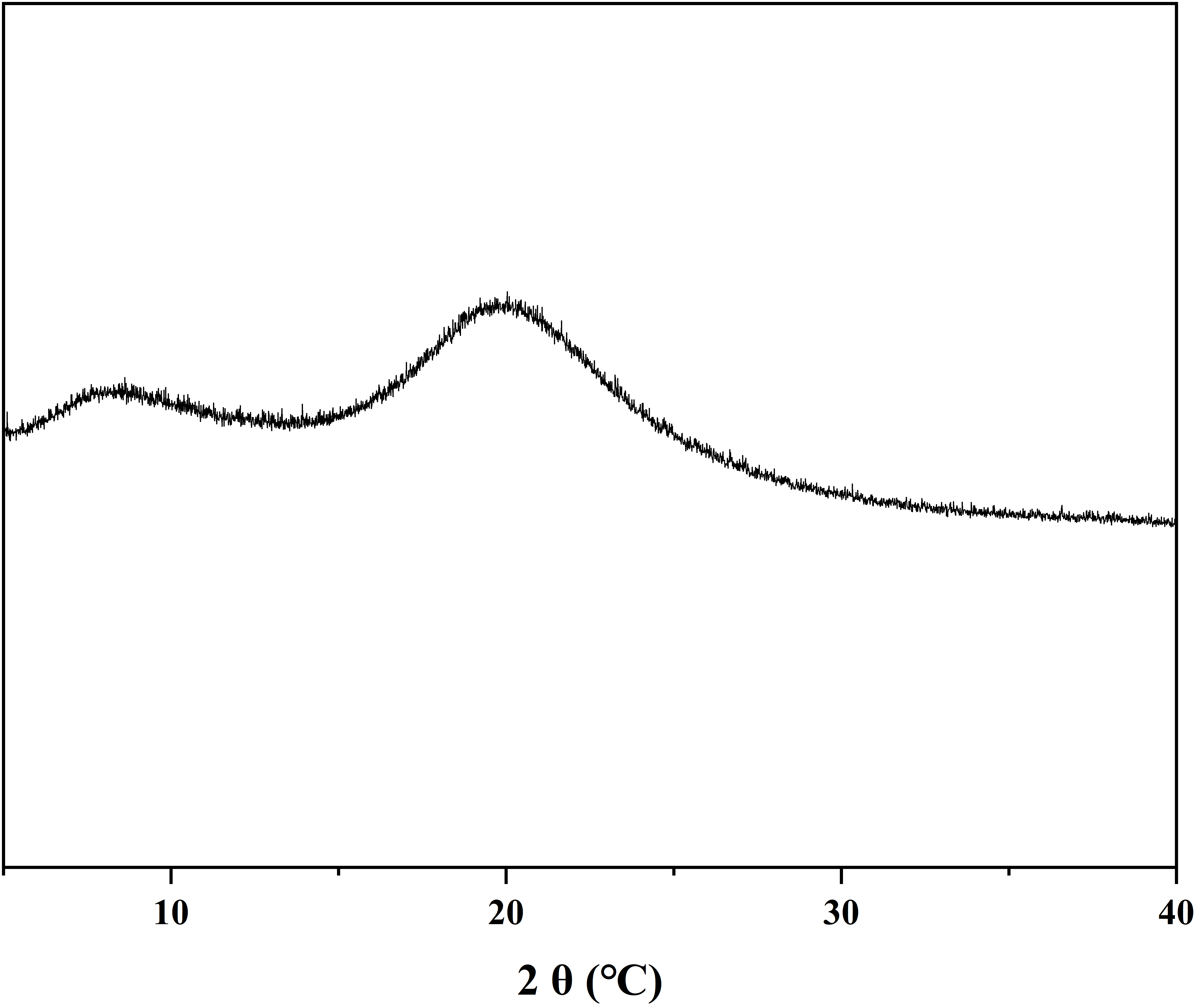

Supplement: Supplementary file 2 — Supporting File 2: advs75248‐sup‐0002‐FigureS1‐S20.zip [file ADVS-9999-e75248-s001.zip › Figure S6.tif]

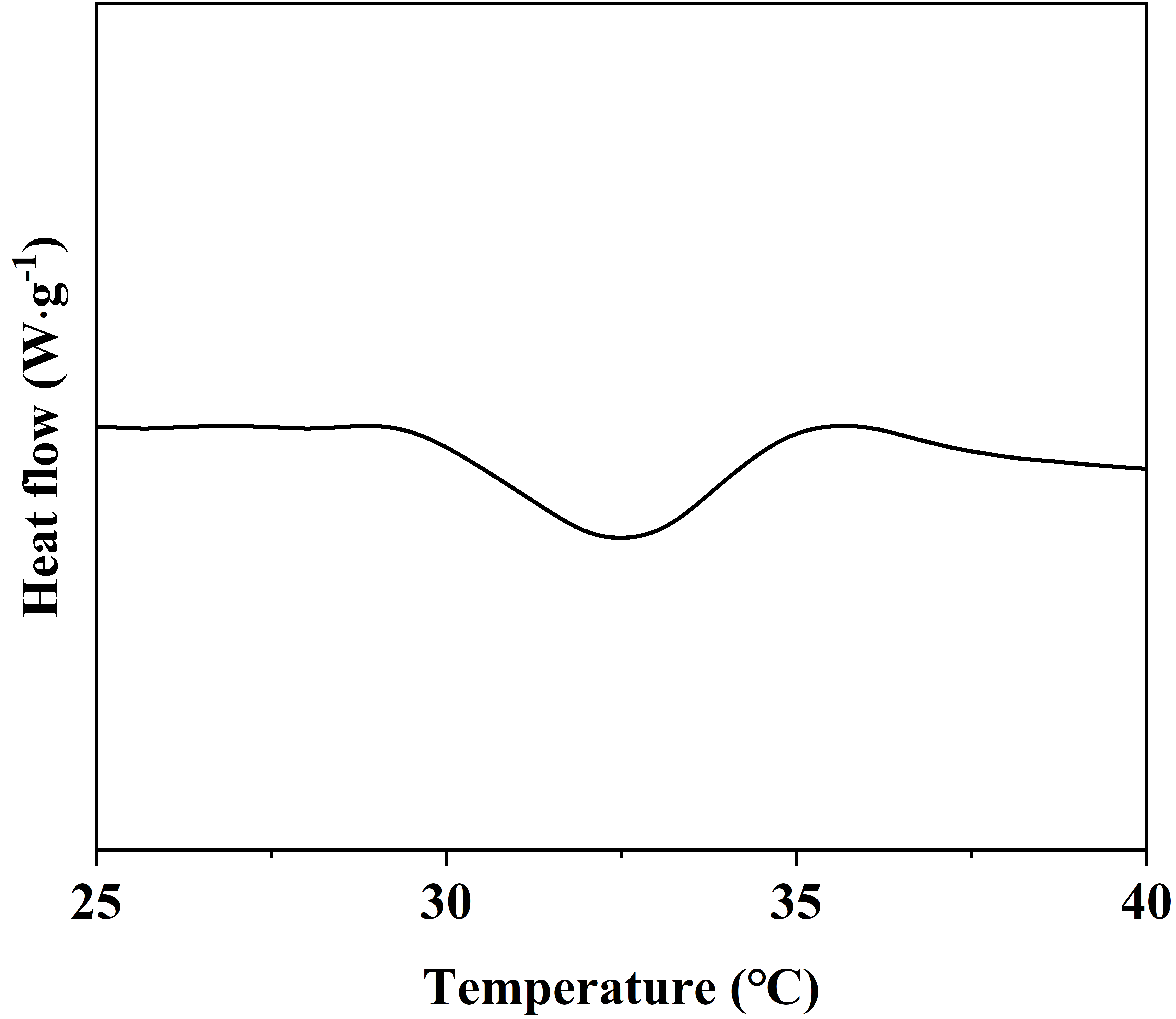

Supplement: Supplementary file 2 — Supporting File 2: advs75248‐sup‐0002‐FigureS1‐S20.zip [file ADVS-9999-e75248-s001.zip › Figure S7.tif]

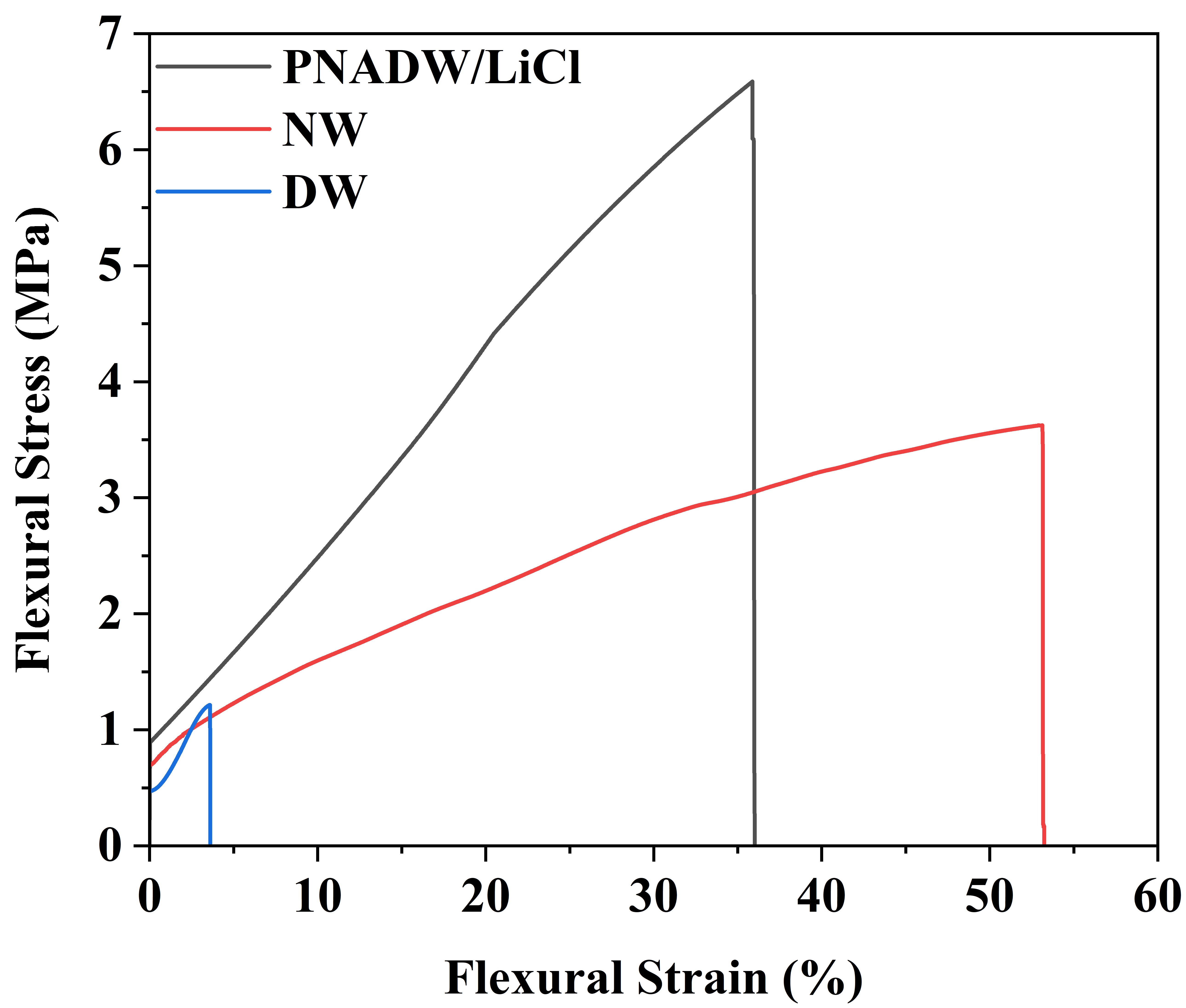

Supplement: Supplementary file 2 — Supporting File 2: advs75248‐sup‐0002‐FigureS1‐S20.zip [file ADVS-9999-e75248-s001.zip › Figure S8.tif]

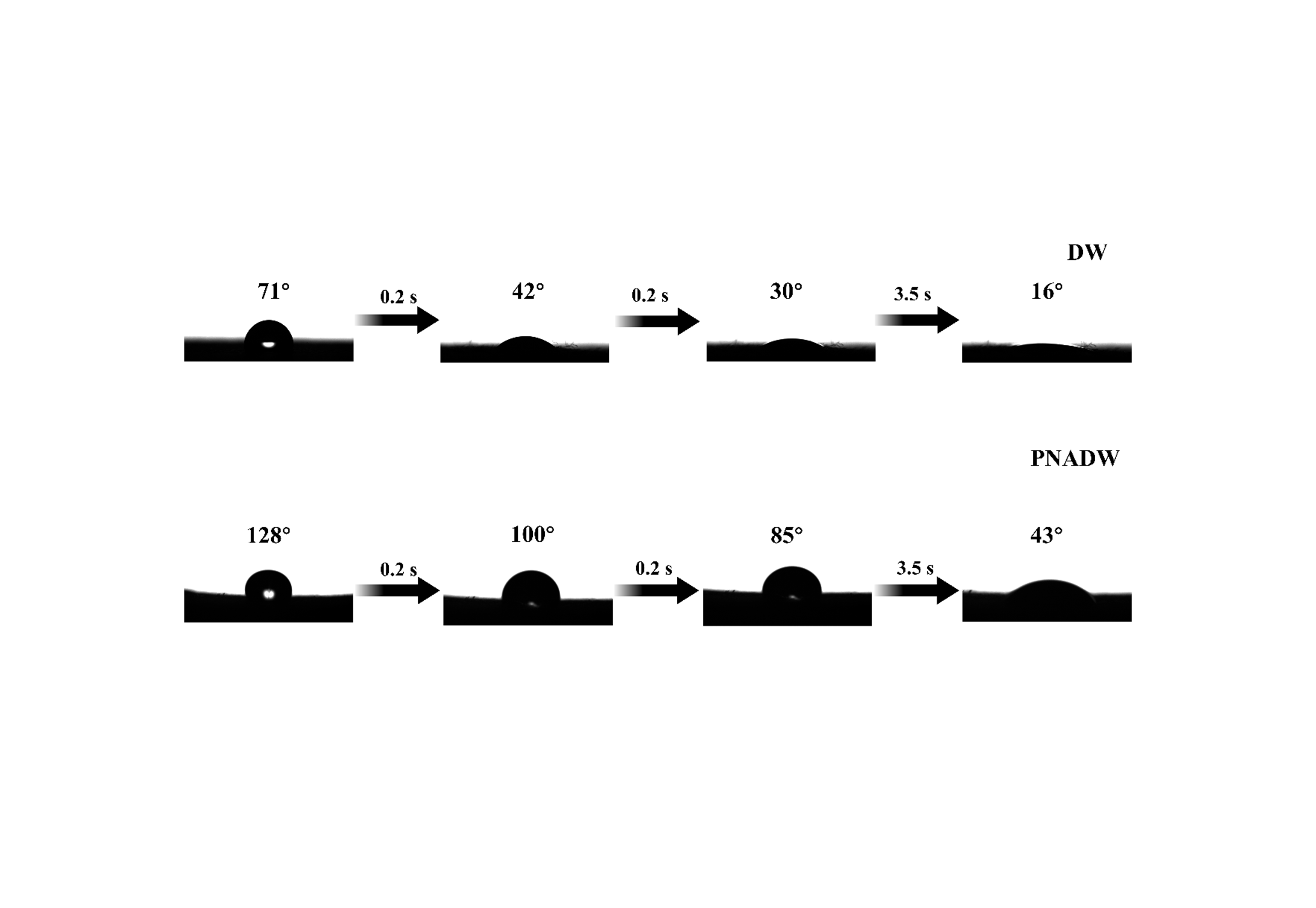

Supplement: Supplementary file 2 — Supporting File 2: advs75248‐sup‐0002‐FigureS1‐S20.zip [file ADVS-9999-e75248-s001.zip › Figure S9.tif]
